# Introducing novel potent anticancer agents of *1H*-benzo[*f*]chromene scaffolds, targeting *c-Src* kinase enzyme with MDA-MB-231 cell line anti-invasion effect

**DOI:** 10.1080/14756366.2018.1476503

**Published:** 2018-06-20

**Authors:** Hany E. A. Ahmed, Mohammed A. A. El-Nassag, Ahmed H. Hassan, Rawda M. Okasha, Saleh Ihmaid, Ahmed M. Fouda, Tarek H. Afifi, Ateyatallah Aljuhani, Ahmed M. El-Agrody

**Affiliations:** a Department of Pharmacy College, Pharmacognosy and Pharmaceutical Chemistry, Taibah University, Al-Madinah Al-Munawarah, Saudi Arabia;; b Pharmaceutical Organic Chemistry Department, Faculty of Pharmacy, Al-Azhar University, Cairo, Egypt;; c Chemistry Department, Faculty of Science, Al-Azhar University, Cairo, Egypt;; d Chemistry Department, Faculty of Science, Jazan University, Jazan, Saudi Arabia;; e Chemistry Department, Faculty of Science, Taibah University, Al-Madinah Al-Munawarah, Saudi Arabia;; f Chemistry Department, Faculty of Science, King Khalid University, Abha, Saudi Arabia

**Keywords:** Microwave synthesis, 1*H*-benzo[*f*]chromenes, antitumour activity, SAR study, Caspase 3/7

## Abstract

In our effort to develop novel and powerful agents with anti-proliferative activity, two new series of 1*H*-benzo[*f*]chromene derivatives, **4a–h** and **6a–h**, were synthesised using heterocyclocondensation methodologies under microwave irradiation condition. The structures of the target compounds were established on the basis of their spectral data, IR, ^1^H NMR, ^13^ C NMR, ^13^ C NMR-DEPT/APT, and MS data. The new compounds have been examined for their anti-proliferative activity against three cancer cell lines, MCF-7, HCT-116, and HepG-2. Vinblastine and Doxorubicin have been used as positive controls in the viability assay. The obtained results confirmed that most of the tested molecules revealed strong and selective cytotoxic activity against the three cancer cell lines. Moreover, these molecules exhibited weak cytotoxicity on the HFL-1 line, which suggested that they might be ideal anticancer candidates. The SAR study of the new benzochromene compounds verified that the substituents on the phenyl ring of 1*H*-benzo[*f*]chromene nucleus, accompanied with the presence of bromine atom or methoxy group at the 8-position, increases the ability of these molecules against the different cell lines. Due to their high anti-proliferative activity, compounds **4c** and **6e** were selected to be examined their proficiency to inhibit the invasiveness of the highly sensitive and invasive breast cancer cell line, MDA-MB-231. The anti-invasion behaviour of these molecules against the highly sensitive, non-oestrogen, and progesterone MDA-MB-231 cell line gave rise to their decreasing metastatic effect compared to the reference drug. Furthermore, this report explores the apoptotic mechanistic pathway of the cytotoxicity of the target compounds and reveals that most of these compounds enhance the Caspase 3/7 activity that could be considered as potential anticancer agents.

## 1. Introduction

**Figure 1.**
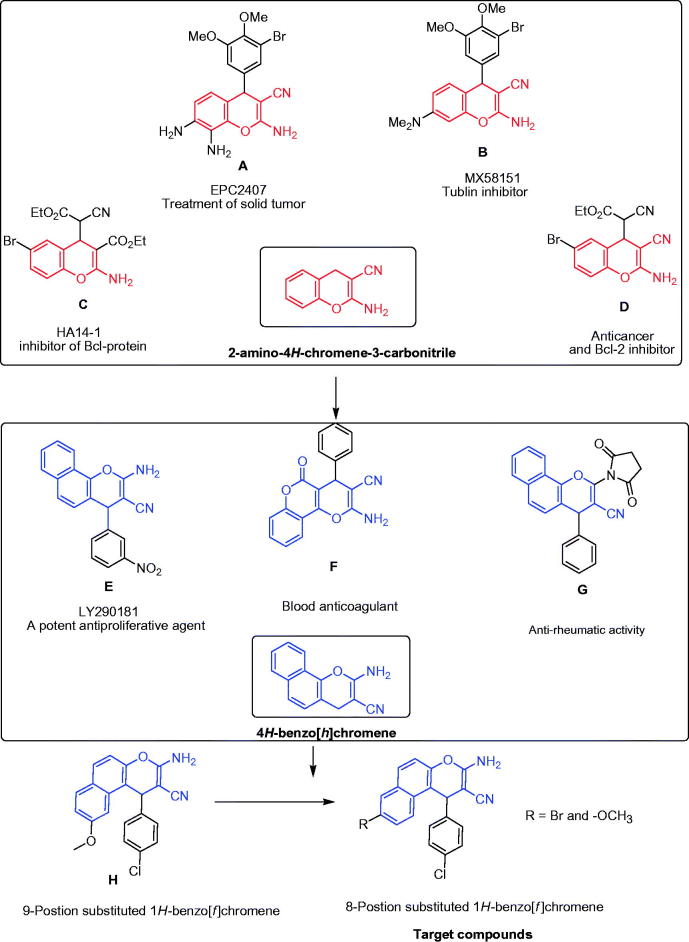
Reference drugs bearing chromene (red highlighted) or 1*H*-benzo[*f*]chromene (blue highlighted) with different biological activities.

Chromene and benzochromene molecules are notorious for exhibiting a wide range of biological and pharmacological activities such as antimicrobial^1–5^, antileishmanial^6^^,^^7^, antioxidant^8^^,^^9^, vascular-disrupting^10^, oestrogenic, anticoagulant and antispasmolytic^11^, blood platelet antiaggregating^12^, analgesic^13^, and hypolipidemic effects^14^. In addition, the current interest in chromenes and benzochromenes arises from their applications in the treatment of human diseases^15–21^. Furthermore, chromene derivatives have emerged as promising and desirable scaffolds in the development of potent antitumour agents^22^^,^^23^. For example, Crolibulin™ (**A**) is currently in phase I/II of clinical trials for the treatment of advanced solid tumours^15^, while 2-amino-4-(3-bromo-4,5-dimethoxyphenyl)-7-(dimethylamino)-4*H*-chromene-3-carbonitrile (**B**) has been known as a tubulin inhibitor^21^, and ethyl 2-amino-6-bromo-4-(1-cyano-2-ethoxy-2-oxoethyl)-4*H*-chromene-3-carboxylate (**C**) and 2-amino-6-bromo-4-(1-cyano-2-ethoxy-2-oxoethyl)-4*H*-chromene-3-carbonitrile (**D**) act as inhibitors of Bcl-2 protein and as apoptosis inducers^16^^,^^17^. It has also been cited in literature that the 4-aryl-4*H*-chromenes have been developed as future anticancer agents with potent apoptosis induction through cell-based mediated Caspases 3/7 activation^16^^,^^17^. Fallah et al. also reported the synthesis of 4-aryl-4*H*-chromenes with mild selective *c*-*Src* kinase anticancer activity^24^. Indeed, benzochromene is a successful choice in the treatment of several human diseases. For instance, 2-amino-4-(3-nitrophenyl)-4*H*-benzo[*h*]chromene-3-carbonitrile (**E**) is a potent anti-proliferative agent for a variety of cell types and acts as an inhibitor for mitosis and microtubules^25^^,^^26^. Meanwhile, 2-amino-5-oxo-4-phenyl-4,5-dihydropyrano[3,2-*c*]chromene-3-carbonitrile (**F**) serves as a precursor for the blood anticoagulant warfarin^19^, and 4-substituted-2-(*N*-succinimido)-4*H*-benzo[*h*]chromene-3-carbonitriles (**G**) displays anti-rheumatic activity^20^ as shown in Figure 1.

In our efforts to be a part of the development process of novel cytotoxic agents, we have recently focussed on utilising the 1*H*-benzo[*f*]chromene scaffold with methoxy substituent at the 9-position to create various potent and high anti-proliferative compounds such as 3-amino-1-(4-chlorophenyl)-9-methoxy-1*H*-benzo[*f*]chromene-2-carbonitrile^27^ (**H**). Since it was revealed that the position and the type of the substituent have a great effect on the cytotoxic activity of the desired compounds, the main goal of the present study is to synthesise a new family of substituted 1*H*-benzo[*f*]chromene at the 8-postion with electron-withdrawing and electron-donating groups. The rational design of the new compounds was based on the following considerations: (i) the 1*H*-benzo[*f*]chromene scaffold itself, (ii) the presence of the effective 8-postion substitution, and (iii) the type of the aryl groups linked to chromene scaffold which seems to play a crucial role in the cytotoxic activity.

Herein, we introduced the microwave irradiation synthesis of the 3-amino-1*H-*benzo[*f*]chromene-2-carbonitrile derivatives. The target compounds were examined for their anti-proliferative activity towards three cancer cell lines, MCF-7, HCT-116, and HepG-2 and the viability assay was evaluated against two standard drugs, Vinblastine and Doxorubicin. The Structure-Activity Relationships of these molecules elucidated the effect of the substituents at the C-1 and C-8 positions of the 1*H*-benzo[*f*]chromene moiety on their cytotoxic activity.

## 2. Experimental section

### 2.1. Materials and equipments

All chemicals were purchased from Sigma-Aldrich Chemical Co. (Sigma-Aldrich Corp., St. Louis, MO). All melting points were measured with a Stuart Scientific Co. Ltd apparatus are uncorrected. The IR spectra were recorded on a KBr disc on a Jasco FT/IR 460 plus spectrophotometer. The ^1^H NMR (500 MHz) and ^13^C NMR (125 MHz) spectra were measured on BRUKER AV 500 MHz spectrometer in DMSO-d_6_ as a solvent, using tetramethylsilane (TMS) as an internal standard, and chemical shifts were expressed as *δ* (ppm). The ^13^C NMR spectra were obtained using distortion less enhancement by polarisation transfer (DEPT), where the signals of CH and CH_3_ carbon atoms appear normal (up), and the signals of carbon atoms in CH_2_ environments appear negative (down). The ^13^C N MR spectra were also obtained using attached proton test (APT), with this technique, the signals of CH and CH_3_ carbon atoms appear normal (up) and the signal of CH_2_ and Cq environments appears negative (down). The Microwave apparatus used is Milestone Sr1, Microsynth. The mass spectra were determined on a Shimadzu GC/MS-QP5050A spectrometer. Elemental analysis was carried out at the Regional Centre for Mycology and Biotechnology (RCMP), Al-Azhar University, Cairo, Egypt, and the results were within ±0.25%. Reaction courses and product mixtures were routinely monitored by thin layer chromatography (TLC) on silica gel precoated F_254_ Merck plates.

### 2.2. General procedure for synthesis of 1*H*-benzo[*f*]chromenes 4a–h and 6a–h

A reaction mixture of 6-bromo-2-naphthol **1** or 6-methoxy-2-naphthol **5** (0.01 mol), different aromatic aldehydes **2a–h** (0.01 mol), malononitrile **3** (0.01 mol) and piperidine (0.5 ml) in ethanol (30 ml) was heated under Microwave irradiation conditions for 2 min at 140 °C. After completion of the reaction, the reaction mixture was cooled to room temperature and the precipitated solid was filtered off, washed with methanol, and was recrystallised from ethanol or ethanol/benzene. The physical and spectral data of compounds **4a–h** and **6a–h** are as follows:

#### 2.2.1. 3-Amino-8-bromo-1-phenyl-1*H*-benzo[*f*]chromene-2-carbonitrile 4a

Colourless crystals from ethanol; yield 89%; m.p. 245–246 °C (Lit. m. p. 240 °C^28^); IR (KBr) *υ* (cm^−1^): 3477, 3329, 3213 (NH_2_), 2204 (CN); ^1^H NMR *δ*: 8.21–7.15 (m, 10H, aromatic), 7.04 (bs, 2H, NH_2_, D_2_O exchangeable), 5.32 (s, 1H, H-1); ^13^C NMR *δ*: 159.53 (C-3), 147.12 (C-4a), 132.12 (C-10a), 130.24 (C-7), 129.85 (C-9), 128.73 (C-6), 128.51 (C-6a), 126.67 (C-10), 125.92 (C-10b), 120.31 (C-5), 118.12 (CN), 116.01 (C-8), 57.78 (C-2), 37.89 (C-1), 145.42, 128.84,128.75, 126.92 (aromatic); *m*/*z* (%): 378 (M^+^+2_,_ 18.99), 376 (M^+^, 57.92) with a base peak at 299 (100); Anal. Calcd for C_20_H_13_BrN_2_O: C, 63.68; H, 3.47; N, 7.43. Found: 63.79; H, 3.59; N, 7.55%.

#### 2.2.2. 3-Amino-8-bromo-1-(4-fluorophenyl)-1*H*-benzo[*f*]chromene-2-carbonitrile 4b

Colourless crystals from ethanol; yield 88%; m.p. 242–243 °C; IR (KBr) *υ* (cm^−1^): 3457, 3337, 3201 (NH_2_), 2203 (CN); ^1^H NMR *δ*: 8.22–7.08 (m, 9H, aromatic), 7.04 (bs, 2H, NH_2_), 5.37 (s, 1H, H-1); ^13^C NMR *δ*: 159.52 (C-3), 147.07 (C-4a), 132.15 (C-10a), 130.28 (C-7), 129.90 (C-9), 128.76 (C-6), 128.28 (C-6a), 125.89 (C-10), 120.21 (C-10b), 118.16 (CN), 118.15 (C-5), 115.82 (C-8), 57.65 (C-2), 37.05 (C-1), 161.79, 141.68, 128.87, 128.83, 115.56, 115.39 (aromatic); ^13^C NMR-DEPT spectrum at 135°CH, CH_3_ [positive (up)], CH_2_ [negative (down)], revealed the following signals at *δ*: 130.28 (C-7 ↑), 129.90 (C-9 ↑), 128.87 (aromatic ↑), 128.83 (aromatic ↑), 128.76 (C-6 ↑), 125.89 (C-10 ↑), 118.15 (C-5 ↑), 115.56 (aromatic ↑), 115.39 (aromatic ↑), 37.05 (C-1 ↑). In the DEPT spectrum at 90° only CH signals are positive (up) and showed *δ*: 130.28 (C-7 ↑), 129.90 (C-9 ↑), 128.87 (aromatic ↑), 128.83 (aromatic ↑), 128.76 (C-6 ↑), 125.89 (C-10 ↑), 118.15 (C-5 ↑), 115.56 (aromatic ↑), 115.39 (aromatic ↑), 37.05 (C-1 ↑). In the DEPT spectrum at 45° (CH, CH_2_ and CH_3_ positive) revealed signals at *δ*: 130.28 (C-7 ↑), 129.90 (C-9 ↑), 128.87 (aromatic ↑), 128.83 (aromatic ↑), 128.76 (C-6 ↑), 125.89 (C-10 ↑), 118.15 (C-5 ↑), 115.56 (aromatic ↑), 115.39 (aromatic ↑), 37.05 (C-1 ↑); ^13^CNMR-APT spectrum CH, CH_3_ [positive (up)], CH_2_, Cq [negative (down)], revealed the following signals at *δ*: 161.79 (aromatic ↓), 159.52 (C-3 ↓), 147.07 (C-4a ↓), 141.69 (aromatic ↓), 132.15 (C-10a ↓), 130.28 (C-7 ↑), 129.90 (C-9 ↑), 128.87 (aromatic ↑), 128.83 (aromatic ↑), 128.76 (C-6 ↑), 128.28 (C-6a ↓), 125.89 (C-10 ↑), 120.21 (C-10b ↓), 118.16 (CN ↓), 118.15 (C-5 ↑), 115.82 (C-8 ↓), 115.56 (aromatic ↑), 115.39 (aromatic ↑), 57.65 (C-2 ↓), 37.05 (C-1 ↑); *m*/*z* (%): 396 (M^+^+2_,_ 10.91), 394 (M^+^, 34.12) with a base peak at 271 (100); Anal. Calcd for C_20_H_12_BrFN_2_O: C, 60.78; H, 3.06; N, 7.09. Found: C, 60.90; H, 3.17; N, 7.20%.

#### 2.2.3. 3-Amino-8-bromo-1-(4-chlorophenyl)-1*H*-benzo[*f*]chromene-2-carbonitrile 4c

Yellow crystals from ethanol; yield 86%; m.p. 260–261 °C (Lit. m. p. 265 °C^28^); IR (KBr) *υ* (cm^−1^): 3449, 3322, 3200 (NH_2_), 2200 (CN); ^1^H NMR *δ*: 8.23–7.19 (m, 9H, aromatic), 7. 10 (bs, 2H, NH_2_), 5.37 (s, 1H, H-1); ^13^C NMR *δ*: 159.55 (C-3), 147.11 (C-4a), 132.14 (C-10a), 130.30 (C-7), 129.96 (C-9), 128.79 (C-6), 128.73 (C-6a), 125.85 (C-10), 120.15 (C-10b), 118.20 (CN), 118.15 (C-5), 115.49 (C-8), 57.29 (C-2), 37.15 (C-1), 144.38, 131.28, 128.97 (aromatic); *m*/*z* (%): 414 (M^+^+4, 3.11), 412 (M^+^+2, 8.65), 410 (M+, 6.31) with a base peak at 299 (100); Anal. Calcd for C_20_H_12_ BrClN_2_O: C, 58.35; H, 2.94; N, 6.80. Found: C, 58.23; H, 2.83; N, 6.69%.

#### 2.2.4. 3-Amino-8-bromo-1-(4-bromophenyl)-1*H*-benzo[*f*]chromene-2-carbonitrile 4d

Colourless crystals from ethanol; yield 88%; m.p. 250–251 °C (Lit. m. p. 250 °C^29^);IR (KBr) *υ* (cm^−1^): 3447, 3321, 3205 (NH_2_), 2201 (CN); ^1^H NMR *δ*: 8.23–7.12 (m, 9H, aromatic), 7.09 (bs, 2H, NH_2_), 5.37 (s, 1H, H-1); ^13^C NMR *δ*: 159.54 (C-3), 147.11 (C-4a), 132.14 (C-10a), 130.31 (C-7), 129.97 (C-9), 128.98 (C-6), 128.73 (C-6a), 125.85 (C-10), 120.13 (C-10b), 118.21 (CN), 118.14 (C-5), 115.42 (C-8), 57.21 (C-2), 37.21 (C-1), 144.80, 131.65, 129.16, 119.79 (aromatic); *m*/*z* (%): 458 (M^+^+4_,_ 31.91), 456 (M^+^+2_,_ 63.81), 454 (M^+^, 32.12) with a base peak at 272 (100); Anal. Calcd for C_20_H_12_Br_2_N_2_O: C, 52.66; H, 2.65; N, 6.14. Found: C, 52.52; H, 2.53; N, 6.02%.

#### 2.2.5. 3-Amino-8-bromo-1-(4-methylphenyl)-1*H*-benzo[*f]*chromene-2-carbonitrile 4e

Colourless needles from ethanol; yield 87%; m.p. 215–216 °C (Lit. m. p. 215 °C^29^); IR (KBr) *υ* (cm^−1^): 3480, 3369, 3217 (NH_2_), 2181 (CN); ^1^H NMR *δ*: 8.22–7.04 (m, 9H, aromatic), 7.00 (bs, 2H, NH_2_, D_2_O exchangeable), 5.26 (s, 1H, H-1), 2.21 (s, 3H, CH_3_); ^13^C NMR *δ*: 159.42 (C-3), 147.04 (C-4a), 132.12 (C-10a), 130.21 (C-7), 129.81 (C-9), 128.86 (C-6a), 128.67 (C-6), 125.99 (C-10), 120.32 (C-10b), 118.11 (C-5), 118.07 (CN), 116.14 (C-8), 57.89 (C-2), 37.51 (C-1), 20.51 (CH_3_), 142.53, 135.78, 129.26, 126.82 (aromatic); ^13^C NMR-DEPT spectrum at 135°CH, CH_3_ [positive (up)], CH_2_ [negative (down)], revealed the following signals at *δ*: 130.21 (C-7 ↑), 129.81 (C-9 ↑), 129.26 (aromatic ↑), 128.67 (C-6 ↑), 126.82 (aromatic ↑), 125.99 (C-10 ↑), 118.11 (C-5 ↑), 37.51 (C-1 ↑), 20.51 (CH_3_ ↑). In the DEPT spectrum at 90° only CH signals are positive (up) and showed *δ*: 130.21 (C-7 ↑), 129.81 (C-9 ↑), 129.26 (aromatic ↑), 128.67 (C-6 ↑), 126.82 (aromatic ↑), 125.99 (C-10 ↑), 118.11 (C-5 ↑), 37.51 (C-1 ↑). In the DEPT spectrum at 45° (CH, CH_2_, and CH_3_ positive) revealed signals at *δ*: 130.21 (C-7 ↑), 129.81 (C-9 ↑), 129.26 (aromatic ↑), 128.67 (C-6 ↑), 126.82 (aromatic ↑), 125.99 (C-10 ↑), 118.11 (C-5 ↑), 37.51 (C-1 ↑), 20.51 (CH_3_ ↑);^13^CNMR-APT spectrum CH, CH_3_ [positive (up)], CH_2_, Cq [negative (down)], revealed the following signals at *δ*: 159.42 (C-3 ↓), 147.04 (C-4a ↓), 142.53 (aromatic ↓), 135.78 (aromatic ↓), 132.12 (C-10a ↓), 130.21 (C-7 ↑), 129.81 (C-9 ↑), 129.26 (aromatic ↑), 128.86 (C-6a ↓), 128.67 (C-6 ↑), 126.82 (aromatic ↑), 125.99 (C-10 ↑), 120.32 (C-10b ↓), 118.11 (C-5 ↑), 118.07 (CN ↓), 116.14 (C-8 ↓), 57.89 (C-2 ↓), 37.51 (C-1↑), 20.51 (CH_3_ ↑); *m*/*z* (%): 392 (M^+^+2_,_ 29.87), 390 (M^+^, 30.02) with a base peak at 271 (100); Anal. Calcd for C_21_H_15_BrN_2_O: C, 64.46; H, 3.86; N, 7.16. Found: C, 64.35; H, 3.76; N, 7.06%.

#### 2.2.6. 3-Amino-8-bromo-1-(4-methoxyphenyl)-1*H*-benzo[*f*]chromene-2-carbonitrile 4f

Colourless needles from ethanol; yield 84%; m.p. 234–235 °C (Lit. m. p. 235 °C^28^); IR (KBr) *υ*(cm^−1^): 3431, 3319, 3199 (NH_2_), 2196 (CN); ^1^H NMR *δ*: 8.21–6.81 (m, 9H, aromatic), 6.99 (bs, 2H, NH_2_, D_2_O exchangeable), 5.26 (s, 1H, H-1), 3.67 (s, 3H, OCH_3_);^13^C NMR *δ*: 159.38 (C-3),146.99 (C-4a), 132.13 (C-10a), 130.21 (C-7), 129.78 (C-9), 128.85 (C-6a), 127.99 (C-6), 126.00 (C-10), 120.38 (C-10b), 118.12 (C-5), 118.06 (CN), 116.29 (C-8), 58.07 (C-2), 54.95 (CH_3_), 37.10 (C-1), 157.88, 137.62, 128.62, 128.28, 114.07 (aromatic); ^13^CNMR-APT spectrum CH, CH_3_ [positive (up)], CH_2_, Cq [negative (down)], revealed the following signals at *δ*: 159.38 (C-3 ↓), 157.88 (aromatic ↓), 146.99 (C-4a ↓), 137.62 (aromatic ↓), 132.13 (C-10a ↓), 130.21 (C-7 ↑), 129.78 (C-9 ↑), 128.86 (C-6a ↓), 128.62 (aromatic ↑), 128.28 (aromatic ↑), 127.99 (C-6 ↑), 126.00 (C-10 ↑), 120.38 (C-10b ↓), 118.12 (C-5 ↑), 118.06 (CN ↓), 116.29 (C-8 ↓), 114.73 (aromatic ↑), 114.07 (aromatic ↑), 58.09 (C-2 ↓), 54.96 (CH_3_ ↑), 37.10 (C-1↑); *m*/*z* (%): 408 (M^+^+2_,_ 9.89), 406 (M^+^, 10.12) with a base peak at 271 (100); Anal. Calcd.for C_21_H_15_BrN_2_O_2_: C, 61.93; H, 3.71; N, 6.88. Found: C, 61.81; H, 3.60; N, 6.77%.

#### 2.2.7. 3-Amino-8-bromo-1-(2,4-dimethoxyphenyl)-1*H*-benzo[*f*]chromene-2-carbonitrile 4g

Colourless crystals from ethanol/benzene; yield 83%; m.p. 220–221 °C; IR (KBr) *υ* (cm^−1^): 3410, 3327, 3202 (NH_2_), 2198 (CN); ^1^H NMR *δ*: 8.19–6.37 (m, 8H, aromatic), 6.90 (bs, 2H, NH_2_, D_2_O exchangeable), 5.47 (s, 1H, H-1), 3.85 (s, 3H, OCH_3_), 3.69 (s, 3H, OCH_3_); ^13^C NMR *δ*: 159.88 (C-3), 147.35 (C-4a), 131.95 (C-10a), 130.20 (C-7), 129.86 (C-9), 128.97 (C-6a), 128.28 (C-6), 125.32 (C-10), 120.34 (C-10b), 117.98 (C-5), 117.93 (CN), 116.40 (C-8), 57.09 (C-2), 55.96 (CH_3_), 55.08 (CH_3_), 31.26 (C-1), 159.25, 156.61, 125.73, 129.21, 105.79, 98.68 (aromatic); ^13^C NMR-DEPT spectrum at 135°CH, CH_3_ [positive (up)], CH_2_ [negative (down)], revealed the following signals at *δ*: 130.20 (C-7 ↑), 129.86 (C-9 ↑), 129.21 (aromatic ↑), 128.28 (C-6 ↑), 125.32 (C-10 ↑), 117.98 (C-5 ↑), 105.79 (aromatic ↑), 98.68 (aromatic ↑), 55.96 (CH_3_ ↑), 55.08 (CH_3_ ↑), 31.26 (C-1 ↑). In the DEPT spectrum at 90° only CH signals are positive (up) and showed *δ*: 130.20 (C-7 ↑), 129.86 (C-9 ↑), 129.21 (aromatic ↑), 128.28 (C-6 ↑), 125.32 (C-10 ↑), 117.98 (C-5 ↑), 105.79 (aromatic ↑), 98.68 (aromatic ↑), 31.26 (C-1 ↑). In the DEPT spectrum at 45° (CH, CH_2_, and CH_3_ positive) revealed signals at *δ*: 130.20 (C-7 ↑), 129.86 (C-9 ↑), 129.21 (aromatic ↑), 128.28 (C-6 ↑), 125.32 (C-10 ↑), 117.98 (C-5 ↑), 105.79 (aromatic ↑), 98.68 (aromatic ↑), 55.96 (CH_3_ ↑), 55.08 (CH_3_ ↑), 31.26 (C-1 ↑); ^13^CNMR-APT spectrum CH, CH_3_ [positive (up)], CH_2_, Cq [negative (down)], revealed the following signals at *δ*: 159.88 (C-3 ↓), 159.25 (aromatic ↓), 156.61 (aromatic ↓), 147.35 (C-4a ↓), 131.95 (C-10a ↓), 130.20 (C-7 ↑), 129.86 (C-9 ↑), 129.21 (aromatic ↑), 128.97 (C-6a ↓), 128.28 (C-6 ↑), 125.73 (aromatic ↓), 125.32 (C-10 ↑), 120.34 (C-10b ↓), 117.98 (C-5 ↑), 117.93 (CN ↓), 116.40 (C-8 ↓), 105.79 (aromatic ↑), 98.68 (aromatic ↑), 57.09 (C-2 ↓), 55.96 (CH_3_ ↑), 55.08 (CH_3_ ↑), 31.26 (C-1 ↑); MS *m*/*z* (%): 438 (M^+^+2_,_ 10.03) 436 (M^+^, 10.33) with a base peak at 63 (100); Anal. Calcd for C_22_H_17_BrN_2_O_3_: C, 60.43; H, 3.92; N, 6.41. Found: C, 60.55; H, 4.03; N, 6.55%.

#### 2.2.8. 3-Amino-8-bromo-1-(3,4-dimethoxyphenyl)-1*H*-benzo[*f*]chromene-2-carbonitrile 4h

Yellow crystals from ethanol/benzene; yield 79%; m.p. 200–201 °C; IR (KBr) *υ* (cm^−1^): 3409, 3327, 3205 (NH_2_), 2197 (CN); ^1^H NMR *δ*: 8.22–6.55 (m, 8H, aromatic), 6.98 (bs, 2H, NH_2_, D_2_O exchangeable), 5.26 (s, 1H, H-1), 3.68 (s, 3H, OCH_3_), 3.67 (s, 3H, OCH_3_); ^13^C NMR *δ*: 159.40 (C-3), 147.00 (C-4a), 132.11 (C-10a), 130.19 (C-7), 129.80 (C-9), 128.97 (C-6a), 128.65 (C-6), 126.09 (C-10), 120.40 (C-10b), 118.95 (C-5), 118.08 (CN), 116.17 (C-8), 57.98 (C-2), 55.48 (CH_3_), 55.43 (CH_3_), 37.43 (C-1), 148.65, 147.51, 138.08, 118.09, 112.16, 111.04 (aromatic); ^13^C NMR-DEPT spectrum at 135°CH, CH_3_ [positive (up)], CH_2_ [negative (down)], revealed the following signals at *δ*: 130.19 (C-7 ↑), 129.80 (C-9 ↑), 128.65 (C-6 ↑), 126.09 (C-10 ↑), 118.95 (C-5 ↑), 118.09 (aromatic ↑), 112.16 (aromatic ↑), 111.04 (aromatic ↑), 55.48 (CH_3_ ↑), 55.43 (CH_3_ ↑), 37.43 (C-1 ↑). In the DEPT spectrum at 90° only CH signals are positive (up) and showed *δ*: 130.19 (C-7 ↑), 129.80 (C-9 ↑), 128.65 (C-6 ↑), 126.09 (C-10 ↑), 118.95 (C-5 ↑), 118.09 (aromatic ↑), 112.16 (aromatic ↑), 111.04 (aromatic ↑), 37.43 (C-1 ↑). In the DEPT spectrum at 45° (CH, CH_2_, and CH_3_ positive) revealed signals at *δ*: 130.19 (C-7 ↑), 129.80 (C-9 ↑), 128.65 (C-6 ↑), 126.09 (C-10 ↑), 118.95 (C-5 ↑), 118.09 (aromatic ↑), 112.16 (aromatic ↑), 111.04 (aromatic ↑), 55.48 (CH_3_ ↑), 55.43 (CH_3_ ↑), 37.43 (C-1 ↑); ^13^CNMR-APT spectrum CH, CH_3_ [positive (up)], CH_2_, Cq[negative (down)], revealed the following signals at *δ*: 159.40 (C-3 ↓), 148.65 (aromatic ↓), 147.51 (aromatic ↓), 147.00 (C-4a ↓), 138.08 (aromatic ↓), 132.11 (C-10a ↓), 130.19 (C-7 ↑), 129.80 (C-9 ↑), 128.97 (C-6a ↓), 128.65 (C-6 ↑), 126.09 (C-10 ↑), 120.40 (C-10b ↓), 118.95 (C-5 ↑), 118.09 (aromatic ↑), 118.08 (CN ↓), 116.17 (C-8 ↓), 112.16 (aromatic ↑), 111.04 (aromatic ↑), 57.98 (C-2 ↓), 55.48 (CH_3_ ↑), 55.43 (CH_3_ ↑), 37.43 (C-1 ↑); MS *m*/*z* (%):438 (M^+^+2_,_ 38.99) 436 (M^+^, 41.38) with a base peak at 79 (100); Anal. Calcd for C_22_H_17_BrN_2_O_3_: C, 60.43; H, 3.92; N, 6.41. Found: C, 60.28; H, 3.77; N, 6.78%.

#### 2.2.9. 3-Amino-1-phenyl-8-methoxy-1*H*-benzo[*f*]chromene-2-carbonitrile 6a

Colourless crystals from ethanol; yield 84%; m.p. 237–238 °C (Lit. m. p. 246 °C^30^); IR (KBr) *υ* (cm^−1^): 3475, 3327, 3197 (NH_2_), 2191 (CN); ^1^H NMR *δ*: 7.85–7.09 (m, 10Hs, aromatic), 6.97 (bs, 2H, NH_2_), 5.27 (s, 1H, H-1), 3.82 (s, 3H, OCH_3_); ^13^C NMR *δ*: 159.80 (C-3), 156.42 (C-8), 145.76 (C-4a), 132.18 (C-6a), 128.65 (C-10a), 126.92 (C-6), 126.53 (C-10), 120.55 (C-10b), 119.03 (C-9), 117.08 (C-7), 115.85 (CN), 107.24 (C-5), 57.77 (C-2), 55.18 (CH_3_), 38.15 (C-1), 145.33, 128.29, 128.17, 125.11 (aromatic); *m*/*z* (%): 328 (M^+^, 16.52) with a base peak at 251 (100); Anal. Calcd for C_21_H_16_N_2_O_2_: C, 76.81; H, 4.91; N, 8.53. Found: C, 76.94; H, 5.03; N, 8.69%.

#### 2.2.10. 3-Amino-1-(4-flourophenyl)-8-methoxy-1*H*-benzo[*f*]chromene-2-carbonitrile 6b

Pale yellow needles from ethanol; yield 81%; m.p. 256–257 °C; IR (KBr) *υ* (cm^−1^): 3467, 3324, 3215 (NH_2_), 2201 (CN); ^1^H NMR *δ*: 7.85–7.07 (m, 9H, aromatic), 6.95 (bs, 2H, NH_2_), 5.32 (s, 1H, H-1), 3.83 (s, 3H, OCH_3_); ^13^C NMR *δ*: 159.78 (C-3), 156.45 (C-8), 145.27 (C-4a), 132.22 (C-6a), 128.73 (C-10a), 126.29 (C-6), 125.06 (C-10), 120.43 (C-10b), 119.07 (C-9), 117.11 (C-7), 115.66 (CN), 107.24 (C-5), 57.64 (C-2), 55.19 (CH_3_), 37.30 (C-1), 161.73, 142.01, 128.79, 115.47, 115.30 (aromatic); *m*/*z* (%): 346 (M^+^, 10.03) with a base peak at 251 (100); Anal. Calcd for C_21_H_15_FN_2_O_2_: C, 72.82; H, 4.37; N, 8.09. Found: C, 72.92; H, 4.50; N, 8.22%.

#### 2.2.11. 3-Amino-1-(4-chlorophenyl)-8-methoxy-1*H*-benzo[*f*]chromene-2-carbonitrile 6c

Pale yellow crystals from ethanol; yield 89%; m.p. 247–248 °C; IR (KBr) *υ* (cm^−1^): 3461, 3323, 3206 (NH_2_), 2201 (CN); ^1^H NMR *δ*: 7.86–7.10 (m, 9H, aromatic), 7.01 (bs, 2H, NH_2_), 5.33 (s, 1H, H-1), 3.83 (s, 3H, OCH_3_); ^13^C NMR *δ*: 159.81 (C-3), 156.47 (C-8), 145.29 (C-4a), 132.21 (C-6a), 128.65 (C-10a), 128.39 (C-6), 125.03 (C-10), 120.37 (C-10b), 119.12 (C-9), 117.11 (C-7), 115.33 (CN), 107.31 (C-5), 57.26 (C-2), 55.20 (CH_3_), 37.39 (C-1), 144.73, 131.11, 128.77, 124.98 (aromatic); MS *m*/*z* (%): 364 (M^+^+2, 3.39), 362 (M^+^, 11.44) with a base peak at 111 (100); Anal. Calcd for C_21_H_15_ClN_2_O_2_: C, 69.52; H, 4.17; N, 7.72. Found: C, 69.61; H, 4.27; N, 7.84%.

#### 2.2.12. 3-Amino-1-(4-bromophenyl)-8-methoxy-1*H*-benzo[*f*]chromene-2-carbonitrile 6d

Pale yellow crystals from ethanol; yield 88%; m.p. 261–262 °C; IR (KBr) *υ* (cm^−1^): 3457, 3321, 3192 (NH_2_), 2201 (CN); ^1^H NMR *δ*: 7.86–7.10 (m, 9H, aromatic), 7.02 (bs, 2H, NH_2_), 5.32 (s, 1H, H-1), 3.83 (s, 3H, OCH_3_); ^13^C NMR (125 MHz, DMSO-d_6_) *δ*: 159.80 (C-3), 156.47 (C-8), 145.29 (C-4a), 132.21 (C-6a), 128.40 (C-10a), 128.29 (C-6), 125.02 (C-10), 120.37 (C-10b), 119.13 (C-9), 117.10 (C-7), 115.25 (CN), 107.32 (C-5), 57.20 (C-2), 55.20 (CH_3_), 37.48 (C-1), 145.14, 131.57, 129.16, 119.61 (aromatic); MS *m*/*z* (%): 408 (M^+^+2, 1.30), 406 (M^+^, 1.46) with a base peak at 75 (100); Anal. Calcd for C_21_H_15_BrN_2_O_2_: C, 61.93; H, 3.71; N, 6.88. Found: C, 61.81; H, 3.59; N, 6.75%.

#### 2.2.13. 3-Amino-8-methoxy-1-(4-methylphenyl)-1*H*-benzo[*f*]chromene-2-carbonitrile 6e

Colourless crystals from ethanol; yield 87%; m.p. 232–233 °C (Lit. m. p. 232 °C^30^); IR (KBr) *υ* (cm^−1^): 3476, 3330, 3208 (NH_2_), 2203 (CN); ^1^H NMR *δ*: 7.84–7.01 (m, 9H, aromatic), 6.92 (bs, 2H, NH_2_), 5.21 (s, 1H, H-1), 3.82 (s, 3H, OCH_3_), 2.21 (s, 3H, CH_3_); ^13^C NMR *δ*: 159.70 (C-3), 156.40 (C-8), 145.26 (C-4a), 132.16 (C-6a), 128.29 (C-10a), 128.08 (C-6), 125.13 (C-10), 120.57 (C-10b), 118.97 (C-9), 117.07 (C-7), 115.96 (CN), 107.21 (C-5), 57.89 (C-2), 55.18 (CH_3_), 37.79 (C-1), 20.51 (CH_3_), 142.85, 135.60, 129.18, 126.82 (aromatic); *m*/*z* (%): 342 (M^+^, 82.13) with a base peak at 241 (100); Anal. Calcd for C_22_H_18_N_2_O_2_: C, 77.17; H, 5.30; N, 8.18. Found: C, 77.01; H, 5.27; N, 8.08%.

#### 2.2.14. 3-Amino-8-methoxy-1-(4-methoxyphenyl)-1*H*-benzo[*f*]chromene-2-carbonitrile 6f

Yellow needles from ethanol; yield 85%; m.p. 222–223 °C (Lit. m. p. 220 °C^30^); IR (KBr) *υ* (cm^−1^): 3447, 3331, 3213 (NH_2_), 2200 (CN); ^1^H NMR *δ*: 7.83–6.81 (m, 9H, aromatic), 6.91 (bs, 2H, NH_2_), 5.21 (s, 1H, H-1), 3.82 (s, 3H, OCH_3_), 3.67 (s, 3H, OCH_3_); ^13^C NMR) *δ*: 159.65 (C-3), 156.39 (C-8), 145.20 (C-4a), 132.17 (C-6a), 127.96 (C-6), 125.15 (C-10), 125.12 (C-10a), 120.62 (C-10b), 118.95 (C-9), 117.09 (C-7), 116.13 (CN), 107.21 (C-5), 58.06 (C-2), 55.18 (CH_3_), 54.95 (CH_3_), 37.35 (C-1), 157.80, 137.95, 128.04, 114.00 (aromatic); ^13^C NMR-DEPT spectrum at 135°CH, CH_3_ [positive (up)], CH_2_ [negative (down)], revealed the following signals at *δ*: 128.04 (aromatic ↑), 127.96 (C-6 ↑), 125.15 (C-10 ↑), 118.95 (C-9 ↑), 117.09 (C-7 ↑), 114.00 (aromatic ↑), 107.21 (C-5 ↑), 55.18 (CH_3_ ↑), 54.95 (CH_3_ ↑), 37.35 (C-1 ↑). In the DEPT spectrum at 90° only CH signals are positive (up) and showed *δ*: 128.04 (aromatic ↑), 127.96 (C-6 ↑), 125.15 (C-10 ↑), 118.95 (C-9 ↑), 117.09 (C-7 ↑), 114.00 (aromatic ↑), 107.21 (C-5 ↑), 37.35 (C-1 ↑). In the DEPT spectrum at 45° (CH, CH_2_, and CH_3_ positive) revealed signals at *δ*: 128.04 (aromatic ↑), 127.96 (C-6 ↑), 125.15 (C-10 ↑), 118.95 (C-9 ↑), 117.09 (C-7 ↑), 114.00 (aromatic ↑), 107.21 (C-5 ↑), 55.18 (CH_3_ ↑), 54.95 (CH_3_ ↑), 37.35 (C-1 ↑); ^13^CNMR-APT spectrum CH, CH_3_ [positive (up)], CH_2_, Cq [negative (down)], revealed the following signals at *δ*: 159.65 (C-3↓), 157.80 (aromatic ↓), 156.39 (C-8 ↓), 145.20 (C-4a ↓), 137.95 (aromatic ↓), 132.17 (C-6a ↓), 128.04 (aromatic ↑), 127.96 (C-6 ↑), 125.15 (C-10 ↑), 125.12 (C-10a ↓), 120.62 (C-10b ↓), 118.95 (C-9 ↑), 117.09 (C-7 ↑), 116.13 (CN ↓), 114.00 (aromatic ↑), 107.21 (C-5 ↑), 58.06 (C-2 ↓), 55.18 (CH_3_ ↑), 54.95 (CH_3_ ↑), 37.35 (C-1 ↑); *m*/*z* (%): 358 (M^+^, 13.92) with a base peak at 251 (100); Anal. Calcd for C_22_H_18_N_2_O_3_: C, 73.73; H, 5.06; N, 7.82. Found: C, 73.61; H, 4.06; N, 7.70%.

#### 2.2.15. 3-Amino-1-(2,4-dimethoxyphenyl)-8-methoxy-1*H*-benzo[*f*]chromene-2-carbonitrile 6g

Colourless crystals from ethanol/benzene; yield 85%; m.p. 210–211 °C; IR (KBr) *υ* (cm^−1^): 3414, 3331, 3203 (NH_2_), 2196 (CN); ^1^H NMR *δ*: 7.80–6.36 (m, 8H, aromatic), 6.81 (bs, 2H, NH_2_), 5.46 (s, 1H, H-1), 3.88 (s, 3H, OCH_3_), 3.82 (s, 3H, OCH_3_), 3.69 (s, 3H, OCH_3_); ^13^C NMR *δ*: 159.10 (C-3), 156.35 (C-8), 145.59 (C-4a), 131.98 (C-6a), 128.29 (C-10a), 127.72 (C-6), 125.25 (C-10b), 124.46 (C-10), 119.03 (C-9), 116.97 (C-7), 116.27 (CN), 107.25 (C-5), 57.20 (C-2), 55.99 (CH_3_), 55.17 (CH_3_), 55.07 (CH_3_), 31.12 (C-1), 160.12, 156.50, 129.12, 120.54, 105.82, 98.59 (aromatic); ^13^C NMR-DEPT spectrum at 135°CH, CH_3_ [positive (up)], CH_2_ [negative (down)], revealed the following signals at *δ*: 129.12 (aromatic ↑), 127.72 (C-6 ↑), 124.46 (C-10 ↑), 119.03 (C-9 ↑), 116.97 (C-7 ↑), 107.25 (C-5 ↑), 105.82 (aromatic ↑), 98.59 (aromatic ↑), 55.99 (CH_3_ ↑), 55.17 (CH_3_ ↑), 55.07 (CH_3_ ↑), 31.12 (C-1 ↑). In the DEPT spectrum at 90° only CH signals are positive (up) and showed *δ*: 129.12 (aromatic ↑), 127.72 (C-6 ↑), 124.46 (C-10 ↑), 119.03 (C-9 ↑), 116.97 (C-7 ↑), 107.25 (C-5 ↑), 105.82 (aromatic ↑), 98.59 (aromatic ↑), 31.12 (C-1 ↑). In the DEPT spectrum at 45° (CH, CH_2_, and CH_3_ positive) revealed signals at *δ*: 129.12 (aromatic ↑), 127.72 (C-6 ↑), 124.46 (C-10 ↑), 119.03 (C-9 ↑), 116.97 (C-7 ↑), 107.25 (C-5 ↑), 105.82 (aromatic ↑), 98.59 (aromatic ↑), 55.99 (CH_3_ ↑), 55.17 (CH_3_ ↑), 55.07 (CH_3_ ↑), 31.12 (C-1 ↑); ^13^CNMR-APT spectrum CH, CH_3_ [positive (up)], CH_2_, Cq [negative (down)], revealed the following signals at *δ*: 160.12 (aromatic ↓), 159.10 (C-3 ↓), 156.50 (aromatic ↓), 156.35 (C-8 ↓), 145.59 (C-4a ↓), 131.98 (C-6a ↓), 129.12 (aromatic ↑), 128.29 (C-10a ↓), 127.72 (C-6 ↑), 125.25 (C-10b ↓), 124.46 (C-10 ↑), 120.54 (aromatic ↓), 119.03 (C-9 ↑), 116.97 (C-7 ↑), 116.27 (CN ↓), 107.25 (C-5 ↑), 105.82 (aromatic ↑), 98.59 (aromatic ↑), 57.20 (C-2 ↓), 55.99 (CH_3_ ↑), 55.17 (CH_3_ ↑), 55.07 (CH_3_ ↑), 31.12 (C-1 ↑); MS *m*/*z* (%): 388 (M^+^, 84.51) with a base peak at 208 (100); Anal. Calcd for C_23_H_20_N_2_O_4_: C, 71.12; H, 5.19; N, 7.21. Found: C, 71.00; H, 5.05; N, 7.09%.

#### 2.2.16. 3-Amino-1-(3,4-dimethoxyphenyl)-8-methoxy-1*H*-benzo[*f*]chromene-2-carbonitrile 6h

Colourless crystals from ethanol/benzene; yield 81%; m.p. 200–201 °C; IR (KBr) *υ* (cm^−1^): 3431, 3337, 3219 (NH_2_), 2185 (CN); ^1^H NMR *δ*: 7.83–6.57 (m, 8H, aromatic), 6.92 (bs, 2H, NH_2_), 5.22 (s, 1H, H-1), 3.82 (s, 3H, OCH_3_), 3.68 (s, 3H, OCH_3_), 3.66 (s, 3H, OCH_3_); ^13^C NMR *δ*: 159.69 (C-3), 156.40 (C-8), 145.24 (C-4a), 132.15 (C-6a), 128.28 (C-10a), 128.07 (C-6), 125.24 (C-10), 120.67 (C-10b), 118.94 (C-9), 117.05 (C-7), 115.99 (CN), 107.19 (C-5), 58.00 (C-2), 55.45 (CH_3_), 55.42 (CH_3_), 55.16 (CH_3_), 37.72 (C-1), 148.63, 147.43, 138.43, 112.11, 111.04 (aromatic); ^13^C NMR-DEPT spectrum at 135°CH, CH_3_ [positive (up)], CH_2_ [negative (down)], revealed the following signals at *δ*: 128.07 (C-6 ↑), 125.24 (C-10 ↑), 118.96 (aromatic ↑), 118.94 (C-9 ↑), 117.05 (C-7 ↑), 112.11 (aromatic ↑), 111.04 (aromatic ↑), 107.19 (C-5 ↑), 55.45 (CH_3_ ↑), 55.42 (CH_3_ ↑), 55.16 (CH_3_ ↑), 37.72 (C-1 ↑). In the DEPT spectrum at 90° only CH signals are positive (up) and showed *δ*: 128.07 (C-6 ↑), 125.24 (C-10 ↑), 118.96 (aromatic ↑), 118.94 (C-9 ↑), 117.05 (C-7 ↑), 112.11 (aromatic ↑), 111.04 (aromatic ↑), 107.19 (C-5 ↑), 37.72 (C-1 ↑). In the DEPT spectrum at 45° (CH, CH_2_, and CH_3_ positive) revealed signals at *δ*: 128.07 (C-6 ↑), 125.24 (C-10 ↑), 118.94 (C-9 ↑), 118.96 (aromatic ↑), 117.05 (C-7 ↑), 112.11 (aromatic ↑), 111.04 (aromatic ↑), 107.19 (C-5 ↑), 55.45 (CH_3_ ↑), 55.42 (CH_3_ ↑), 55.16 (CH_3_ ↑), 37.72 (C-1 ↑); ^13^CNMR-APT spectrum CH, CH_3_ [positive (up)], CH_2_, Cq [negative (down)], revealed the following signals at *δ*: 159.69 (C-3 ↓), 156.40 (C-8 ↓), 148.63 (aromatic ↓), 147.43 (aromatic ↓), 145.24 (C-4a ↓), 138.43 (aromatic ↓), 132.15 (C-6a ↓), 128.28 (C-10a ↓), 128.07 (C-6 ↑), 125.24 (C-10 ↑), 120.67 (C-10b ↓), 118.94 (C-9 ↑), 117.05 (C-7 ↑), 115.99 (CN ↓), 112.11 (aromatic ↑), 111.04 (aromatic ↑), 107.19 (C-5 ↑), 58.00 (C-2 ↓), 55.45 (CH_3_ ↑), 55.42 (CH_3_ ↑), 55.16 (CH_3_ ↑), 37.72 (C-1 ↑); MS *m*/*z* (%): 388 (M^+^, 16.00) with a base peak at 251 (100); Anal. Calcd for C_23_H_20_N_2_O_4_: C, 71.12; H, 5.19; N, 7.21. Found: C, 70.99; H, 5.02; N, 7.07%.

### 2.3. Biological screening

#### 2.3.1. Cell culture and cytotoxicity evaluation using viability assay

**Figure 2.**
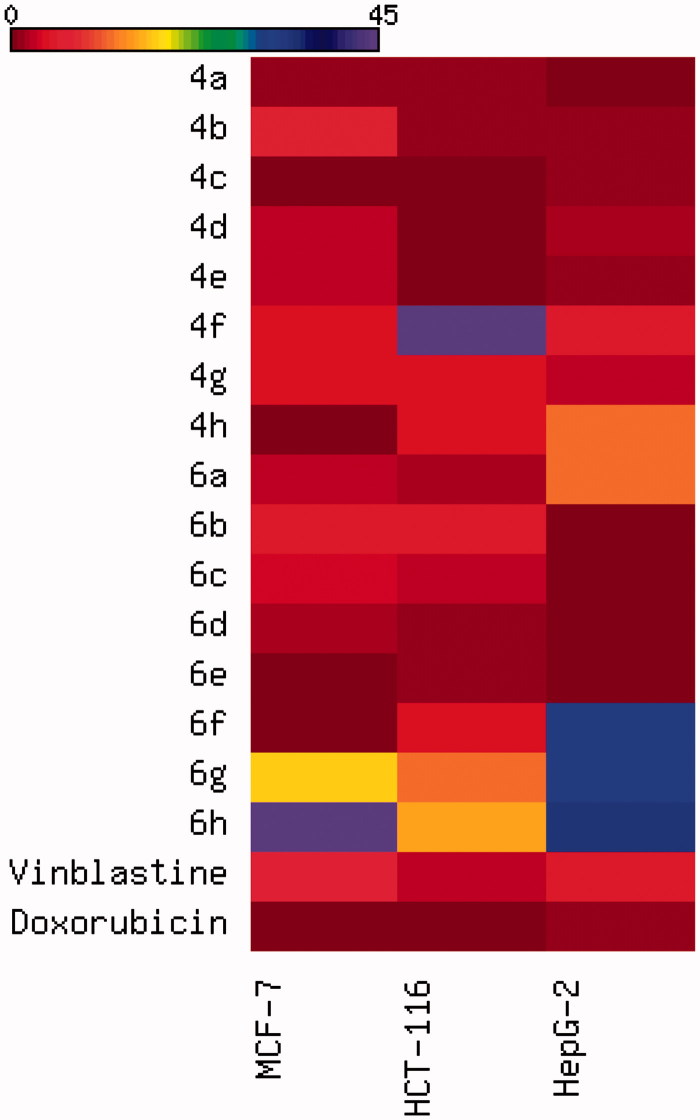
Heatmap of the distribution of IC_50_ values with colour codes of target compounds against the panel of three cancer cell lines.

Compounds **4a–h** and **6a–h** were initially evaluated for *in vitro* antitumour activity against three different human cell lines: MCF-7, HCT-116, and HepG-2, and only the representative active cytotoxic compounds were further evaluated against the normal HFL-1 cell line. The *in vitro* cytotoxicity evaluation was performed at the Regional Center for Mycology & Biotechnology (RCMP), Al-Azhar University under different concentrations (50, 25, 12.5, 6.25, 3.125, 1.56, and 0 µg/mL); Vinblastine and Doxorubicin are used as reference cytotoxic compounds. The measurements of cell growth and the *in vitro* cytotoxicity evaluation were determined using viability assay as described in literature^31–33^ and the result was cited in Table 1 and Figure 2.

**Table 1.** Cytotoxic activity of the target compounds against MCF-7, HCT-116, and HepG-2 cell lines.
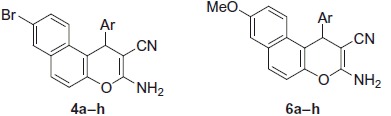

Compound	Ar	IC_50_ (µg/mL)^a^			
		MCF-7	HCT-116	HepG-2	HFL-1
**4a**	C_6_H_5_	0.9 ± 0.29	1.2 ± 0.97	0.7 ± 0.5	
**4b**	4-FC_6_H_4_	5.2 ± 0.03	1.0 ± 0.12	0.8 ± 0.12	
**4c**	4-ClC_6_H_4_	0.5 ± 0.23	0.7 ± 0.11	1.1 ± 0.12	15.2 ± 0.10
**4d**	4-BrC_6_H_4_	2.5 ± 0.14	0.5 ± 0.35	1.8 ± 0.2	
**4e**	4-MeC_6_H_4_	2.5 ± 0.18	0.7 ± 0.25	0.9 ± 0.14	22.4 ± 0.21
**4f**	4-MeOC_6_H_4_	3.8 ± 0.36	44.9 ± 0.14	4.4 ± 0.05	
**4g**	2,4-MeOC_6_H_3_	3.6 ± 0.01	3.7 ± 0.15	2.8 ± 0.17	
**4h**	3,4-MeOC_6_H_3_	0.66 ± 0.04	4.1 ± 0.16	11.0 ± 0.02	12.9 ± 0.13
**6a**	C_6_H_5_	2.7 ± 0.2	2.1 ± 0.23	11.2 ± 0.18	
**6b**	4-FC_6_H_4_	4.4 ± 0.05	4.9 ± 0.01	0.5 ± 0.01	
**6c**	4-ClC_6_H_4_	3.1 ± 0.29	2.5 ± 0.97	0.3 ± 0.08	
**6d**	4-BrC_6_H_4_	2.0 ± 0.25	0.9 ± 0.58	0.7 ± 0.13	35.5 ± 0.22
**6e**	4-MeC_6_H_4_	0.3 ± 0.05	0.8 ± 0.06	0.6 ± 0.21	29.7 ± 0.17
**6f**	4-MeOC_6_H_4_	0.6 ± 0.08	3.8 ± 0.14	29.3 ± 0.12	
**6g**	2,4-MeOC_6_H_3_	16.0 ± 0.92	10.9 ± 0.5	9.0 ± 0.3	
**6h**	3,4-MeOC_6_H_3_	41.1 ± 0.25	13.9 ± 0.3	30.7 ± 0.5	
**Vinblastine**	–	6.1 ± 0.03	2.6 ± 0.08	4.6 ± 0.01	
**Doxorubicin**	–	0.4 ± 0.01	0.5 ± 0.015	0.9 ± 0.04	

a IC_50_ values expressed in µg/mL as the mean values of triplicate wells from at least three experiments and are reported as the mean ± standard error.

#### 2.3.2. Cell invasion assay

The cell invasion assay was performed by Reaction Biology Corporation, One Great Valley Parkway, PA. All compounds were dissolved in DMSO and tested for their ability to cell invasion assay. Compounds **4c**, **6e**, and reference compound bosutinib were purchased from Sellechem (Houston, TX). Corning Bio Coat Fluoro Blok Insert System-96-well plates were purchased from Corning (Corning Incorporated, Tewksbury, MA). Calcein AM labelling reagent was purchased from Molecular Probes (Eugene, Oregon). MDA-MB-231 cell line was purchased from American Type Culture Collection (Manassas, VA). MDA-MB-231 cells were grown in DMEM medium supplemented with 10% foetal bovine serum (FBS), 100 µg/mL of penicillin, and 100 µg/mL of streptomycin. Cultures were maintained at 37 °C in a humidified atmosphere of 5% CO_2_ and 95% air-following incubation; medium was carefully removed from the apical chambers. The insert system was transferred into a second 96-square well plate containing 200 µl/well of 4 µg/mL Calcein AM in HBSS. The plates were incubated for 1 h at 37 °C, 5% CO_2_. Fluorescence of invaded cells was read at wavelengths of 492/535 nm (Ex/Em) from bottom reading using Envision 2104 Multilabel Reader (PerkinElmer, Santa Clara, CA). The IC_50_ curves were plotted and IC_50_ values were calculated using the Graph Pad Prism 4 program for windows (Graph Pad software, San Diego California. www.graphpad.com) based on a sigmoidal dose-response equation.

#### 2.3.3. Caspase 3/7 activity assay

The Caspase-Glo 3/7 activity assay was performed by Reaction Biology Corporation, One Great Valley Parkway, PA. Examined compounds **4c**, **4e**, **4h**, **6d**, and **6e** were dissolved in DMSO as 20 mM stock and tested for their ability to Caspase-Glo 3/7 activity assay. The reference compound staurosporine was purchased from Sigma-Aldrich (Saint Louis, MO) and dissolved in DMSO in 10 mM stock. Caspase-Glo 3/7 activity assay reagent was purchased from Promega (Madison, WI). MDA-MB-231 cell line was provided by ATCC. MDA-MB-231 cell line culture conditions: DMEM with 10% FBS. 100 µg/mL penicillin and 100 µg/mL streptomycin were added to the culture media. Cultures were maintained at 37 °C in a humidified atmosphere of 5% CO_2_ and 95% air.

#### 2.3.4. *c-Src* kinase assay

The effect of the synthesised compounds on the activity of *c-Src* kinase was determined by HTScan Src Kinase Assay Kit, catalogue number 7776 from Cell Signaling Technology (Danvers, MA); according to the manufacturer’s protocol. Streptavidin-coated plates were purchased from Pierce (Rockford, IL). In brief, the kinase reaction was started with the incubation of the 12.5 µL of the reaction cocktail (0.5 ng/µL of GST-Src kinase in 1.25 mM DTT) with 12.5 µL of prediluted compounds (dissolved in 1% DMSO) for 5 min at room temperature. ATP/substrate (25 µL, 20 µM/1.5 uM) cocktail was added to the mixture. The biotinylated substrate (catalogue number 1366) contains the residues surrounding tyrosine 160 (Tyr160) of signal transduction protein and has a sequence of EGIYDVP. The reaction mixture was incubated for 30 min at room temperature. The kinase reaction was stopped with the addition of 50 µL of 50 mM EDTA (pH 8.0). The reaction solution (25 µL) was transferred into 96-well streptavidin plates (Pierce, part number 15125), diluted with 75 µL double distilled water, and incubated at room temperature for 60 min. At the end of the incubation, the wells were washed three times with 200 µL of 0.05% Tween-20 in PBS buffer (PBS/T). After that to each well was added 100 µL of phosphotyrosine antibody (P-Tyr-100) (1:1000 dilution in PBS/T with 1% BSA) and the wells were incubated for another 60 min. After washing three times with 0.05% Tween-20 in PBS/T, the wells were incubated with 100 µL secondary anti-mouse IgG antibody, which was HRP-conjugated (1:500 dilution in PBS/T with 1% BSA) for next 30 min at room temperature. The wells were washed five times with 0.05% Tween-20 in PBS and then were incubated with 100 µL of 3,3′,5,5′-tetramethylbenzidine dihydrochloride substrate for 5 min. The reaction was stopped by adding 100 µL/well of stop solution to each well and mixed well and read the absorbance at 450 nm using a microplate reader (Molecular devices, spectra Max M2). IC_50_ values of the compounds were calculated using ORIGIN 6.0 (Origin Lab Corporation, Northampton, MA) software. IC_50_ is the concentration of the compound that inhibited enzyme activity by 50%. All the experiments were carried out in triplicate^24^.

### 2.4. Molecular docking

Molecular docking simulation was carried out with the program AutoDock 4.0.1^34^ (GNU General Public License). The three-dimensional (3D) structure of *c-Src* kinase was obtained from Protein Data Bank (PDB entry no. 2src). The ligand and solvent molecules were removed from the crystal structure to obtain the docking grid and the active site was defined using AutoGrid. The grid box was centred on the centre of the ligand from the corresponding crystal structure complexes. The Lamarckian genetic algorithm issued for docking with the following settings: a maximum number of 2,500,000 energy evaluations, an initial population of 50 randomly placed individuals, a maximum number of 37,000 generations, a mutation rate of 0.02, across over rate of 0.80, and an elitism value (number of top individuals that automatically survive) of 1. The ligand was fully optimised inside the binding site during the docking simulations. Finally, the conformation with the lowest predicted binding free energy of the most occurring binding modes in *c-Src* active pocket was selected. Hydrogen atoms were added to the structure with the Molecular Operating Environment (MOE 2012)^35^ and atomic partial charges were calculated using Auto-Dock Tools. Selected active compounds were docked into the active site of microorganisms' targets to predict compound binding modes. For flexible docking, Auto-Dock standard parameter settings were applied. High-scoring binding poses were selected on the basis of visual inspection.

### 2.5. Statistical analysis

All statistical calculations were done using computer programs, Microsoft excel version 10, SPSS version 20.00 (SPSS Inc., IBM in 2009, Chicago, IL) statistical program at 0.05, 0.01, and 0.001 level of probability^36^. Comparisons of inhibiting tumour growth between treatments groups or the control were done using Student's *t* test, one-way ANOVA, and *post hoc* the least significant difference tests measurement.

## 3. Results and discussion

### 3.1. Chemistry

**Scheme 1.**
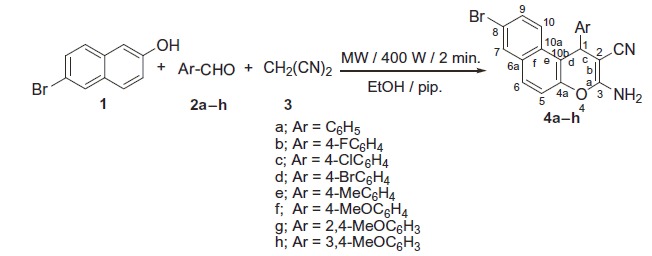
Synthesis of 3-amino-1-aryl-6-bromo-1*H*-benzo[*f*]chromene-3-carbonitriles (**4a**–**h**).

**Scheme 2.**
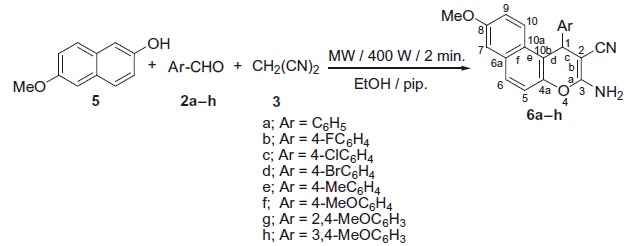
Synthesis of 3-amino-1-aryl-8-methoxy-1*H*-benzo[*f*]chromene-3-carbonitriles (**6a**–**h**).

Benzochromene molecules have been synthesised using the synthetic strategy illustrated in Schemes 1 and 2. In a one-pot three component heterocyclocondensation process, 3-amino-1-aryl-8-bromo-1*H*-benzo[*f*]chromene-2-carbonitrile **4a–h** were obtained via the reaction of 6-bromo-2-naphthol **1** with a mixture of appropriate aromatic aldehydes **2a–h** and malononitrile **3** in ethanol/piperidine solution under microwave irradiation conditions for 2 min at 140 °C.

The IR spectra of compounds **4a–h** revealed the presence of three peaks at υ 3477–3409, 3369–3321, 3217–3199 cm^−1^, and one peak at υ 2204–2181 cm^−1^ which are assigned to the amino and cyano groups, respectively. In the meantime, the ^1^H NMR spectra of compounds **4a–h** displayed a singlet signal of the 1*H* protons in the range of *δ* 5.47–5.26 ppm and a broad signal of the amino group in the range of *δ* 7.10–6.90 ppm. In addition, compounds **4a** and **4e–h** revealed the presence of D_2_O as a replacement to the NH_2_ proton at *δ* 7.04–6.90 ppm. Furthermore, the ^1^H NMR spectra of compounds **4e** and **4f–h** exhibited signals of the methyl and methoxy groups at *δ* 2.21 and 3.85–3.67 ppm, respectively. On the other hand, the ^13^C NMR spectra of compounds **4a–h** displayed one signal of the chiral carbon resonating at *δ* 37.89–31.26 ppm, and compounds **4f–h** exhibited signals of the methyl and methoxy groups at *δ* 20.51 and 55.96–54.96 ppm, respectively. Also, the ^13^C NMR-DEPT spectra at 45°, 90°, and 135 °C as well as the ^13^C NMR-APT spectra of compounds **4b**, **4e–h** provided additional evidence to support the proposed structures (See supplemental data).

The same methodology has been employed to prepare the second series of benzochromene molecules; compounds **6a–h**, Scheme 2 illustrated the interaction of 6-methoxy-2-naphthol **5** with a mixture of appropriate aromatic aldehydes, **2a–h**, and malononitrile **3** to afford the corresponding 3-amino-1-aryl-8-methoxy-1*H*-benzo[*f*]chromene-2-carbonitriles, **6a–h**. The maximum power of the microwave irradiation was optimised by repeating the reaction at different watt powers and periods of time. The optimum condition was obtained by using the microwave irradiations at 400 W and reaction time 2 min, which gave the highest yield of the desired compounds. It is also important to note that the 1-position of compounds **4a–h** and **6a–h** is a chiral centre and all the reactions were controlled using the TLC technique.

The IR spectra of the target compounds **6a–h** confirmed the presence of the characteristics of the NH_2_ absorption bands around *υ* 3475–3414, 3337–3321, 3219–3192 cm^−1^ and the CN absorption bands around υ 2203–2185 cm^−1^. The ^1^H NMR spectra of **6a–h** showed singlet signals of 1*H*, NH_2_, and methyl or methoxy groups protons at *δ* 5.46–5.21, 7.02–6.81 and 2.21, and 3.88–3.66 ppm, respectively. Furthermore, the ^13^C NMR spectra of compounds **6a–h** showed signals resonating in the range of *δ* 38.15–31.12 ppm which are attributed to the chiral carbon, while the carbons of the methyl and methoxy groups appeared in the range of *δ* 20.51 and 55.45–54.95 ppm, respectively. Besides, the ^13^C NMR-DEPT spectra at 45°, 90°, and 135 °C as well as the ^13^C NMR-APT spectra of compounds **6f–h** provided additional evidence in supporting the proposed structures (See supplemental data).

### 3.2. Antitumour assay

The *in vitro* cytotoxic evaluation of the prepared compounds **4a–h** and **6a–h** was performed against MCF-7 (breast cancer), HCT-116 (human colon cancer), HepG-2 (liver cancer), and HFL-1(normal fibroblast lung) using the 3-(4,5-dimethylthiazol-2-yl)-2,5-diphenyl tetrazolium bromide (MTT) colorimetric assay as described in literature^31–33^. Doxorubicin, one of the most effective anticancer agents, and the cancer drug Vinblastine was included in the experiment as positive controls. The results were expressed as growth inhibitory concentration (IC_50_) values, which represent the compounds’ concentrations required to produce a 50% inhibition of the cell growth after 24 h of incubation, compared to the untreated controls as shown in Table 1 and Figure 2.

According to the obtained results, it was obvious that most of the synthesised compounds displayed excellent anti-proliferative activity against the tested cancer cell lines. Investigations of the cytotoxic activity against the three cancer cell lines, MCF-7, HCT-116, and HepG-2, indicated that the HepG-2 was the most sensitive cell line to the influence of the target compounds. In the meantime, the treatment of the MCF-7 cancer cell lines was very successful using the first series compounds, **4a–h** which displayed IC_50_ values in the range of 0.5–5.2 µg/mL. The same effect was obtained using compounds **6a–f** of the second series which exhibited IC_50_ values between 0.3 and 4.4 µg/mL, Table 1. These values were found to be more potent and efficacious against MCF-7 cancer cell lines than Vinblastine (IC_50_ = 6.1 µg/mL) while compound **6e** (IC_50_ = 0.3 µg/mL) was found to be the most potent derivative against MCF-7 as compared to Doxorubicin (IC_50_ = 0.4 µg/mL); compounds **4c** and **6f** (IC_50_= 0.5 and 0.6 µg/mL) are almost equipotent as Doxorubicin and emerged as the most potent counterpart against MCF-7 in this study. Concerning the cytotoxic activity against HCT-116, compounds **4a–e** of the first series with IC_50_ values of 0.5–1.2 µg/mL and compounds **6a**, **6c–e** with IC_50_ in the range of 0.8–2.5 µg/mL displayed significant growth inhibitory activity in comparison to Vinblastine (IC_50_ = 2.6 µg/mL). In particular, compound **4d** (IC_50_ = 0.5 µg/mL) was equipotent as Doxorubicin (IC_50_ = 0.5 µg/mL). The same effect has also been achieved using compounds **4c**, **4e**, **6d**, and **6e** with IC_50_ between 0.7 and 0.9 µg/mL. On the other hand, the cytotoxic evaluation against HepG-2 cell lines revealed that the second series elicited considerable anti-proliferative activity than the first series. For instance, seven members of the first series **4a–g** with IC_50_ values in the range of 0.5–4.4 µg/mL and four members of the second series **6b–e** with IC_50_ values between 0.3 and 0.7 µg/mL were more potent and efficient than Vinblastine (IC_50_ = 4.6 µg/mL) while compounds **6b–e**, **4a**, and **4b** with IC_50_ ranging from 0.3 to 0.8 µg/mL displayed significant growth inhibitory activity in comparison to Doxorubicin (IC_50_ = 0.9 µg/mL). Compound **4e** (IC_50_ = 0.9 µg/mL) was equipotent as Doxorubicin. In addition, the potent compounds **4c**, **4e**, **4h**, **6d**, and **6e** were evaluated for their cytotoxic effect against normal fibroblast cell line. The obtained data revealed that all compounds displayed weak cytotoxicity (IC_50_ = 13–35 µg/mL) compared to their anticancer effect, proving the selectivity behaviour. Finally, the remaining compounds exhibited equipotent or moderate to fair cytotoxic activities against the three tumour cell types compared to Vinblastine and Doxorubicin, and the highly active ones could be considered as selective anticancer agents.

### 3.3. Cell invasion assay

**Figure 3.**
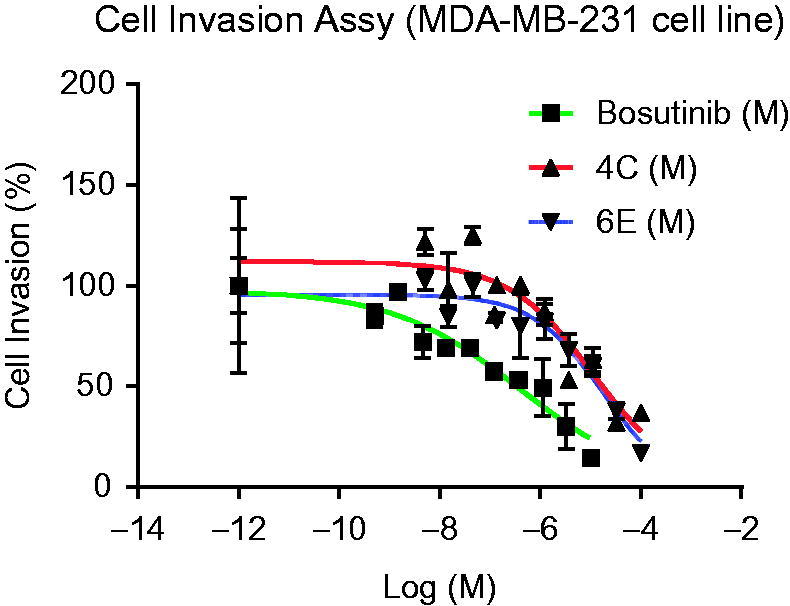
The effect of selected benzochromene derivatives on the invasion of MDA-MB-231 cell line.

**Figure 4.**
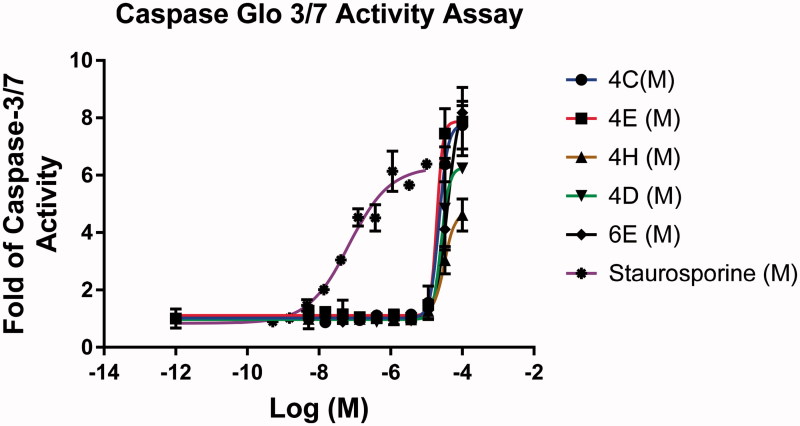
Apoptosis induction by caspase-3/7 activity assay detection in MDA-MB-231 cell line after compound treatment against reference drug.

Tumour invasion is a common feature of triple-negative breast cancers. Triple-negative breast cancers (12–24% of breast cancers)^37^^,^^38^ which are characterised by the absence of ER, progesterone receptors, and HER-2 expression, resulted in high morbidity and mortality owing to their rapid growth rate, invasive potential, and frequently acquired treatment resistance. The invasion process has become an important prerequisite for cancer progression and metastasis. Therefore, therapeutic strategies for preventing or suppressing cancer invasion and metastasis can significantly improve the survival of triple-negative breast cancer patients. The two most active compounds **4c** and **6e** were examined for their ability to inhibit the invasiveness of the highly sensitive and invasive breast cancer cell line, MDA-MB-231. A bosutinib drug was used as a control ant-invasive agent. As shown in Figure 3, **4c** and **6e** derivatives have markedly suppressed the invasion of the MDA-MB-231 cell up to the highest percentage. As shown in Table 2, they showed comparable potent ant-invasion activity with IC_50_ of 10.5 and 16.5 µM, respectively. The obtained data might confirm that the cytotoxicity of novel molecules led to the death of the cancer cells and stopped the metastatic processes.

**Table 2.** IC_50_ values for two most potent ant-invasion compounds in MDA-MB-231 breast cancer cells.

Compound ID	IC_50_ (M)
**4c**	1.08 × 10^−5^
**6e**	1.65 × 10^−5^

### 3.4. Caspase 3/7 activity assay

Caspases are ATP-dependent enzymes that promote apoptosis. Caspases are vital biochemical features of apoptotic signalling. The apoptotic response of MDA-MB-231 cells to potent anti-proliferative compounds was examined via quantifying the activity of caspases 3/7. To determine whether the cell growth inhibition in response to the benzochromene derivatives was mediated by apoptosis and was caspase dependent, MDA-MB-231 cells were treated with 10 different concentrations of the target compounds for 24 h. As shown in Figure 4, caspase-3/7 activity was activated in a dose-dependent manner. The target benzochromene compounds significantly increased caspase-3/7 activity to a 10-fold at a certain concentration and time point, Table 3 and Figure 4.

**Table 3.** Caspase-Glo 3/7 activity assay.

Compound ID	MDA-MB-231 cell line, IC_50_ (M)
**4c**	2.26 × 10^−5^
**4e**	1.95 × 10^−5^
**4h**	3.15 × 10^−5^
**6d**	2.56 × 10^−5^
**6e**	3.89 × 10^−5^
**Staurosporine**	6.85 × 10^−8^

### 3.5. *c-Src* kinase assay

As a biological step for the discovery of their anti-proliferative mechanism, further *c-Src* kinase inhibitory activity of the potent compounds **4c**, **4e**, **4h**, **6d**, and **6e** were performed, Table 4. It is clearly presented that their inhibitory activity is potent and the IC_50_ values ranged between 9 and 329 nM, which mostly exhibited higher activity than the reference drug. For instance, compound **4e**, with the 4-methylphenyl substitution, exhibited higher *c-Src* kinase inhibitory activity (IC_50_ = 9 nM) than the rest. Similarly, compound **6e**, with the 4-methylphenyl substitution, was the most effective compound in the 8-methoxy series with an IC_50_ value of 9 nM. Additionally, 3,4-dimethoxyphenyl and 4-bromophenyl substituted compounds **4 h** and **6d** were among the moderately effective inhibitors in both the 8-bromo and the 8-methoxy series of compounds with IC_50_ ranging from 214 to 329 nM. Lastly, the substitution of the aryl terminal moiety with the electron-donating group (methyl group) in compounds **4e** and **6e** led to increase in inhibitory activity, suggesting that substitution at position 4 of the phenyl ring is detrimental on the *c-Src* kinase inhibition.

**Table 4.** Inhibition of *c-Src* kinase activity data as IC_50_ values (nM) of selected 1*H*-benzo[*f*]-chromene derivatives.

Compound ID	IC_50_ (nM)
**4c**	89
**4e**	9
**4h**	214
**6d**	329
**6e**	9
Staurosporine	112

### 3.6. SAR studies

The preliminary SAR study has focussed on the effect of the presence of the phenyl ring on the antitumour activities of the synthesised compounds. The influence of replacing the H atom at the 4-position in the phenyl ring of the 1*H*-benzo[*f*]chromene moiety with the electron-withdrawing (F, Cl, Br) or electron-donating (Me, MeO) groups, and the presence of a bromine atom (electron-withdrawing) or methoxy group (electron-donating) at the 8-position has also been discussed. In a comparison of the cytotoxic activities of the two series **4a–h** and **6a–h** against MCF-7 breast cancer, we found that the highest growth inhibitory effect of the first series **4a–h** was associated with the presence of 4-chlorophenyl **4c**, 3,4-dimethoxyphenyl **4h**, unsubstituted phenyl **4a**, 4-bromophenyl **4d**, 4-methylphenyl **4e**, 2,4-dimethoxyphenyl **4g**, 4-methoxyphenyl **4f**, and 4-fluorophenyl **4b**, respectively. As previously mentioned, these molecules showed IC_50_ values in the range of 0.5–5.2 µg/mL against MCF-7, which are considered excellent activities relative to Vinblastine (IC_50_ = 6.1 µg/mL), Table 5. On the other hand, the inhibitory effect of the second series **6a–h** was more potent and efficacious than Vinblastine (IC_50_ = 6.1 µg/mL). The IC_50_ values of these molecules were between 0.3 and 4.4 µg/mL against MCF-7, and their effect was as follows: 4-methylphenyl **6e**, 4-methoxyphenyl **6f**, 4-bromophenyl **6d**, un-substituted phenyl **6a**, 4-chlorophenyl **6c**, and 4-fluorophenyl **6b**, respectively, Table 5. According to the obtained data and regarding the first series, it was found that the replacement of the H atom by the chlorine atom (electron-withdrawing group) at the 4-position in the phenyl ring of the 1*H*-benzo[*f*]chromene moiety was significant for its activity against the MCF-7 compared to the dimethoxy (electron-donating group) at 3,4-positions on the same ring with bromine atom at the 8-position. However, the introduction of a methyl or methoxy group at 4-position (electron-donating group) on the phenyl ring, at position 1 of the second series is more beneficial than an electron-withdrawing substituent like Br, Cl, or F atoms, taking into consideration that the second series **6a–h** (more electron-donating group) exhibited significant growth inhibitory activity against MCF-7 which is more effective than the first series **4a–h** (more electron-withdrawing group). In general, the SAR image of the novel potent derivatives indicates that the presence of different pharmacophoric functionalities with small hydrophobic moieties as halogens or slightly hydrophobic fragments as methoxy play an important role in the cytotoxic activity.

**Table 5.** Positive and negative controls and effectiveness of the test compounds against MCF-7.

Control/Cpd.	IC_50_ (μg/mL)	Cell line	*F* ratio	*p* Value
**Vinblastine**	6.1 ± 0.03	MCF-7	221.234	0.000 (HS)
**4c**	0.5 ± 0.23			
**4h**	0.66 ± 0.04			
**4a**	0.9 ± 0.29			
**4d**	2.5 ± 0.14			
**4e**	2.5 ± 0.18			
**4g**	3.6 ± 0.01			
**4f**	3.8 ± 0.36			
**4b**	5.2 ± 0.03			
**6e**	0.3 ± 0.05			
**6f**	0.6 ± 0.08			
**6d**	2.0 ± 0.25			
**6a**	2.7 ± 0.2			
**6c**	3.1 ± 0.29			
**6b**	4.4 ± 0.05			

Positive control (active compounds) and negative control (standard drugs).

All statistical calculations were done as the mean values of triplicate.

HS: Highest significantly at *p* values <0.05.

Investigations of the cytotoxic activity against HCT-116, human colon cancer, indicated that compounds **4a–e** of the first series with IC_50_ = 0.5–1.2 µg/mL and compounds **6a**,**c–e** of the second series with IC_50_ = 0.8–2.5 µg/mL displayed a good inhibitory activity compared to Vinblastine (IC_50_ = 2.6 µg/mL), Table 6. These results are intimate that the grafting of a lipophilic electron-withdrawing substituent (halogen atoms) is more beneficial than an electron-donating substituent (methyl or unsubstituted phenyl group) for the activity of the bromine atom or the methoxy group at the 8-position. Moreover, the first series **4a–h** (electron-withdrawing group) revealed significant growth inhibitory activity against HCT-116 in comparison to the second series **6a–h** (electron-donating group).

**Table 6.** Positive and negative controls and effectiveness of the test compounds against HCT-116.

Control/Cpd.	IC_50_ (μg/mL)	Cell line	*F* ratio	*p* Value
**Vinblastine**	2.6 ± 0.08	HCT-116	57.243	0.000 (HS)
**4d**	0.5 ± 0.35			
**4c**	0.7 ± 0.11			
**4e**	0.7 ± 0.25			
**4b**	1.0 ± 0.12			
**4a**	1.2 ± 0.97			
**6e**	0.8 ± 0.06			
**6d**	0.9 ± 0.58			
**6a**	2.1 ± 0.23			
**6c**	2.5 ± 0.97			

Positive control (active compounds) and negative control (standard drugs).

All statistical calculations were done as the mean values of triplicate.

HS: Highest significantly at *p* value <0.05.

Regarding the activity against the HepG-2 cell line (liver cancer), the order of the antitumour activity of the first series was found to be **4a **>** 4b** >** 4e **>** 4c** >** 4d **>** 4g **>** 4f** (IC_50_ = 0.7–4.4 µg/mL) and for the second series **6c **>** 6b **>** 6e **>** 6d**, with IC_50_ = 0.3–0.7 µg/mL, Table 7. These results suggested that the un-substituted phenyl (electron-donating group) of the first series is preferred as an antitumor agent in comparison to the substituted phenyl (electron-withdrawing group) with bromine atom at the 8-position; however, the electron-withdrawing substituent is preferred compared to electron-donating substituent for the second series with methoxy group at the 8-position, keeping in mind that the second series, **6a–h** (electron-withdrawing and electron-donating groups), is more effective towards the HepG-2 than the first series, **4a–h** (electron-donating and electron-withdrawing groups).

**Table 7.** Positive and negative controls and effectiveness of the test compounds against HepG-2.

Control/Cpd.	IC_50_ (μg/mL)	Cell line	*F* ratio	*p* Value
**Vinblastine**	4.6 ± 0.18	HepG-2	270.518	0.000 (HS)
**4a**	0.7 ± 0.06			
**4b**	0.8 ± 0.06			
**4e**	0.9 ± 0.06			
**4c**	1.1 ± 0.08			
**4d**	1.8 ± 0.12			
**4g**	2.8 ± 0.11			
**4f**	4.4 ± 0.09			
**6c**	0.3 ± 0.02			
**6b**	0.5 ± 0.03			
**6e**	0.6 ± 0.01			
**6d**	0.7 ± 0.04			
**Doxorubicin**	0.9 ± 0.05	HepG-2	8.210	0.000 (HS)
**4a**	0.7 ± 0.08			
**4b**	0.8 ± 0.03			
**4e**	0.9 ± 0.06			
**6c**	0.3 ± 0.08			
**6b**	0.5 ± 0.06			
**6e**	0.6 ± 0.04			
**6d**	0.7 ± 0.02			

Positive control (active compounds) and negative control (standard drugs).

All statistical calculations were done as the mean values of triplicate.

HS: Highest significantly at *p* value <0.05.

On the other hand, the rest of the compounds showed almost equipotent or moderate to fair cytotoxic activities against the three tumour cell lines compared to Vinblastine. While compound **6e** (IC_50_ = 0.3 µg/mL) was found to be the most potent derivative against MCF-7 compared to Doxorubicin (IC_50_ = 0.4 µg/mL) as shown in Table 8. Compound **4c** (IC_50_ = 0.5 µg/mL) was almost as equipotent as Doxorubicin, and compound **4d** (IC_50_ = 0.5 µg/mL) was equipotent as Doxorubicin against HCT-116. Furthermore, compounds **6c**, **6b**, **6e**, **4a**, and **4b** with IC_50_ = 0.3–0.8 µg/mL displayed significant growth inhibitory activity against HepG-2 in comparison to Doxorubicin (IC_50_ = 0.9 µg/mL) as shown in Table 7, and compound **4e** (IC_50_ = 0.9 µg/mL) was equipotent as Doxorubicin indicating that the electron-withdrawing substituent is preferred for antitumour activity compared to the electron-donating substituent.

**Table 8.** Positive and negative controls and effectiveness of the test compound against MCF-7.

Control/Cpd.	IC_50_ (μg/mL)	Cell line	*T* test	*p* Value
**Doxorubicin**	0.4 ± 0.01	MCF-7	1.225	0.288 (NS)
**6e**	0.3 ± 0.05			

Positive control (active compounds) and negative control (standard drugs).

All statistical calculations were done as the mean values of triplicate.

NS: not significant at *p* values >0.05.

Finally, we can deduce that the substitution pattern of the phenyl group at the 1-position of the 1*H*-benzo[*f*]chromene moiety played a vital role in its antitumour activity. The incorporation of halogen atoms (electron-withdrawing group) has also greatly enhanced the activity of the desired compounds in comparison to the electron-donating groups as methyl or methoxy groups with bromine atom or methoxy group at the 8-position. Overall, the growth inhibitory activity of the second series exceeded the first one.

### 3.7. Molecular docking

**Figure 5.**
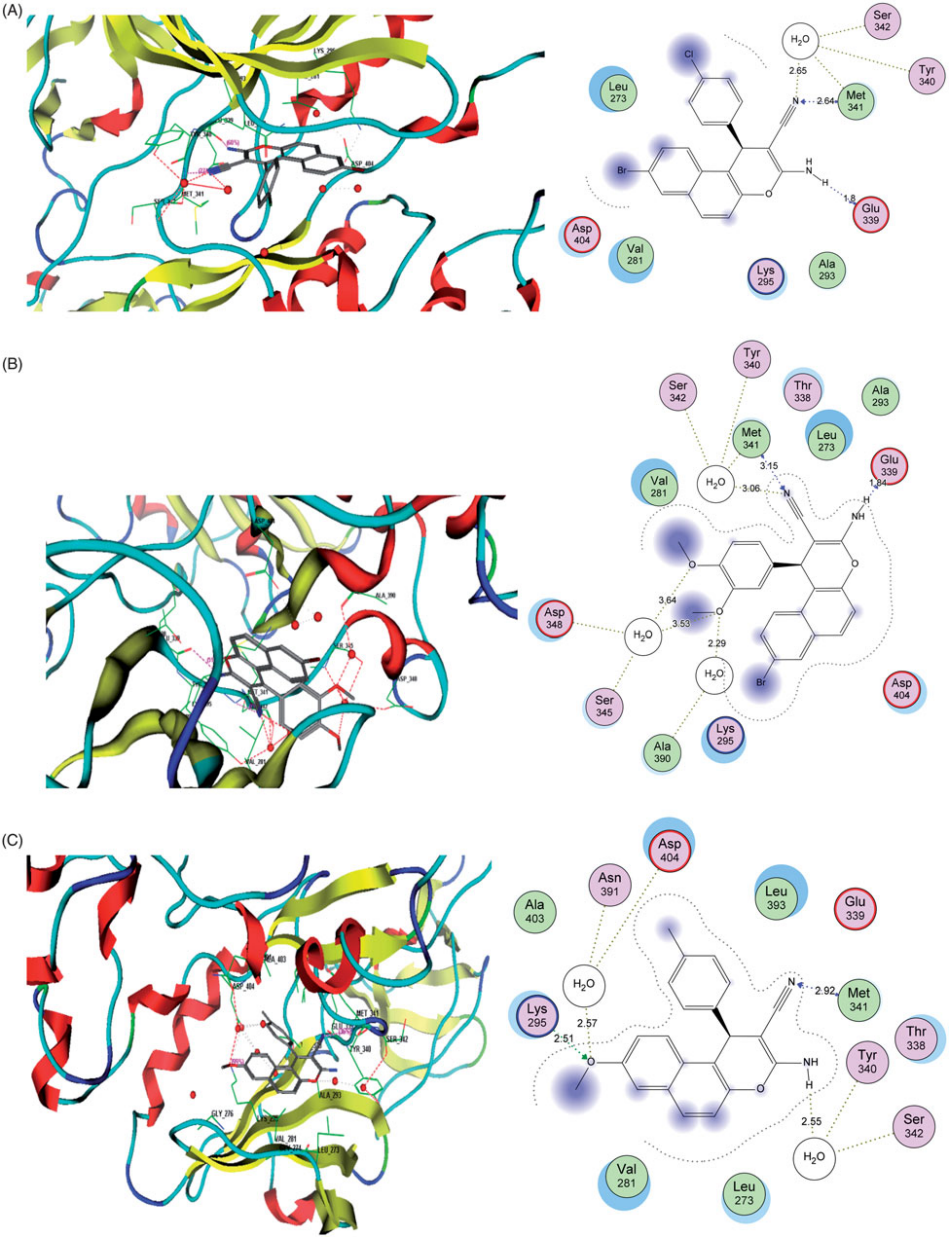
Binding data of target compounds. (A) 3D and 2D depiction of the docked conformation of the selected active compounds (A) 4c, (B) 4h, and (C) 6e in the active site of enzyme revealing the essential residues and types of interactions.

*c-Src* kinase is a non-receptor tyrosine kinase which plays essential roles in controlling a myriad of fundamental cellular processes, including cell proliferation, adhesion, migration, and invasion^39^^,^^40^. *c-Src* kinase is overexpressed and/or is aberrantly activated in a variety of human tumours, including breast, colon, pancreatic, bladder, ovarian, and brain carcinomas^41^^,^^42^. Notably, increased *c-Src* activity is believed to correlate strongly with the metastatic potential and the poor prognosis in breast cancers^41^^,^^43^^,^^44^. Thus, *c-Src* kinase has been recognised as an attractive molecular target for cancer therapy, and the development of *c-Src* inhibitors for cancer treatment is an increasing interest. In this report, we docked the potent active anti-proliferative derivatives **4c**, **4e**, **4h**, **6d**, and **6e** in the ATP-binding pocket of the target enzyme. The placement was performed based upon the position of the co-crystallised ligand ANP. A root mean square deviation (rmsd) of 0.29 and binding energies data were resulted, suggesting a high similarity of interaction to the referenced ligand. Investigation of the docking results reported different binding modes of the target compounds. These compounds consistently showed a stable hydrogen-bonding interaction with the NH of Met 341 residue as a donor with all nitrile fragments of the structures. However, in some cases, the interaction was supported with extra hydrogen bonds with the carbonyl group of Glu 339 as an acceptor through the NH_2_ fragment. Moreover, Asp 404 and Lys 295 exhibited additional hydrogen bonds through the OCH_3_ substituents. In addition, a hydrophobic interaction was found between the naphthalene substructure of compounds within the hydrophobic pockets in the active site, Figure 5 and Table 9. Finally, the hydrogen-bonding donor-acceptor motif which was proposed as a crucial key for the kinase inhibitory activity was accomplished by the active compounds fragments with the corresponding Met 341 or Glu 399 residues^45^. This behaviour towards *c-Src* kinase might confirm the cytotoxic effect of the studied compounds is mediated through the inhibition of such promising target.

**Table 9.** Description of the docking data of selected target compounds.

Cop.	Fragment	Target residues (distance, Å)	Interaction	Binding energy (dG)
**4c**	CN	Met341 (2.64), H_2_O-Ser342 (2.65)	H-bonding	**−16.5**
	NH_2_	Glu339 (1.80)	H-bonding	
	Cl	Leu273	Hydrophobic	
	Br-Naphthalene	Val281, Asp404, Leu273, Lys295	Hydrophobic	
**4h**	CN	Met341 (3.15)	H-bonding	**−15.4**
	NH_2_	Glu339 (1.84)	H-bonding	
	MeO	Lys295, H_2_O-Asp346, H_2_O-Ser345, H_2_O-Ala390 (3.64) (3.53) (2.29)	H-bonding	
	Br-Naphthalene	Asp404, Lys295	Hydrophobic	
**6e**	CN	Met341 (2.92)	H-bonding	**−15.6**
	NH_2_	H_2_O-Ser342 (2.55)	H-bonding	
	MeO	Lys295, H_2_O-Asn391, Asp404 (2.51) (2.57)	H-bonding	
	Me	Asn391, Asp404	Hydrophobic	
	MeO-Naphthalene	Val281, Leu273	Hydrophobic	

The data reported in the table are extracted from MOE program showing the corresponding amino acids residues in enzyme pocket, corresponding fragments of ligands, interaction distances, types of interaction, and their binding energy to some selected drugs. Conserved amino acids are bold highlighted.

### 3.8. Prediction of drug-likeness and ADME properties

In a way to design a drug with a good oral bioavailability profile, certain parameters are considered to play an important role^46^^,^^47^. About one-third of the invented drugs fail during development processes due to their unreasonable pharmacokinetic profiles^48^. Therefore, a computational study for the prediction of the ADME properties of the synthesised molecules was performed by the calculation of lipophilicity, topological polar surface area (TPSA), absorption (% ABS), and simple molecular descriptors used by Lipinski in formulating his “rule of five”^49^. Calculations were performed using the Molinspiration online property calculation toolkit^50^. Table 10 represents a calculated percentage of the absorption (% ABS), TPSA, and Lipinski parameters of the compounds. A percentage of absorption (% ABS) was estimated using the equation: % ABS =109−(0.345 × TPSA), according to Zhao et al.^51^ polar surface area, together with lipophilicity, is an essential property of a molecule of transportation across biological membranes. Extremely high TPSA values give rise to poor bioavailability and absorption of a drug. According to the above criterions, calculated percentages of absorption for compounds are ranged between 88.6% and 79.1%. The number of the hydrogen bond donors was constant for all of the compounds, and the number of hydrogen bond acceptors varied from 1 to 4. The investigation of the Lipinski profile of the synthesised compounds indicated that most of the synthesised derivatives are considered to be drug-like candidates for the novel cytotoxic agents with a slight increase in the log *p* values for two bromobenzochromene compounds.

**Table 10.** Predicted ADME, Lipinski parameters, and molecular properties of the synthesised compounds.

Cpd.	M. wt.	Log*S*	*n*-ON	*n*-OHNH	TPSA	Log*P*(o/w)	%ABS
**4a**	377	−7.3	1	1	59	4.8	88.6
**4b**	395	−7.6	1	1	59	4.9	88.6
**4c**	412	−8	1	1	59	5.4	88.6
**4d**	456	−8.3	1	1	59	5.6	88.6
**4e**	407	−7.3	2	1	68	4.7	85.4
**4f**	391	−7.7	1	1	59	5.1	88.6
**4g**	437	−7.4	3	1	78	4.7	82.3
**4h**	437	−7.4	3	1	78	4.5	82.3
**6a**	328	−6.2	2	1	68	3.9	85.4
**6b**	346	−6.5	2	1	68	4.1	85.4
**6c**	363	−6.9	2	1	68	4.5	85.4
**6d**	407	−7.3	2	1	68	4.7	85.4
**6e**	342	−6.7	2	1	68	4.2	85.4
**6f**	358	−6.3	3	1	78	3.9	82.3
**6g**	388	−6.3	4	1	87	3.9	79.1
**6h**	388	−6.3	4	1	87	3.6	79.1

% ABS: Percentage of absorption; TPSA: topological polar surface area; *n*-ON: number of hydrogen bond acceptors; *n*-OHNH: number of hydrogen bond donors; log*S*: solubility coefficient; Log *P*: partition coefficient. Calculations were performed using Molinspiration online property calculation toolkit (http://www.molinspiration.com).

## Supplementary Material

Supplemental Material

## 4. Conclusions

Herein, we present the synthesis of 16 compounds of 3-amino-1-aryl-8-bromo/methoxy-1*H*-benzo[*f*]chromene-2-carbonitrile **4a–h** and **6a–h**. The new compounds have been evaluated for their cytotoxic activities against three cancer cell lines, MCF-7, HCT-116, and HepG-2. The obtained results claimed that most of the synthesised compounds displayed strong and selective cytotoxic activity against the three cell lines. Moreover, the potent compounds have been examined for their cytotoxic effect against normal fibroblast cell line and revealed weak cytotoxicity compared to their anticancer effect. These results confirm the selectivity behaviour of the target compounds. On the other hand, the SAR study has been performed on the desired compounds and elucidated that the introduction of halogens substituent (electron-withdrawing group) at the 4-position of the phenyl group at position 1 of the 1*H*-benzo[*f*]chromene moiety enhanced their antitumour activities than the electron-donating substituent like methyl or methoxy groups. In addition, the presence of the lipophilic electron-donating, methoxy group at the 8-position was essential for the activity than the electron-withdrawing substituent like a bromine atom. Caspase 3/7 intrinsic apoptotic assay has been used to explore the demise of the three cell lines upon their treatment with the target compounds. Meanwhile, the interaction behaviour of selected active benzochromene derivatives was examined computationally via their docking simulation within the *c-Src* kinase target, which resulted in a good interaction profile compared to the reference ligand. This behaviour classifies these molecules as potential *c-Src* kinase candidates with potent cytotoxic activity.
