# Supplementary material for: Introducing novel potent anticancer agents of 1H-benzo[f]chromene scaffolds, targeting c-Src kinase enzyme with MDA-MB-231 cell line anti-invasion effect
Source: J Enzyme Inhib Med Chem. 2018 Jun 20;33(1):1074–88. doi: 10.1080/14756366.2018.1476503 (PMC6022228; doi:10.1080/14756366.2018.1476503)
Supplement: Supplemental Material [file IENZ_A_1476503_SM6629.pdf]

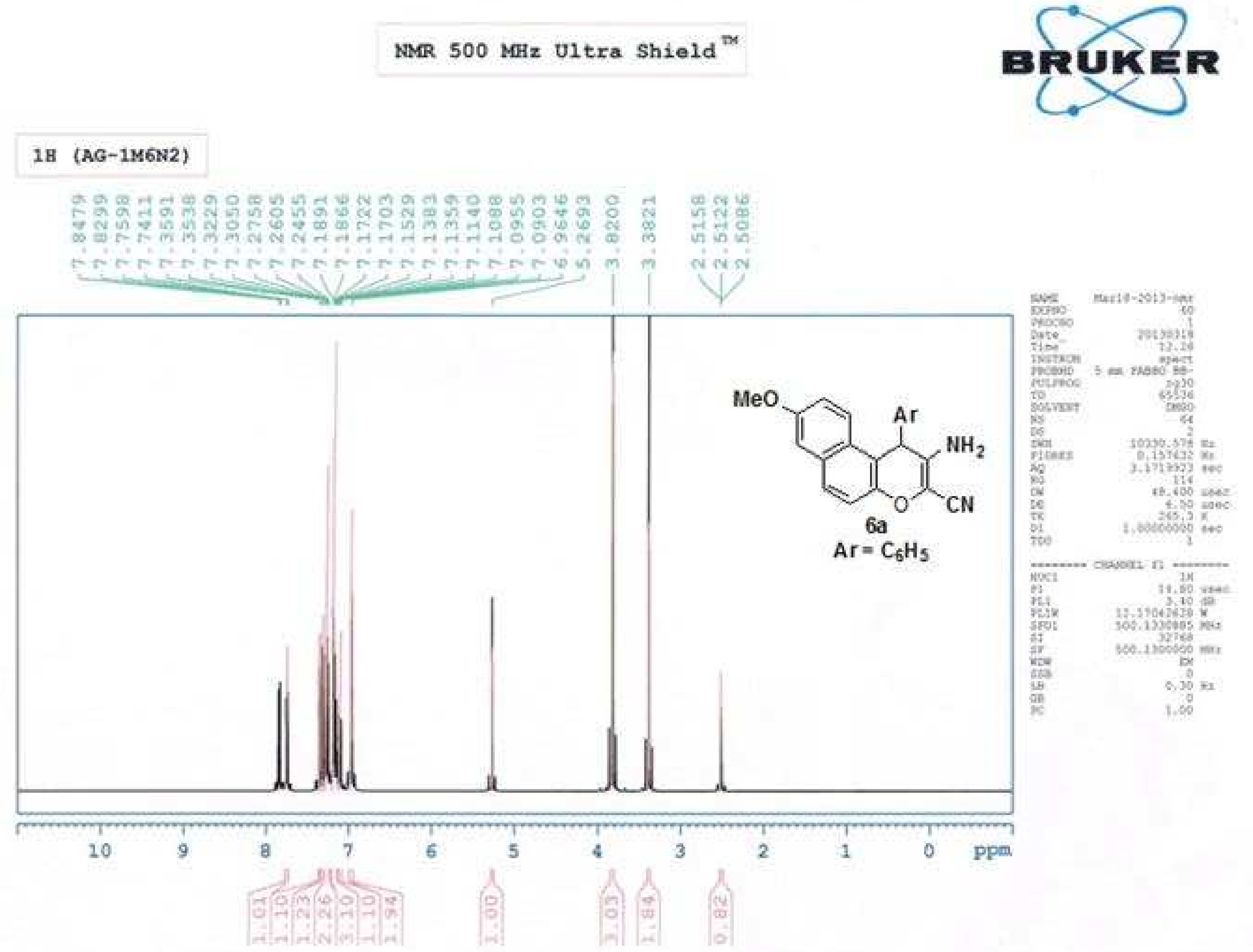
NMR 500 MHz Ultra Shield™
1H (AG-1M6N2)
7.8479
7.8299
7.7598
7.7411
7.3591
7.3538
7.3229
7.3050
7.2758
7.2605
7.2455
7.1891
7.1866
7.1722
7.1703
7.1529
7.1383
7.1359
7.1140
7.1088
7.0955
7.0903
6.9646
5.2693
3.8200
3.3821
2.5158
2.5122
2.5086
MeO
Ar
NH2
CN
O
6a
Ar = C6H5
10
9
8
7
6
5
4
3
2
1
0
ppm
1.01
1.10
1.23
2.26
3.10
1.10
1.94
1.00
3.03
1.84
0.82

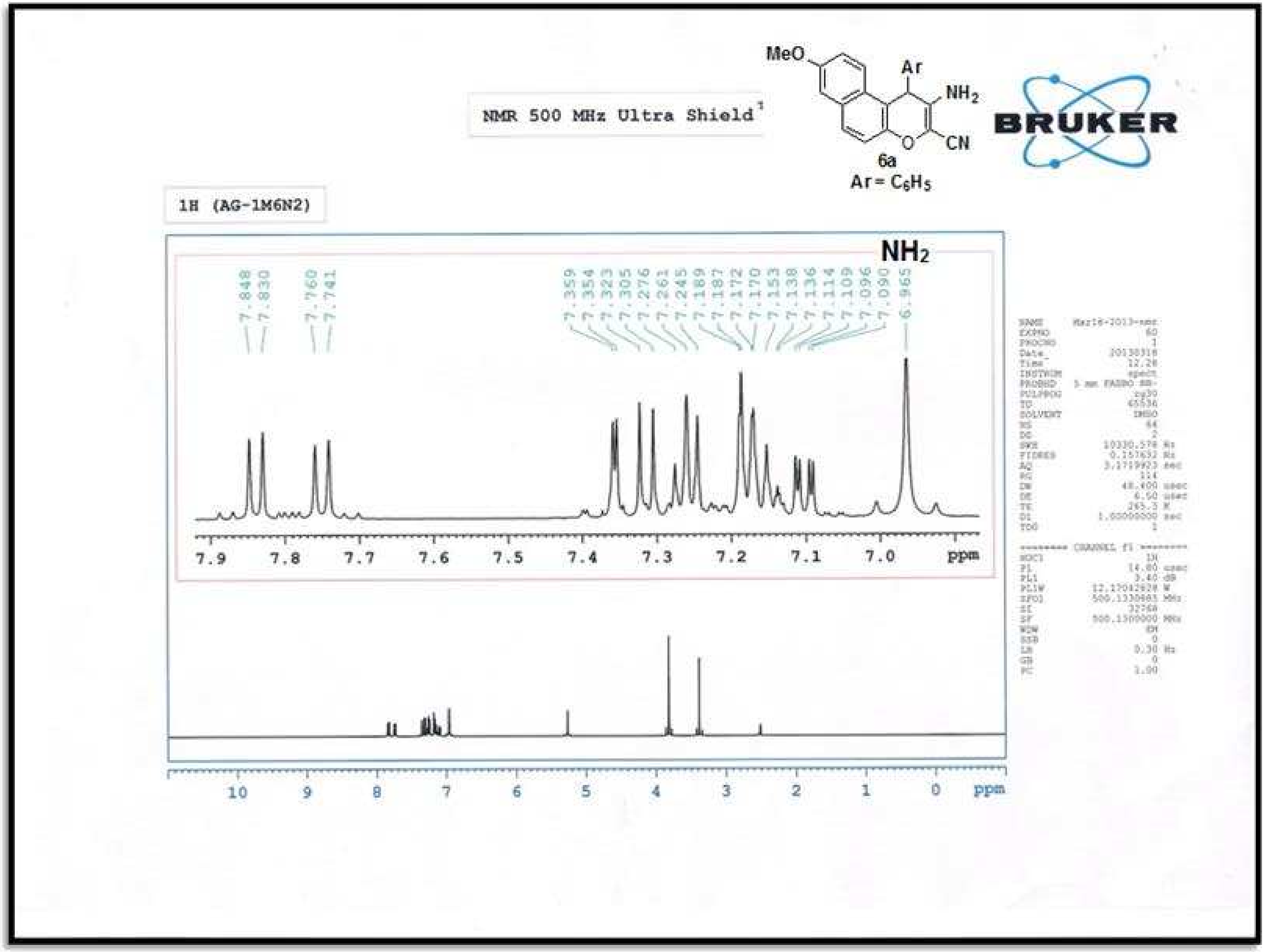
NMR 500 MHz Ultra Shield
MeO
Ar
NH2
CN
O
6a
Ar = C6H5
1H (AG-1M6N2)
NH2
7.848
7.830
7.760
7.741
7.359
7.354
7.323
7.305
7.276
7.261
7.245
7.189
7.187
7.172
7.170
7.153
7.138
7.136
7.114
7.109
7.096
7.090
6.965
ppm
ppm

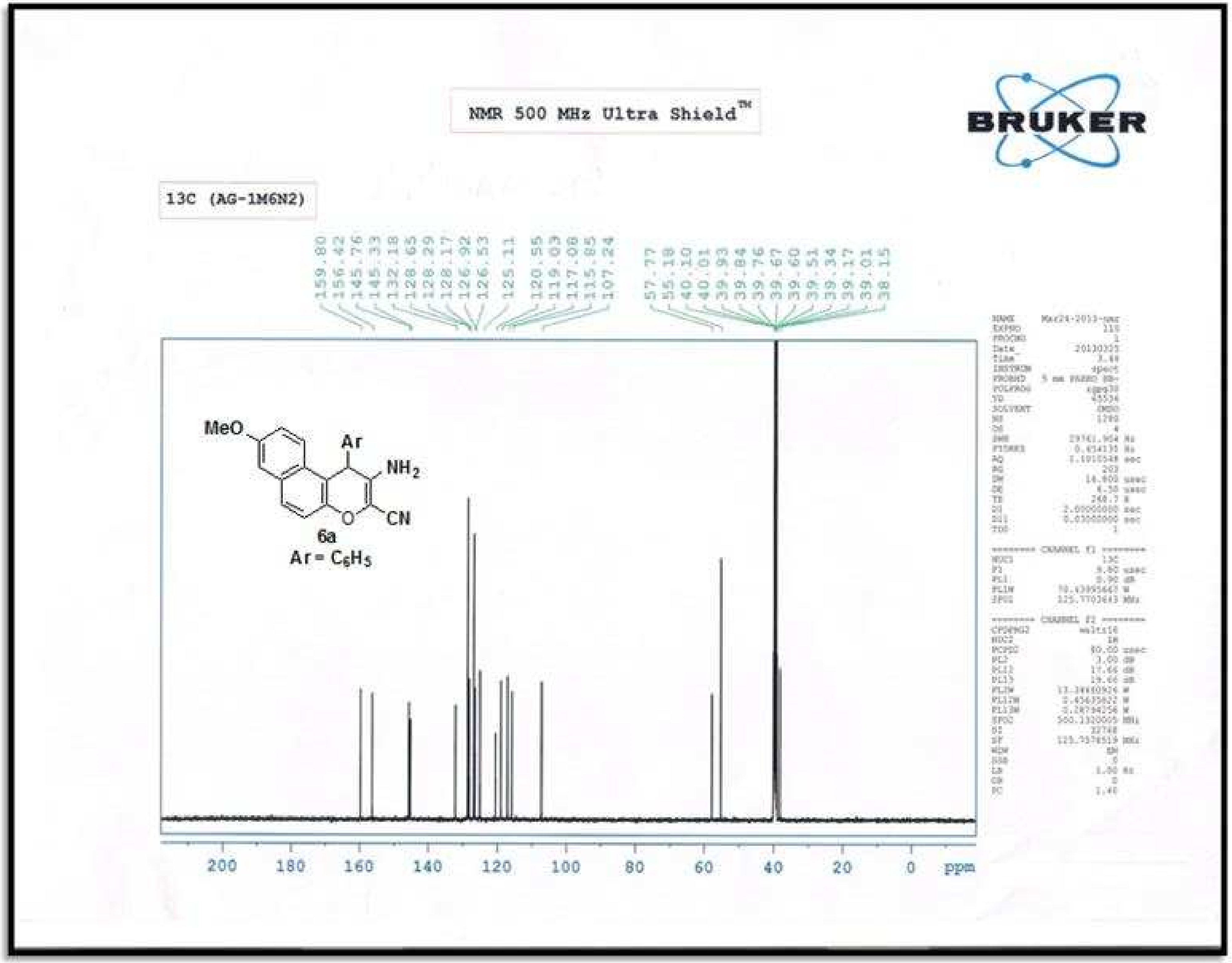

NMR 500 MHz Ultra Shield™
BRUKER
13C (AG-1M6N2)
159.80
156.42
145.76
145.33
132.18
128.65
128.29
128.17
126.92
126.53
125.11
120.55
119.03
117.08
115.85
107.24
57.77
55.18
40.10
40.01
39.93
39.84
39.76
39.67
39.60
39.51
39.34
39.17
39.01
38.15
MeO
Ar
NH2
CN
O
6a
Ar = C6H5
200
180
160
140
120
100
80
60
40
20
0
ppm

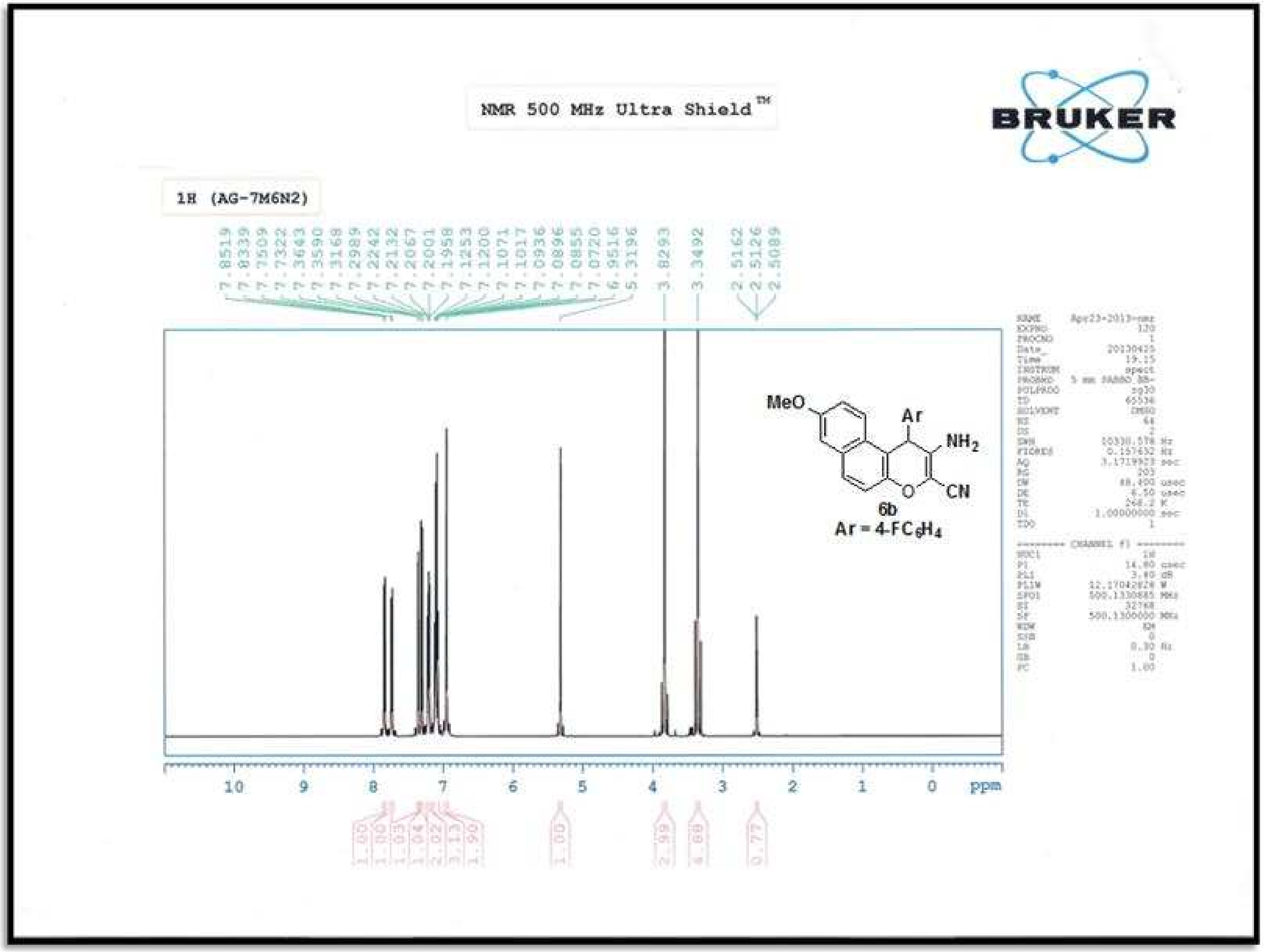
NMR 500 MHz Ultra Shield™
BRUKER
1H (AG-7M6N2)
7.8519
7.8339
7.7509
7.7322
7.3643
7.3590
7.3168
7.2989
7.2242
7.2132
7.2067
7.2001
7.1958
7.1253
7.1200
7.1071
7.1017
7.0936
7.0896
7.0855
7.0720
6.9516
5.3196
3.8293
3.3492
2.5162
2.5126
2.5089
MeO
Ar
NH2
CN
O
6b
Ar = 4-FC6H4
10
9
8
7
6
5
4
3
2
1
0
ppm
1.00
1.00
1.03
1.04
2.02
3.13
1.90
1.00
2.99
4.88
0.77

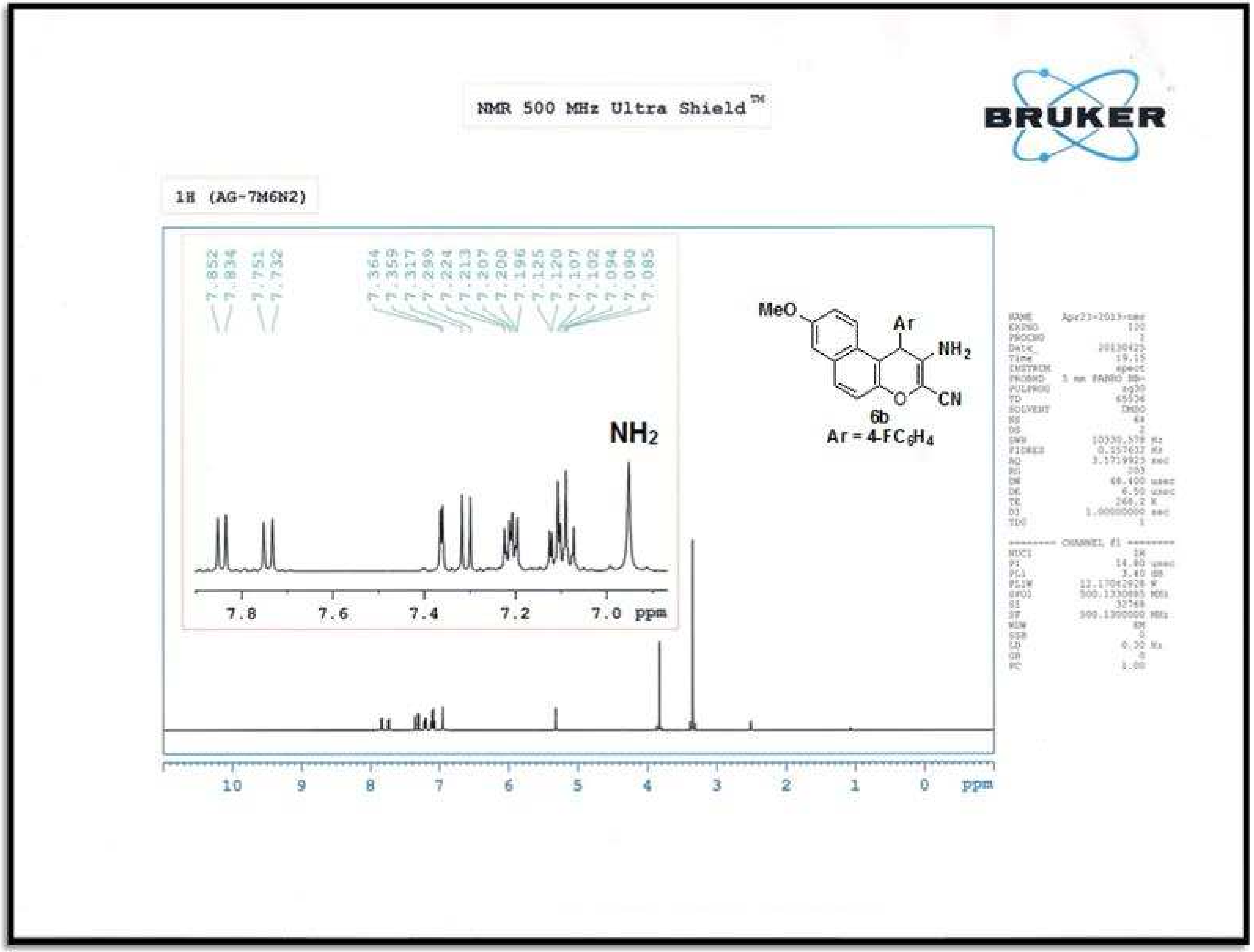
NMR 500 MHz Ultra Shield™
BRUKER
1H (AG-7M6N2)
7.852
7.834
7.751
7.732
7.364
7.359
7.317
7.299
7.224
7.213
7.207
7.200
7.196
7.125
7.120
7.107
7.102
7.094
7.090
7.085
NH2
7.8
7.6
7.4
7.2
7.0
ppm
MeO
Ar
NH2
O
CN
6b
Ar = 4-FC6H4
10
9
8
7
6
5
4
3
2
1
0
ppm

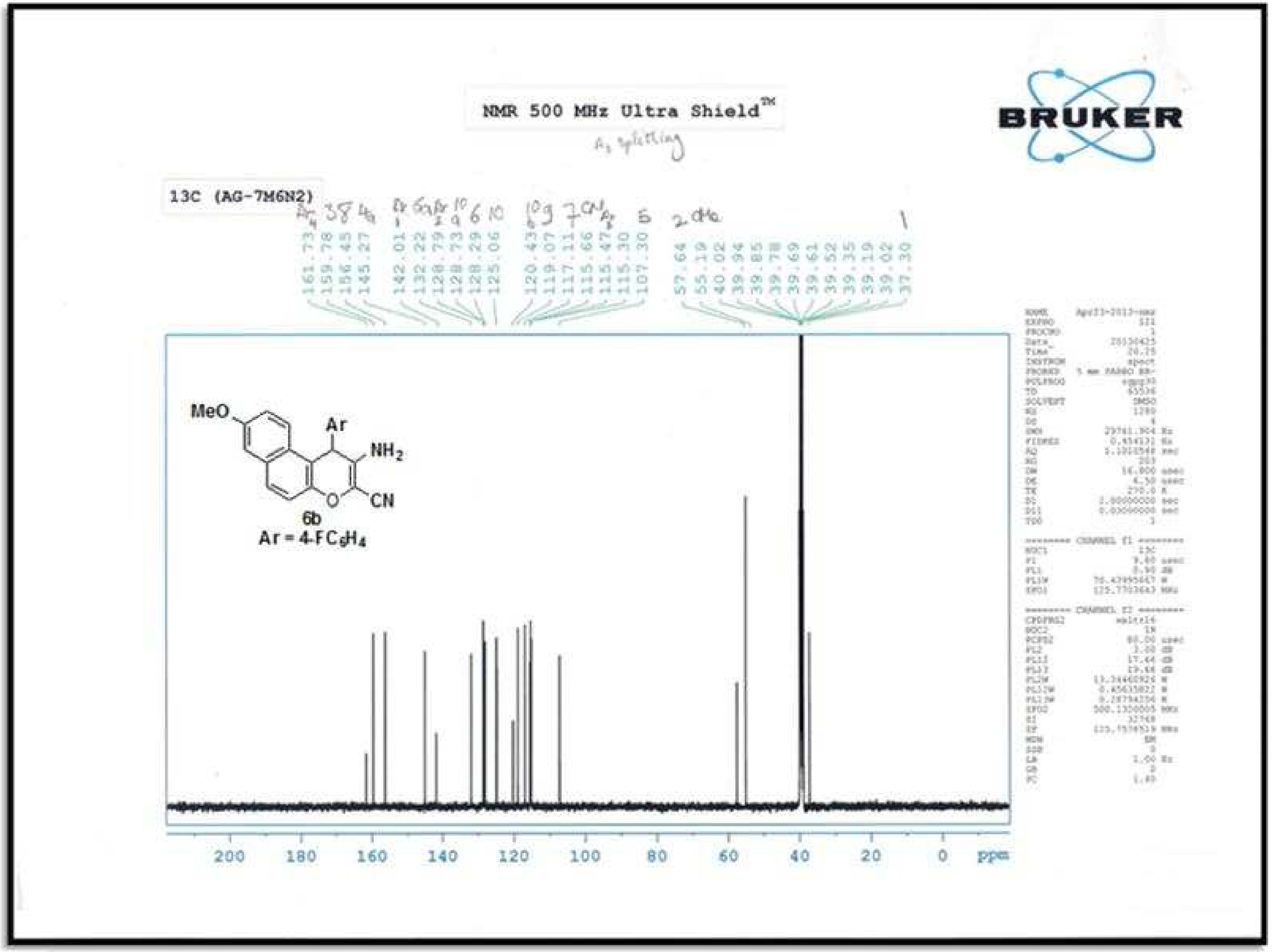
NMR 500 MHz Ultra Shield™
BRUKER
13C (AG-7M6N2)
161.73
159.78
156.45
145.27
142.01
132.22
128.79
128.73
128.29
125.06
120.43
119.07
117.11
115.66
115.47
115.30
107.30
57.64
55.19
40.02
39.94
39.85
39.78
39.69
39.61
39.52
39.35
39.19
39.02
37.30
MeO
Ar
NH2
CN
6b
Ar = 4-FC6H4
200
180
160
140
120
100
80
60
40
20
0
ppm

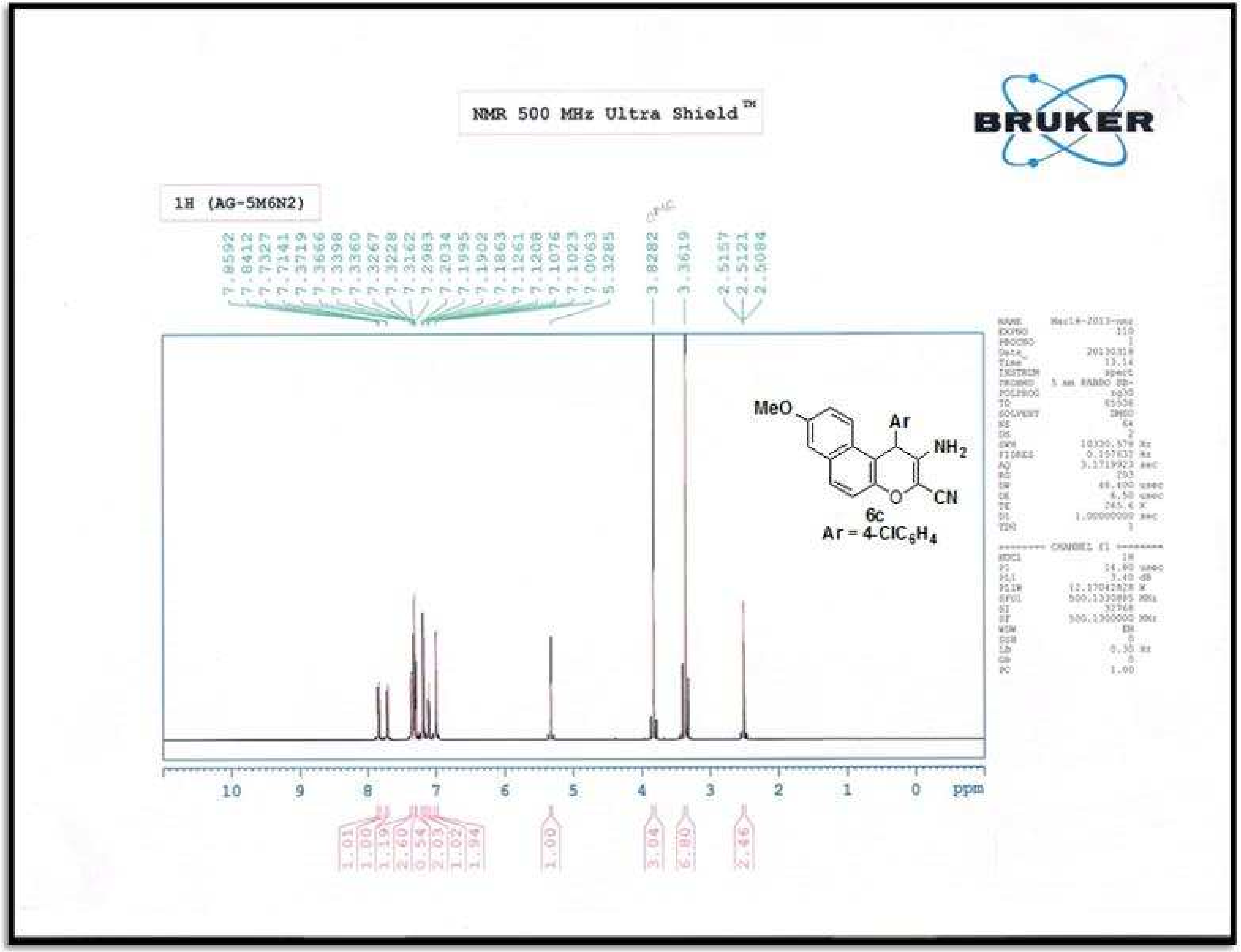

NMR 500 MHz Ultra Shield™
BRUKER
1H (AG-5M6N2)
7.8592
7.8412
7.7327
7.7141
7.3719
7.3666
7.3398
7.3360
7.3267
7.3228
7.3162
7.2983
7.2034
7.1995
7.1902
7.1863
7.1261
7.1208
7.1076
7.1023
7.0063
5.3285
3.8282
3.3619
2.5157
2.5121
2.5084
MeO
Ar
NH2
CN
O
6c
Ar = 4-ClC6H4
10
9
8
7
6
5
4
3
2
1
0
ppm
1.01
1.00
1.19
2.60
0.54
2.03
1.02
1.94
1.00
3.04
6.80
2.46

NMR 500 MHz Ultra Shield™

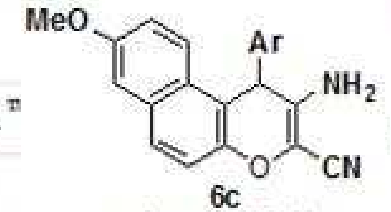

6c
Ar = 4-ClC$_6$H$_4$

1H (AG-5M6N2)

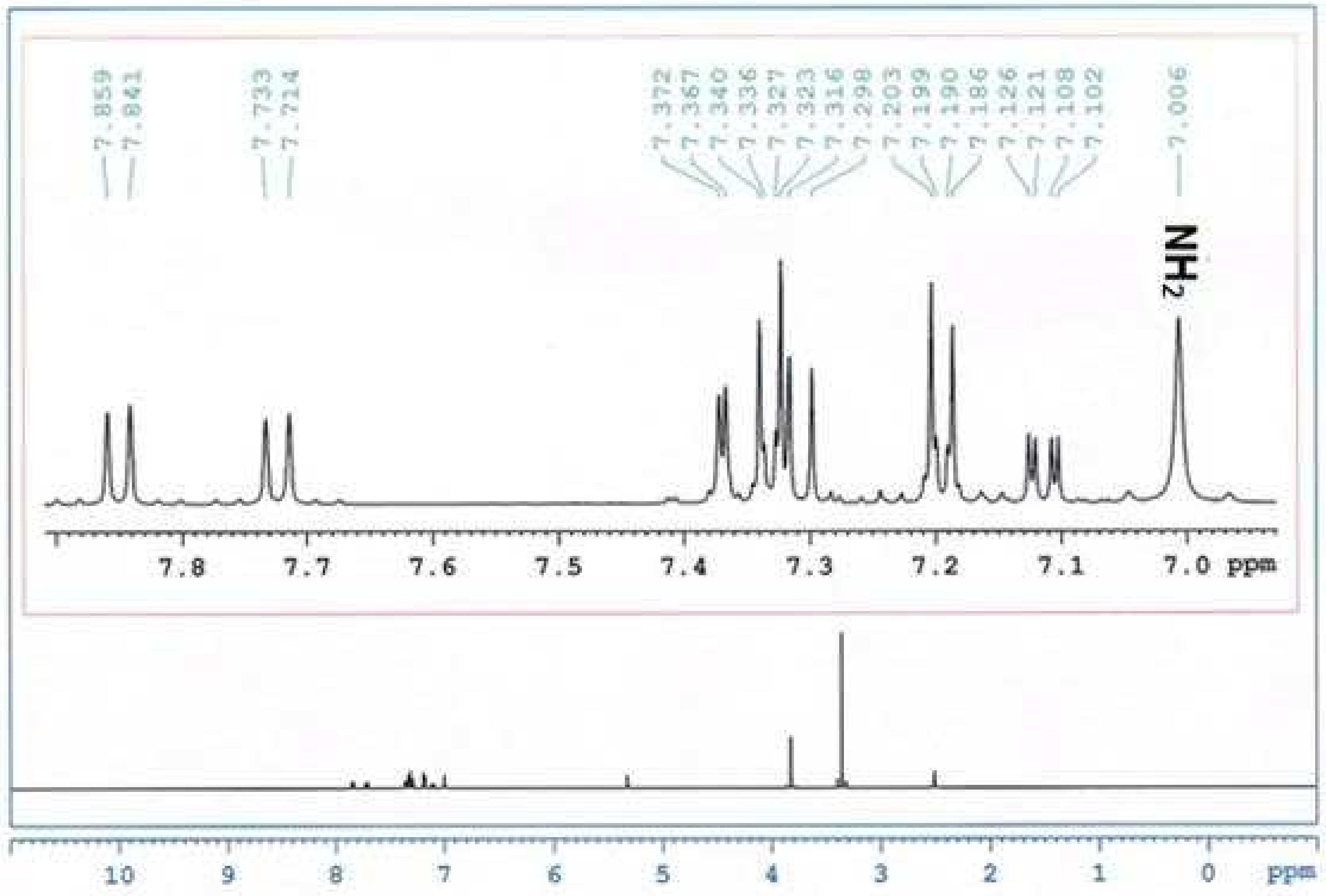

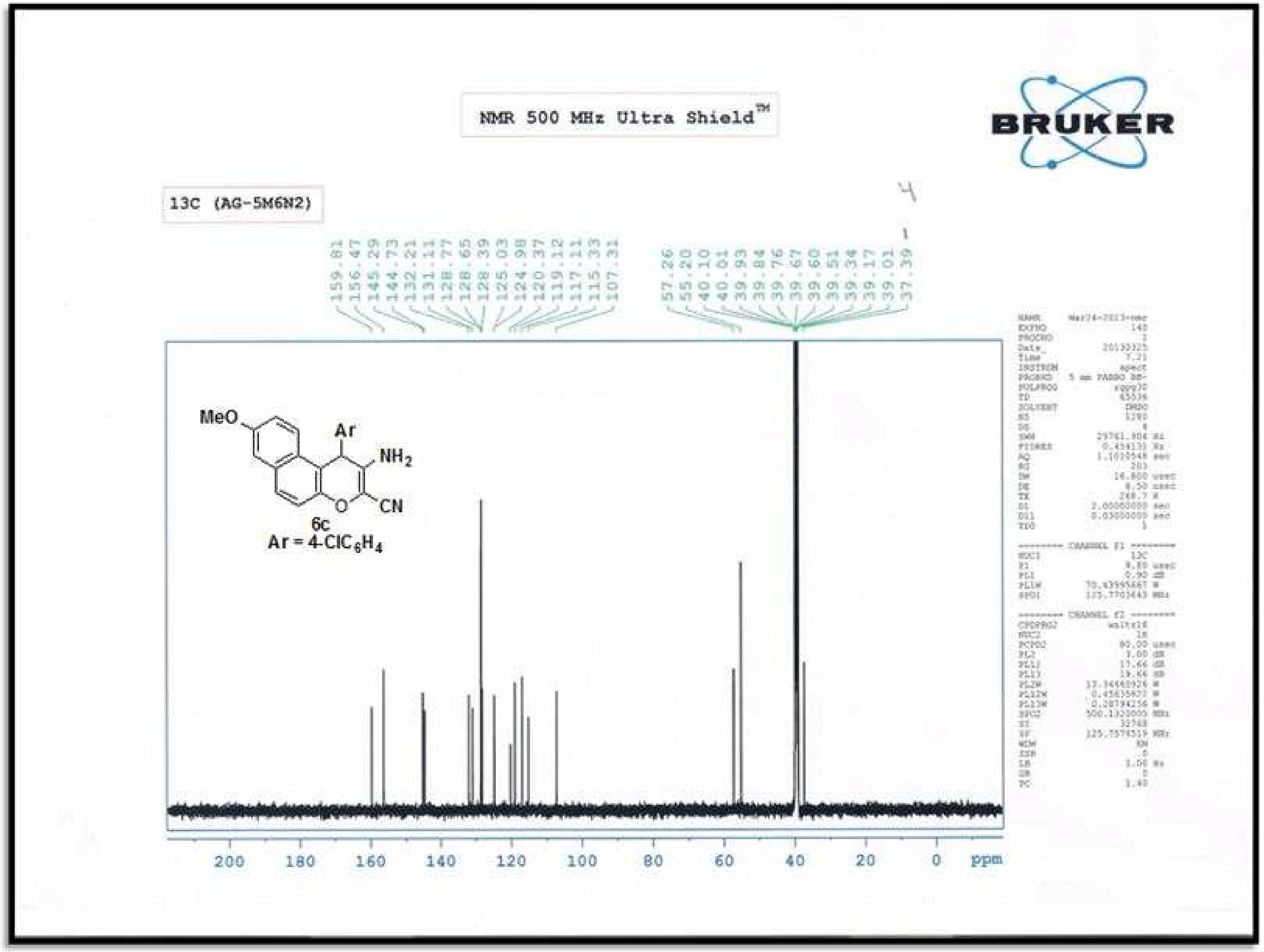
NMR 500 MHz Ultra Shield™
BRUKER
13C (AG-5M6N2)
159.81
156.47
145.29
144.73
132.21
131.11
128.77
128.65
128.39
125.03
124.98
120.37
119.12
117.11
115.33
107.31
57.26
55.20
40.10
40.01
39.93
39.84
39.76
39.67
39.60
39.51
39.34
39.17
39.01
37.39
MeO
Ar
NH2
CN
O
6c
Ar = 4-ClC6H4
200
180
160
140
120
100
80
60
40
20
0
ppm

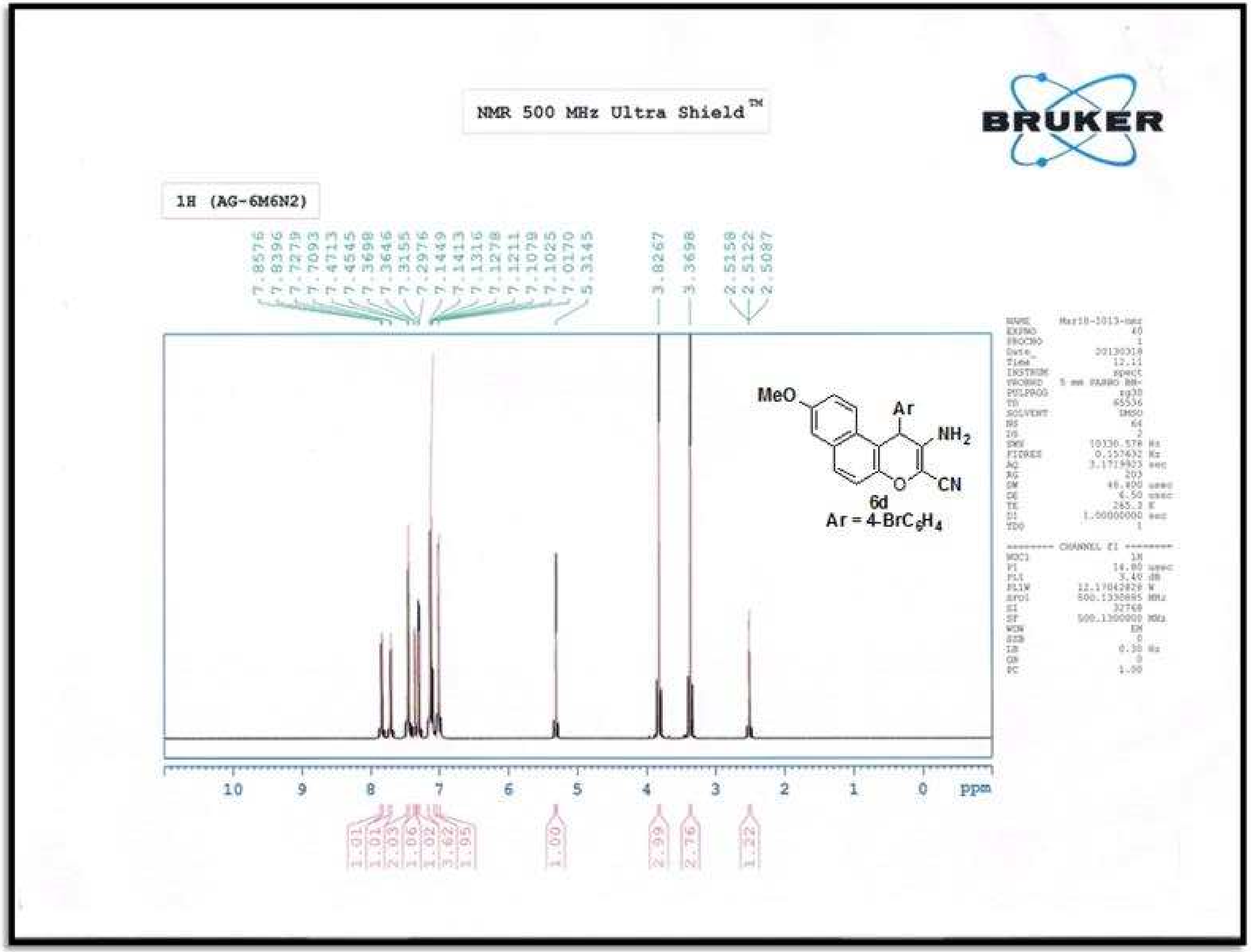
NMR 500 MHz Ultra Shield™
BRUKER
1H (AG-6M6N2)
7.8576 7.8396 7.7279 7.7093 7.4713 7.4545 7.3698 7.3646 7.3155 7.2976 7.1449 7.1413 7.1316 7.1278 7.1211 7.1079 7.1025 7.0170 5.3145
3.8267 3.3698
2.5158 2.5122 2.5087
MeO
Ar
NH2
CN
6d
Ar = 4-BrC6H4
10 9 8 7 6 5 4 3 2 1 0 ppm
1.01 1.01 2.03 1.06 1.02 3.62 1.95
1.00
2.99 2.76
1.22

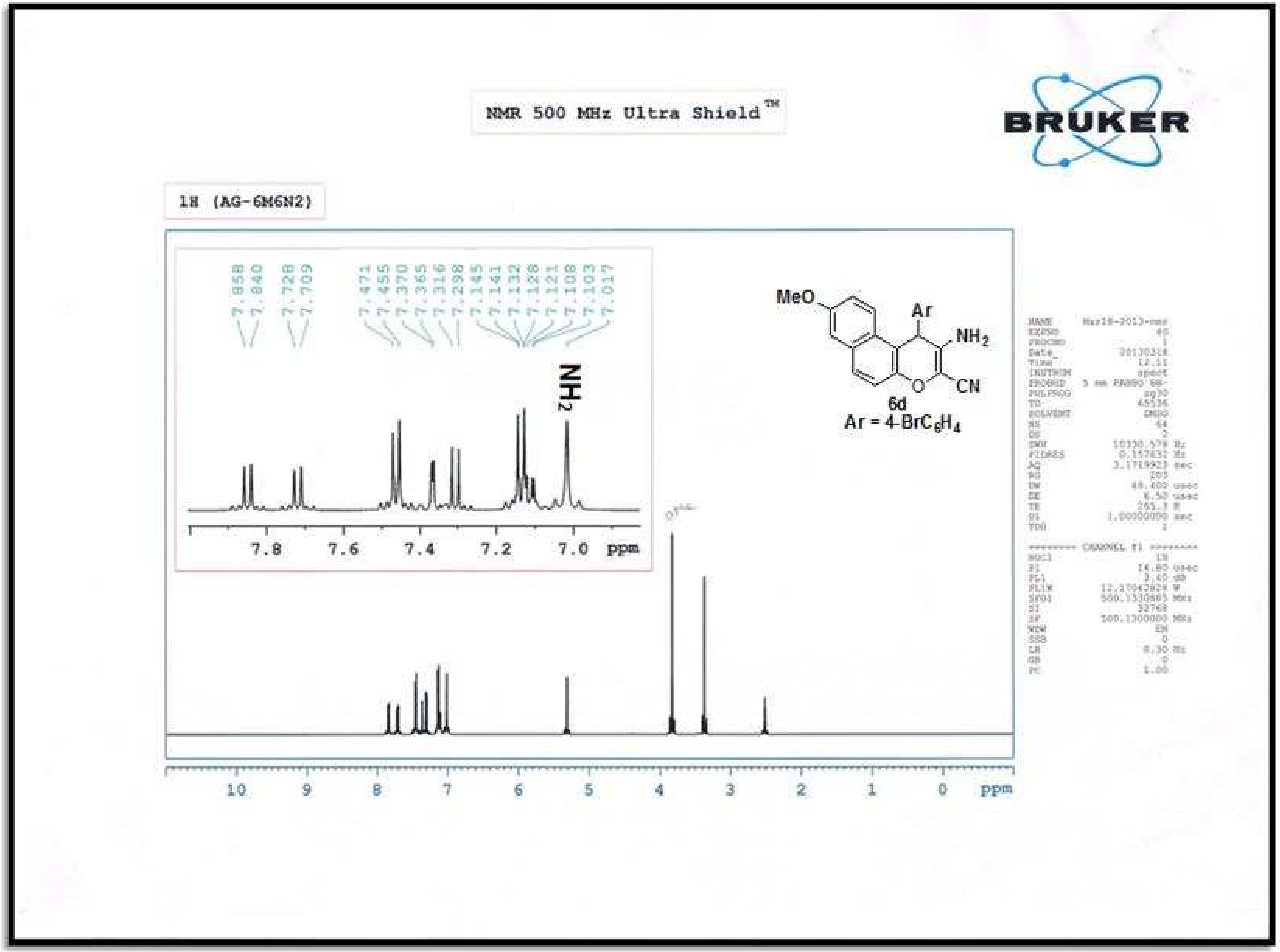
NMR 500 MHz Ultra Shield™
BRUKER
1H (AG-6M6N2)
7.858
7.840
7.728
7.709
7.471
7.455
7.370
7.365
7.316
7.298
7.145
7.141
7.132
7.128
7.121
7.108
7.103
7.017
NH2
7.8
7.6
7.4
7.2
7.0
ppm
MeO
Ar
NH2
CN
O
6d
Ar = 4-BrC6H4
10
9
8
7
6
5
4
3
2
1
0
ppm

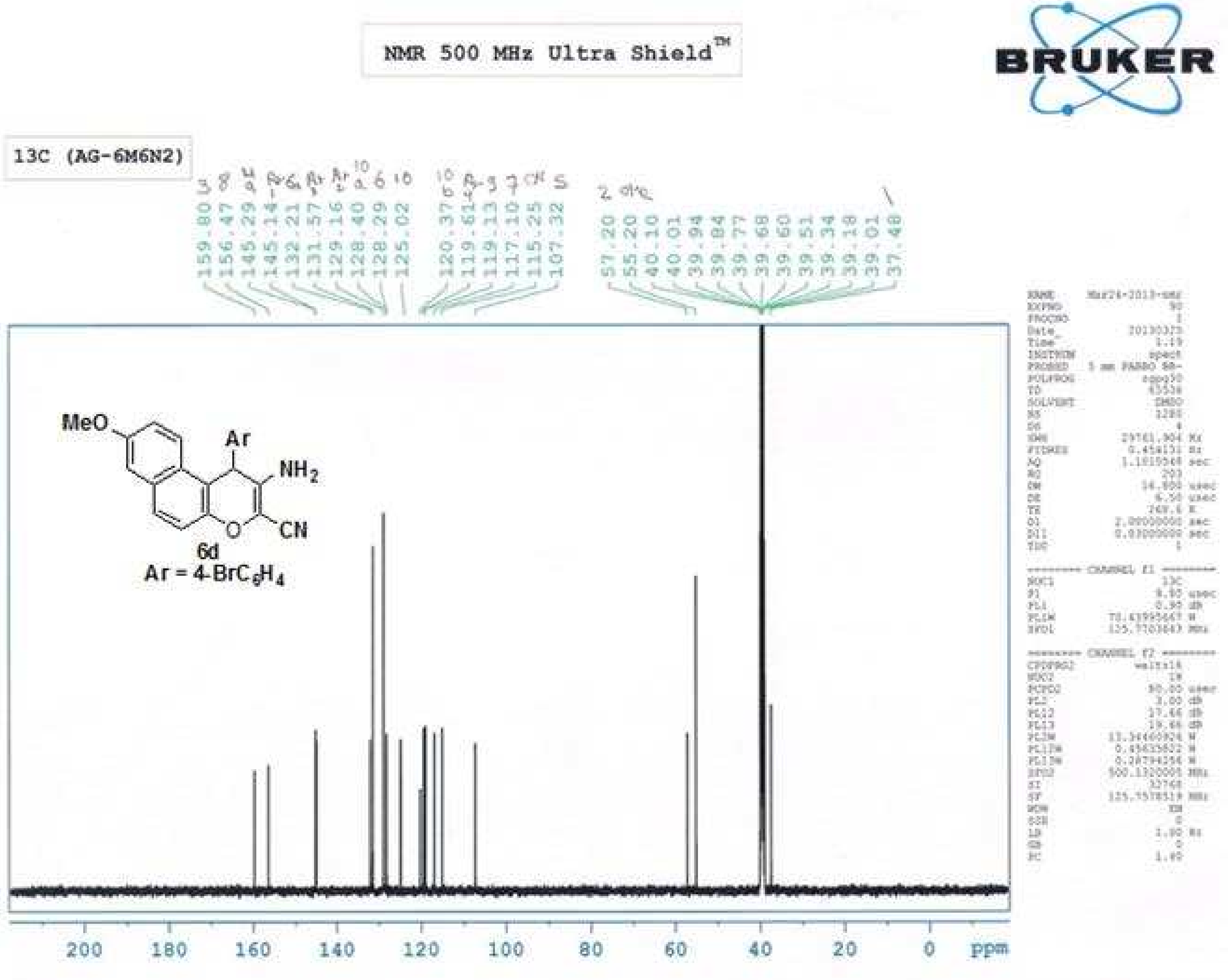
NMR 500 MHz Ultra Shield™
13C (AG-6M6N2)
159.80
156.47
145.29
145.14
132.21
131.57
129.16
128.40
128.29
125.02
120.37
119.61
119.13
117.10
115.25
107.32
57.20
55.20
40.10
40.01
39.94
39.84
39.77
39.68
39.60
39.51
39.34
39.18
39.01
37.48
MeO
Ar
NH2
CN
O
6d
Ar = 4-BrC6H4
200 180 160 140 120 100 80 60 40 20 0 ppm

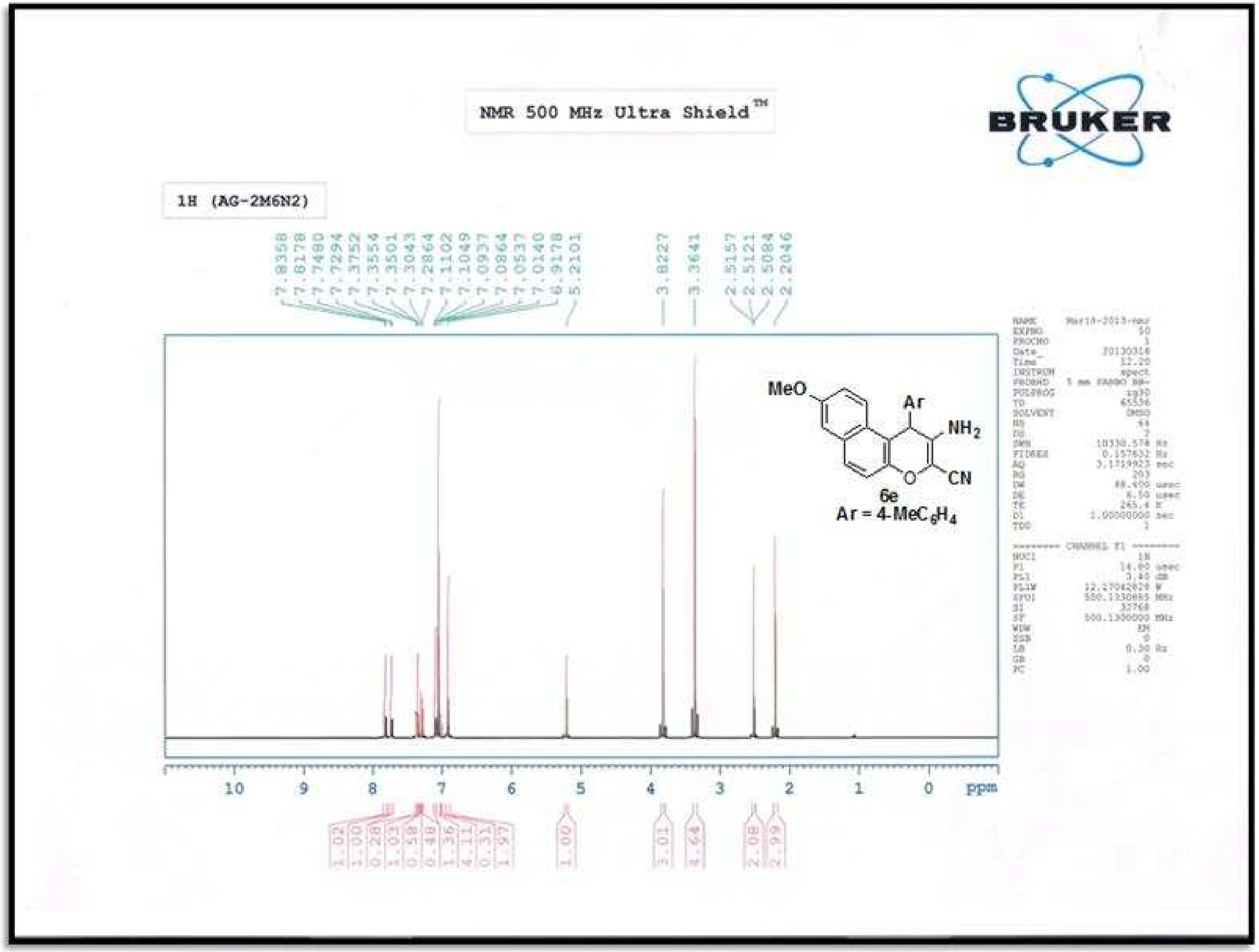

NMR 500 MHz Ultra Shield™
BRUKER
1H (AG-2M6N2)
7.8358
7.8178
7.7480
7.7294
7.3752
7.3554
7.3501
7.3043
7.2864
7.1102
7.1049
7.0937
7.0864
7.0537
7.0140
6.9178
5.2101
3.8227
3.3641
2.5157
2.5121
2.5084
2.2046
MeO
Ar
NH2
CN
O
6e
Ar = 4-MeC6H4
10
9
8
7
6
5
4
3
2
1
0
ppm
1.02
1.00
0.28
1.03
0.58
0.48
1.36
4.11
0.31
1.97
1.00
3.01
4.64
2.08
2.99

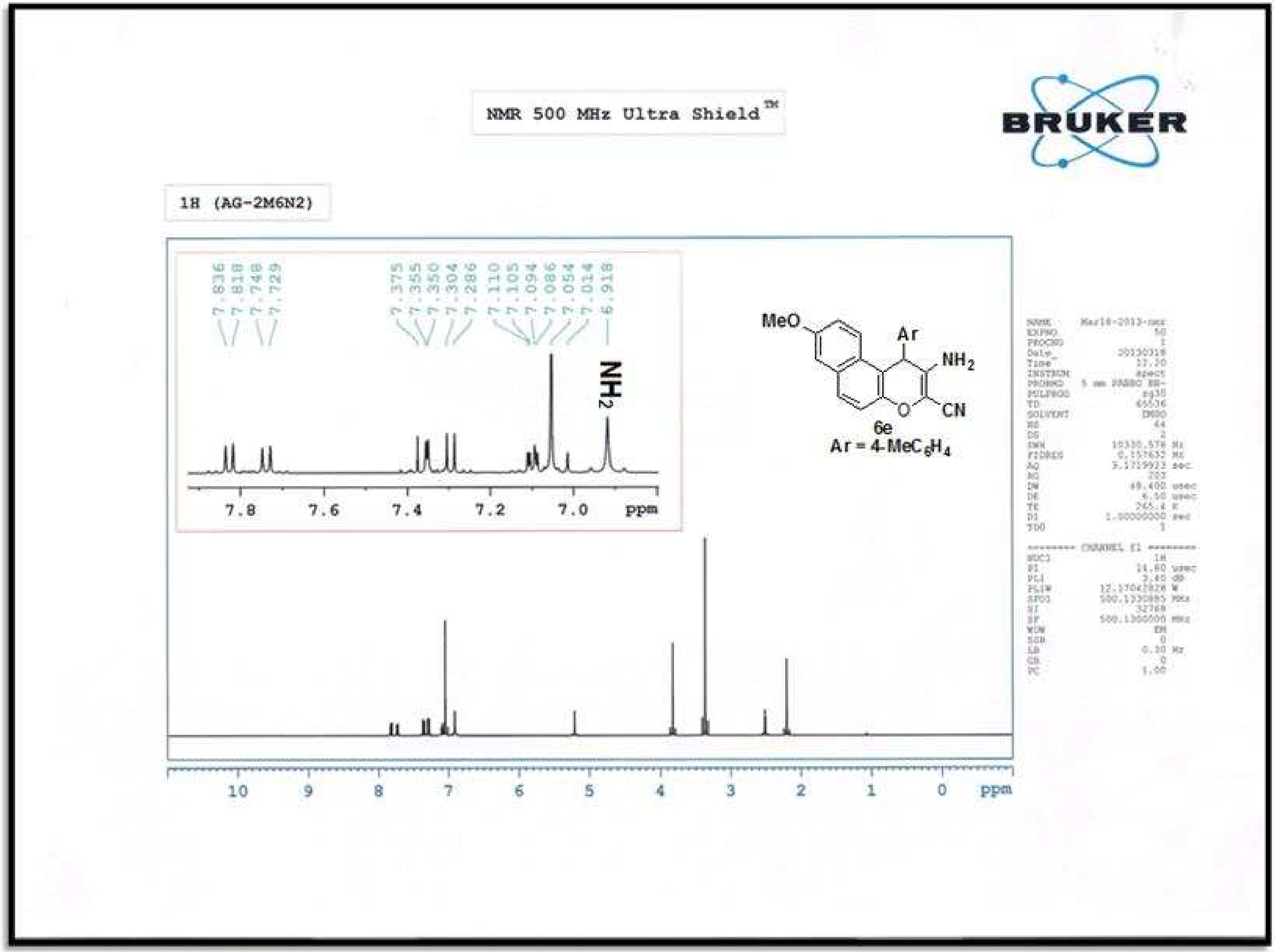
NMR 500 MHz Ultra Shield™
BRUKER
1H (AG-2M6N2)
7.836
7.818
7.748
7.729
7.375
7.355
7.350
7.304
7.286
7.110
7.105
7.094
7.086
7.054
7.014
6.918
NH2
MeO
Ar
NH2
CN
6e
Ar = 4-MeC6H4
ppm

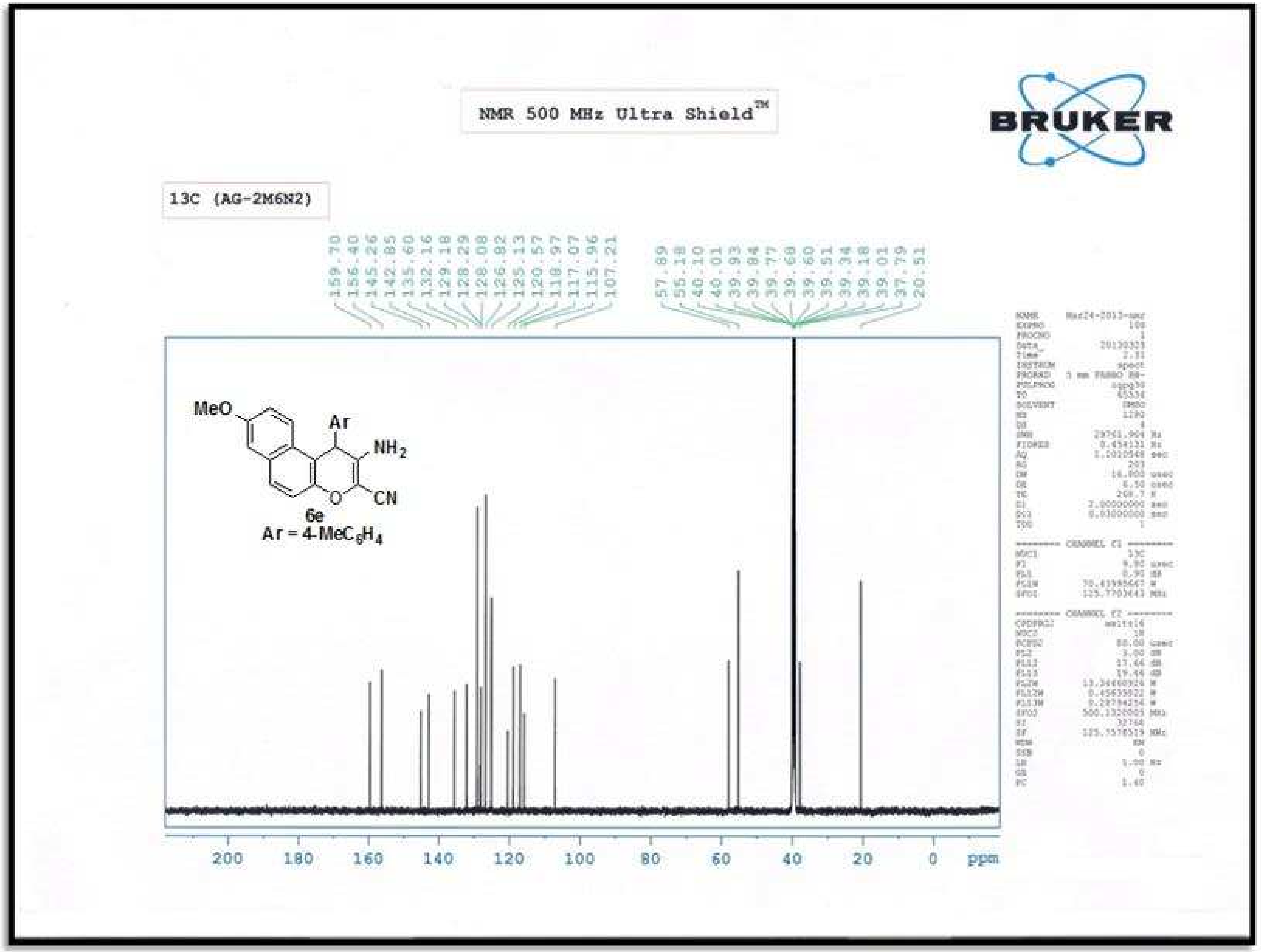

NMR 500 MHz Ultra Shield™
BRUKER
13C (AG-2M6N2)
159.70
156.40
145.26
142.85
135.60
132.16
129.18
128.29
128.08
126.82
125.13
120.57
118.97
117.07
115.96
107.21
57.89
55.18
40.10
40.01
39.93
39.84
39.77
39.68
39.60
39.51
39.34
39.18
39.01
37.79
20.51
MeO
Ar
NH2
CN
O
6e
Ar = 4-MeC6H4
200
180
160
140
120
100
80
60
40
20
0
ppm

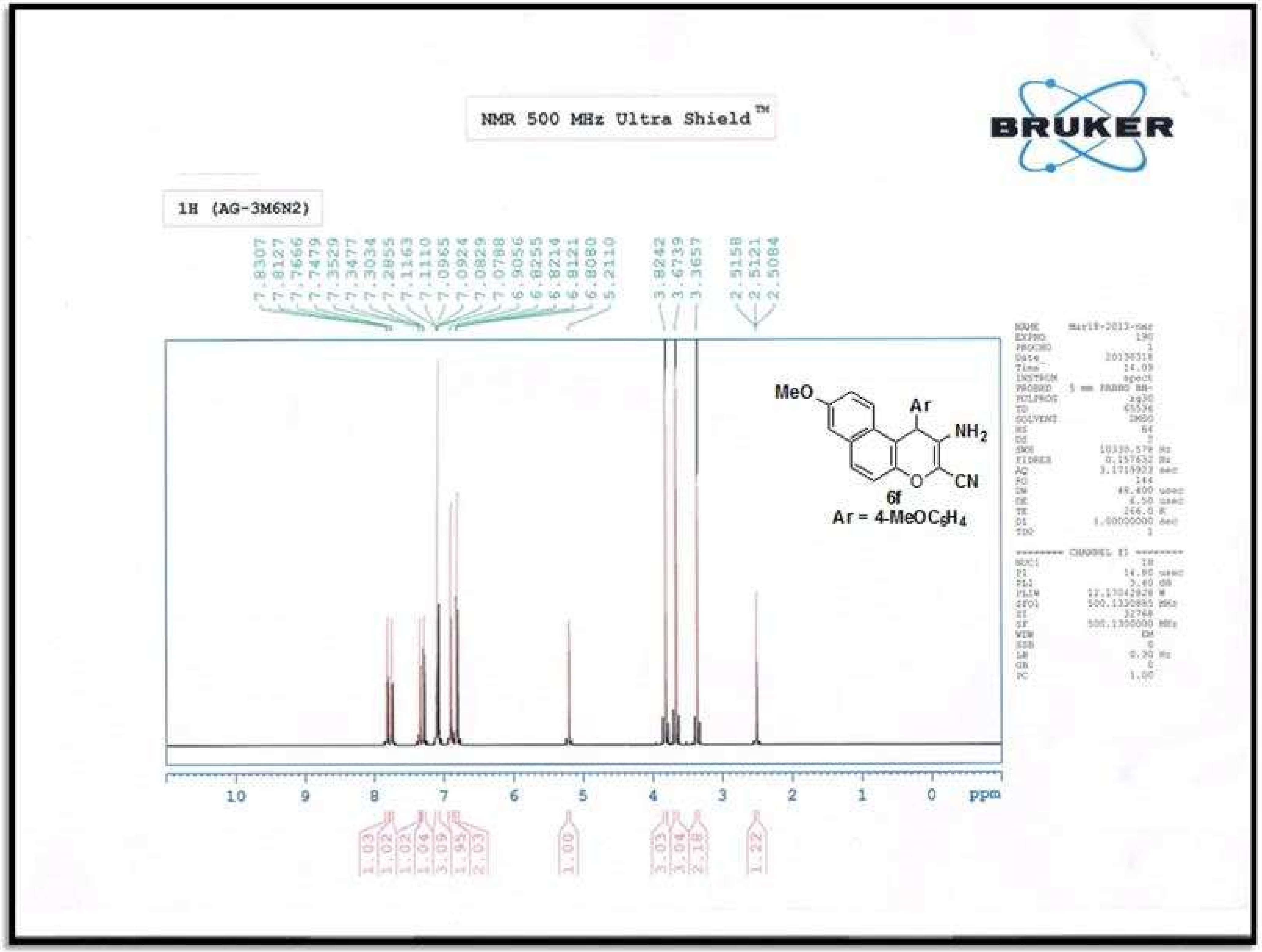

NMR 500 MHz Ultra Shield™
BRUKER
1H (AG-3M6N2)
7.8307
7.8127
7.7666
7.7479
7.3529
7.3477
7.3034
7.2855
7.1163
7.1110
7.0965
7.0924
7.0829
7.0788
6.9056
6.8255
6.8214
6.8121
6.8080
5.2110
3.8242
3.6739
3.3657
2.5158
2.5121
2.5084
MeO
Ar
NH2
CN
O
6f
Ar = 4-MeOC6H4
10 9 8 7 6 5 4 3 2 1 0 ppm
1.03 1.02 1.02 1.04 3.09 1.95 2.03 1.00 3.03 3.04 2.18 1.22

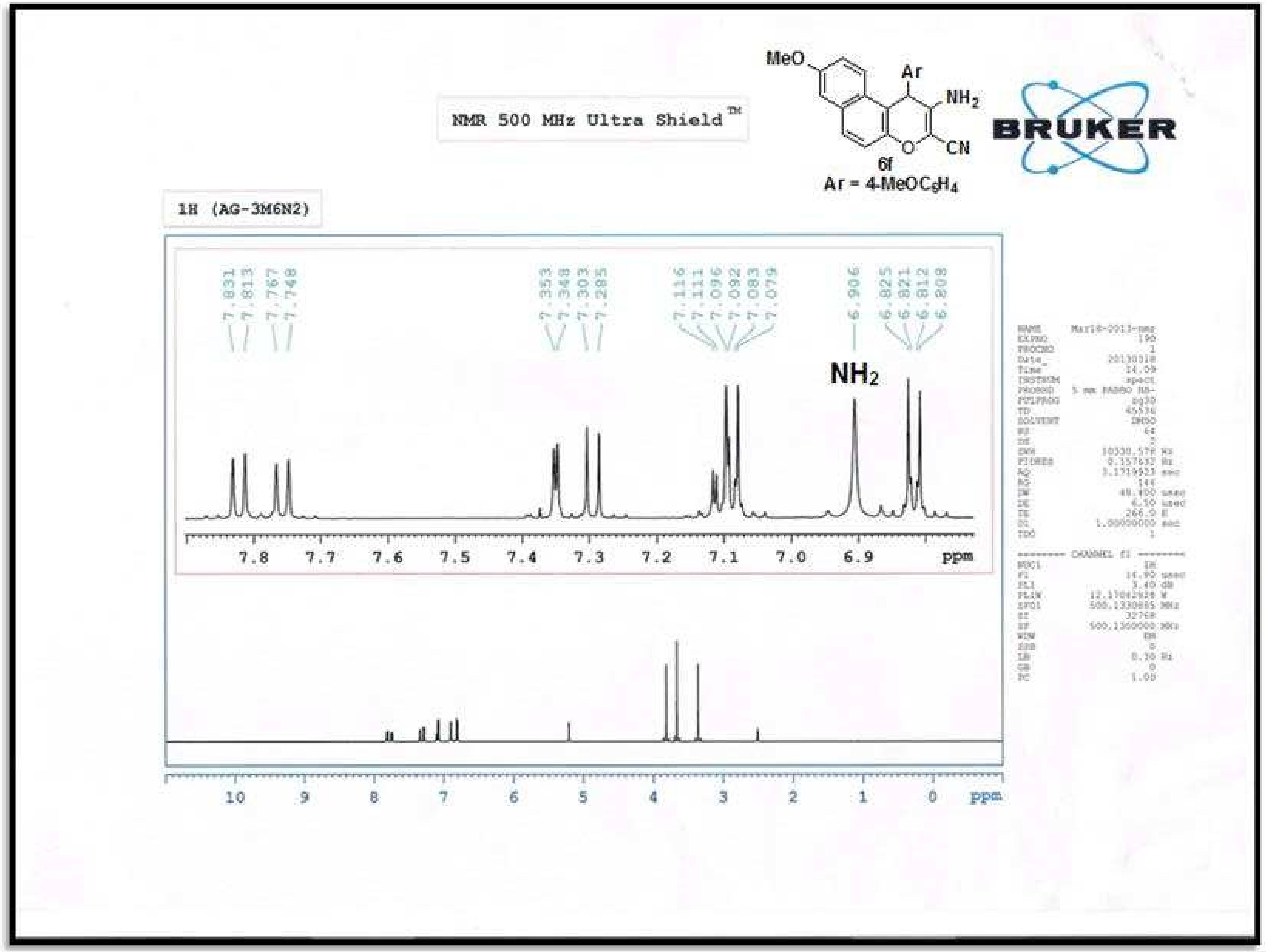
NMR 500 MHz Ultra Shield™
BRUKER
MeO
Ar
NH2
CN
O
6f
Ar = 4-MeOC6H4
1H (AG-3M6N2)
7.831
7.813
7.767
7.748
7.353
7.348
7.303
7.285
7.116
7.111
7.096
7.092
7.083
7.079
6.906
6.825
6.821
6.812
6.808
NH2
ppm

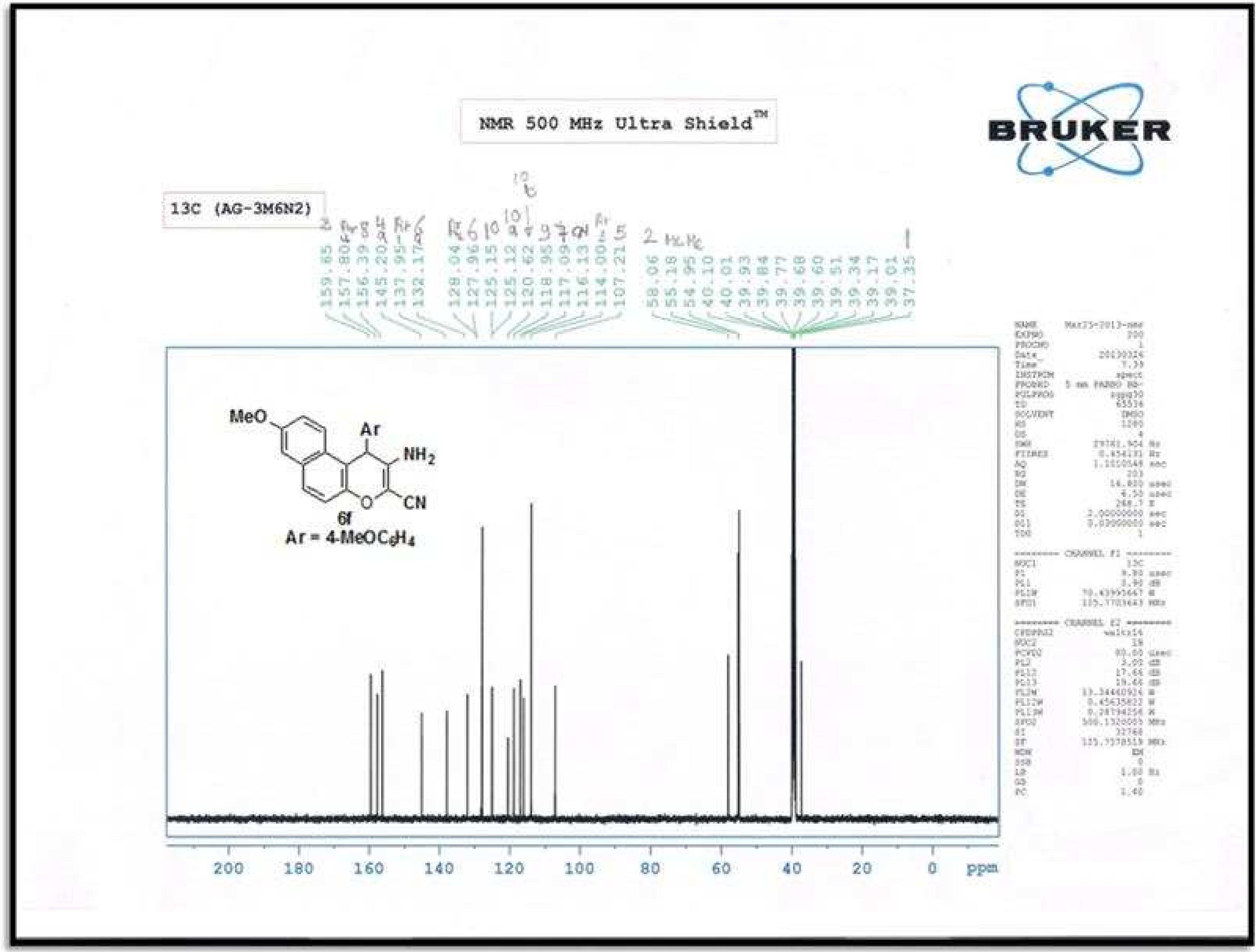
NMR 500 MHz Ultra Shield™
BRUKER
13C (AG-3M6N2)
159.65
157.80
156.39
145.20
137.95
132.17
128.04
127.96
125.15
125.12
120.62
118.95
117.09
116.13
114.00
107.21
58.06
55.18
54.95
40.10
40.01
39.93
39.84
39.77
39.68
39.60
39.51
39.34
39.17
39.01
37.35
MeO
Ar
NH2
CN
O
6f
Ar = 4-MeOC6H4
200
180
160
140
120
100
80
60
40
20
0
ppm

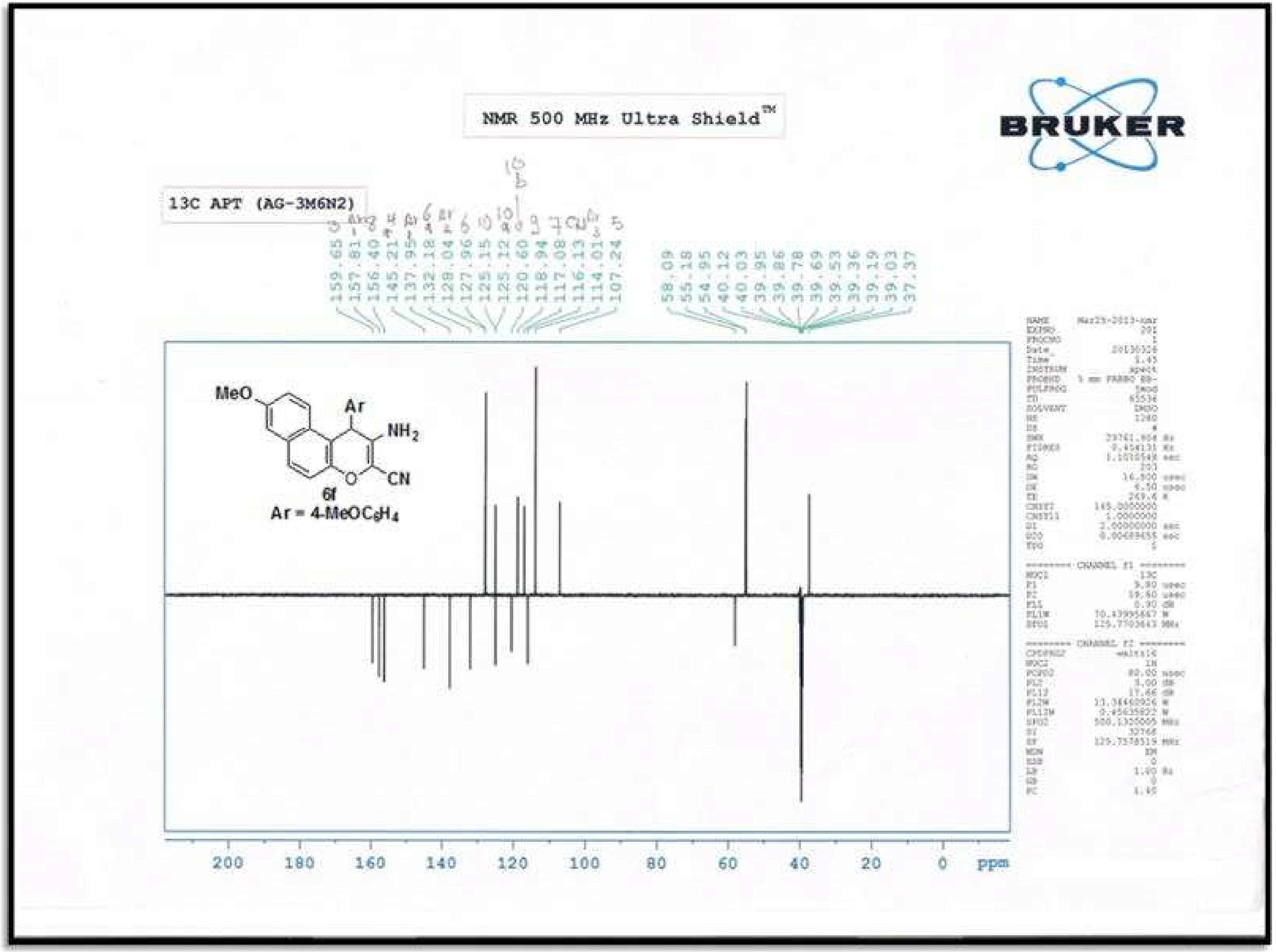
NMR 500 MHz Ultra Shield™
13C APT (AG-3M6N2)
BRUKER
159.65
157.81
156.40
145.21
137.95
132.18
128.04
127.96
125.15
125.12
120.60
118.94
117.08
116.13
114.01
107.24
58.09
55.18
54.95
40.12
40.03
39.95
39.86
39.78
39.69
39.53
39.36
39.19
39.03
37.37
MeO
Ar
NH_2
CN
6f
Ar = 4-MeOC_6H_4
200
180
160
140
120
100
80
60
40
20
0
ppm

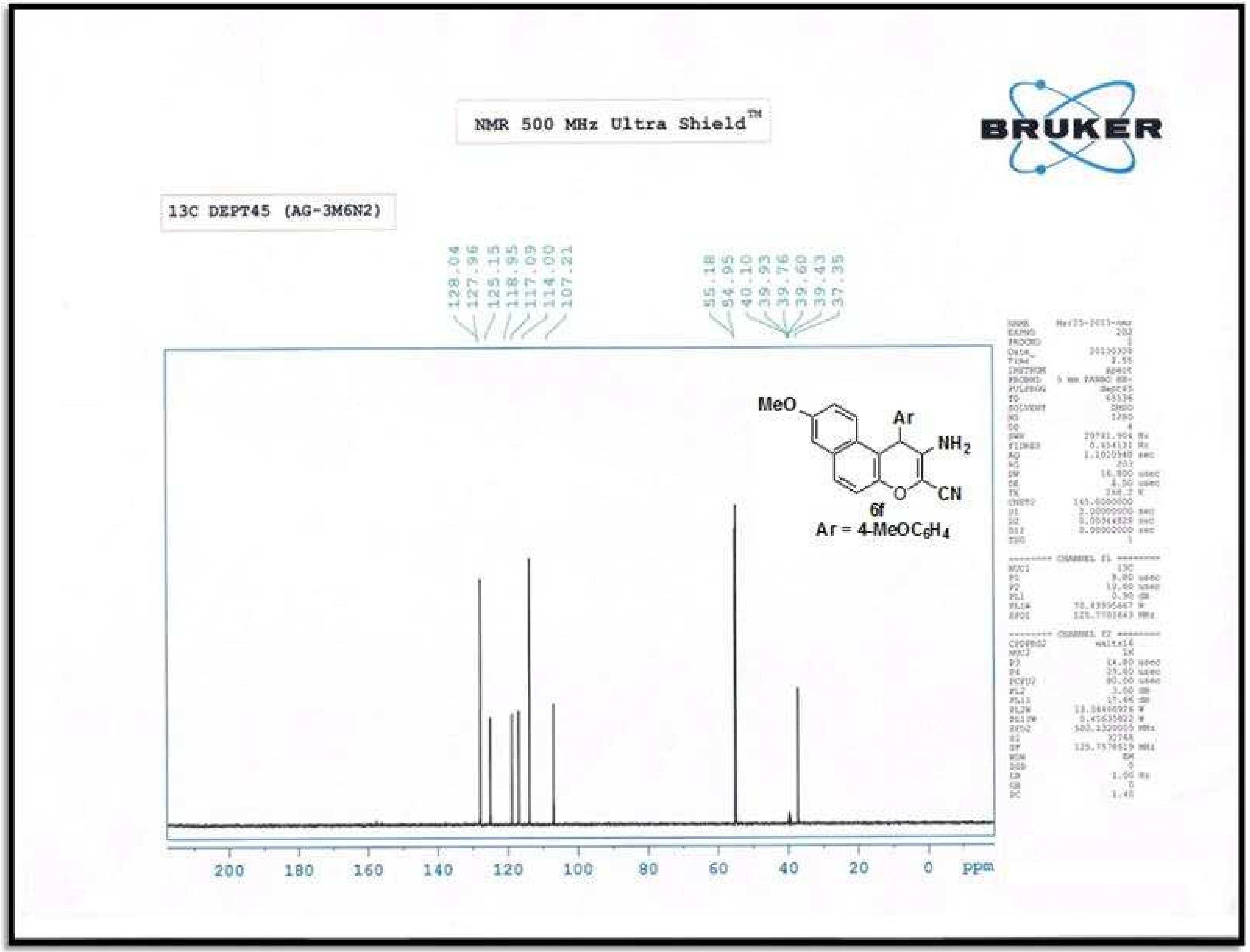
NMR 500 MHz Ultra Shield™
BRUKER
13C DEPT45 (AG-3M6N2)
128.04
127.96
125.15
118.95
117.09
114.00
107.21
55.18
54.95
40.10
39.93
39.76
39.60
39.43
37.35
MeO
Ar
NH2
CN
O
6f
Ar = 4-MeOC6H4
200
180
160
140
120
100
80
60
40
20
0
ppm

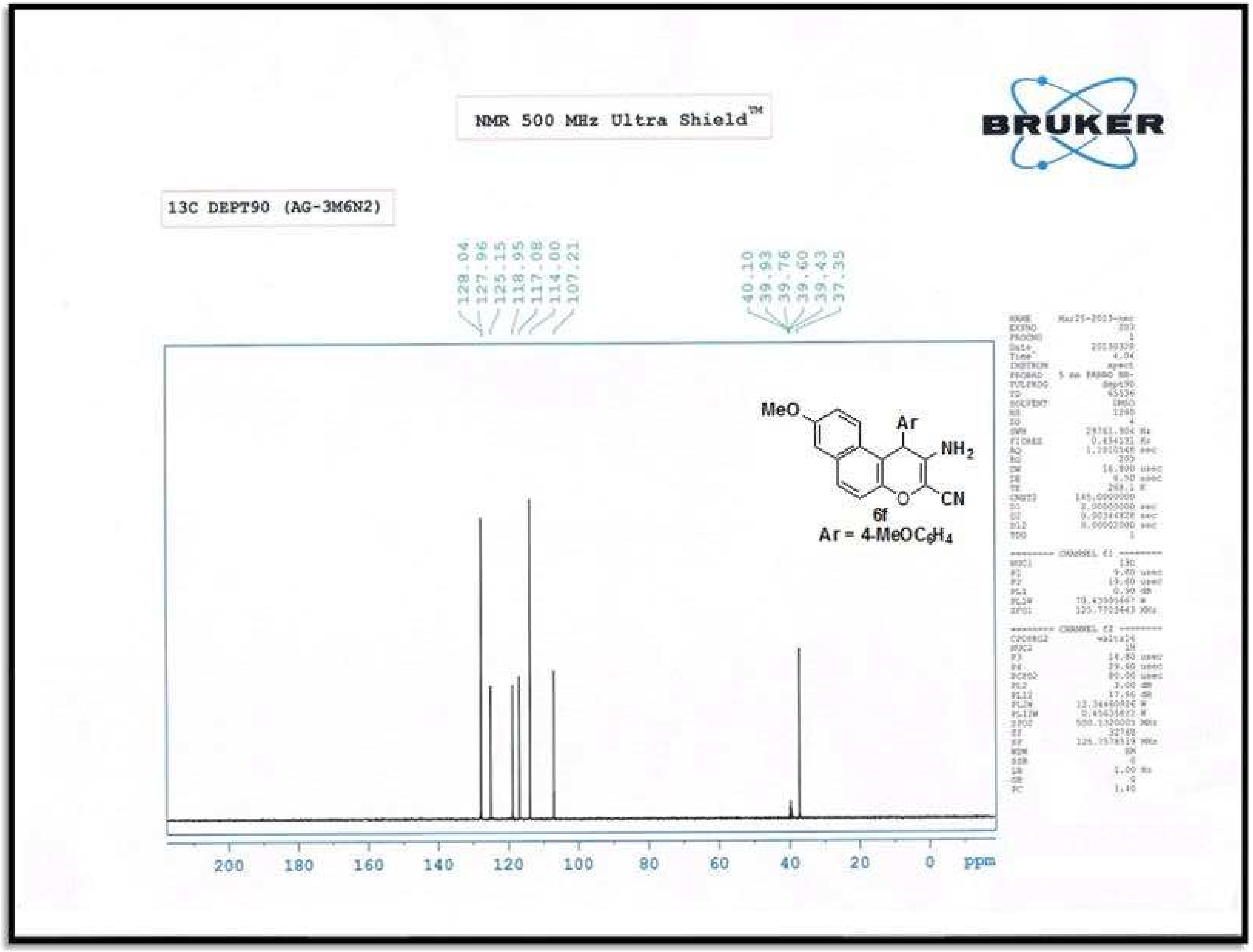
NMR 500 MHz Ultra Shield™
13C DEPT90 (AG-3M6N2)
128.04
127.96
125.15
118.95
117.08
114.00
107.21
40.10
39.93
39.76
39.60
39.43
37.35
MeO
Ar
NH2
CN
O
6f
Ar = 4-MeOC6H4
200
180
160
140
120
100
80
60
40
20
0
ppm

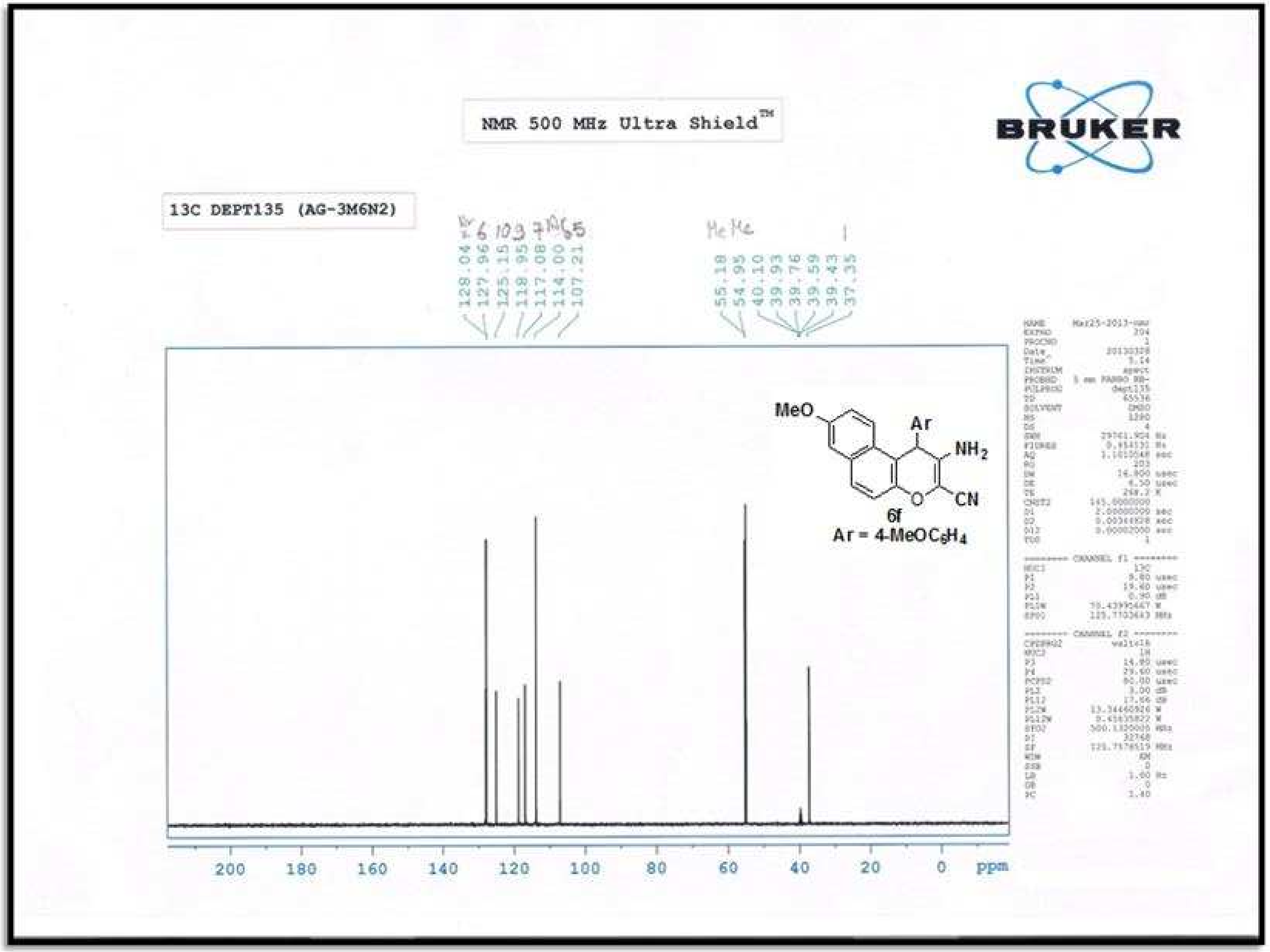
NMR 500 MHz Ultra Shield™
BRUKER
13C DEPT135 (AG-3M6N2)
128.04
127.96
125.15
118.95
117.08
114.00
107.21
55.18
54.95
40.10
39.93
39.76
39.59
39.43
37.35
MeO
Ar
NH2
CN
O
6f
Ar = 4-MeOC6H4
200
180
160
140
120
100
80
60
40
20
0
ppm

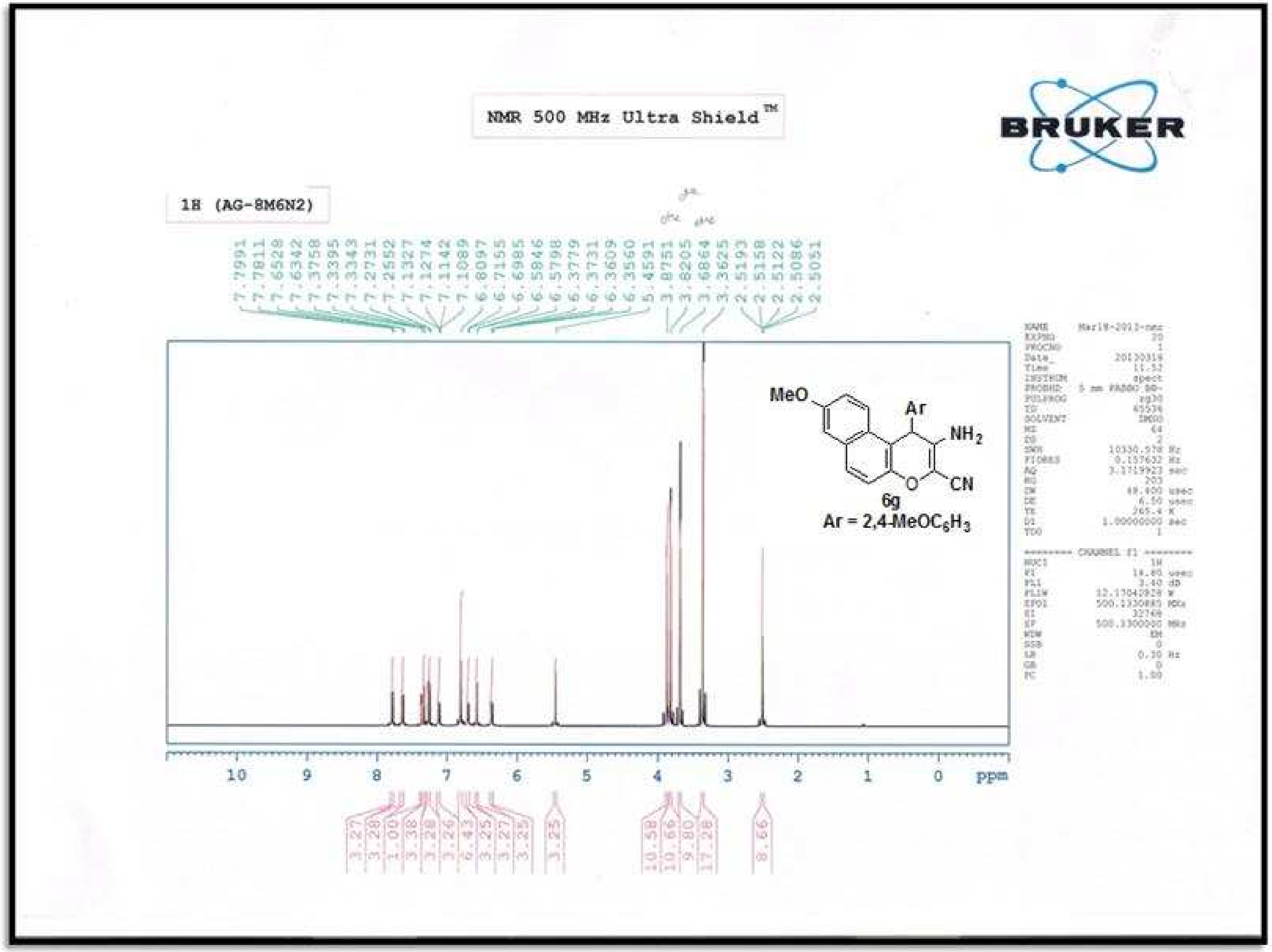
NMR 500 MHz Ultra Shield™
BRUKER
1H (AG-8M6N2)
7.7991
7.7811
7.6528
7.6342
7.3758
7.3395
7.3343
7.2731
7.2552
7.1327
7.1274
7.1142
7.1089
6.8097
6.7155
6.6985
6.5846
6.5798
6.3779
6.3731
6.3609
6.3560
5.4591
3.8751
3.8205
3.6864
3.3625
2.5193
2.5158
2.5122
2.5086
2.5051
MeO
Ar
NH2
CN
O
6g
Ar = 2,4-MeOC6H3
10 9 8 7 6 5 4 3 2 1 0 ppm
3.27
3.28
1.00
3.38
3.28
3.26
6.43
3.25
3.27
3.25
3.25
10.58
10.66
9.80
17.28
8.66

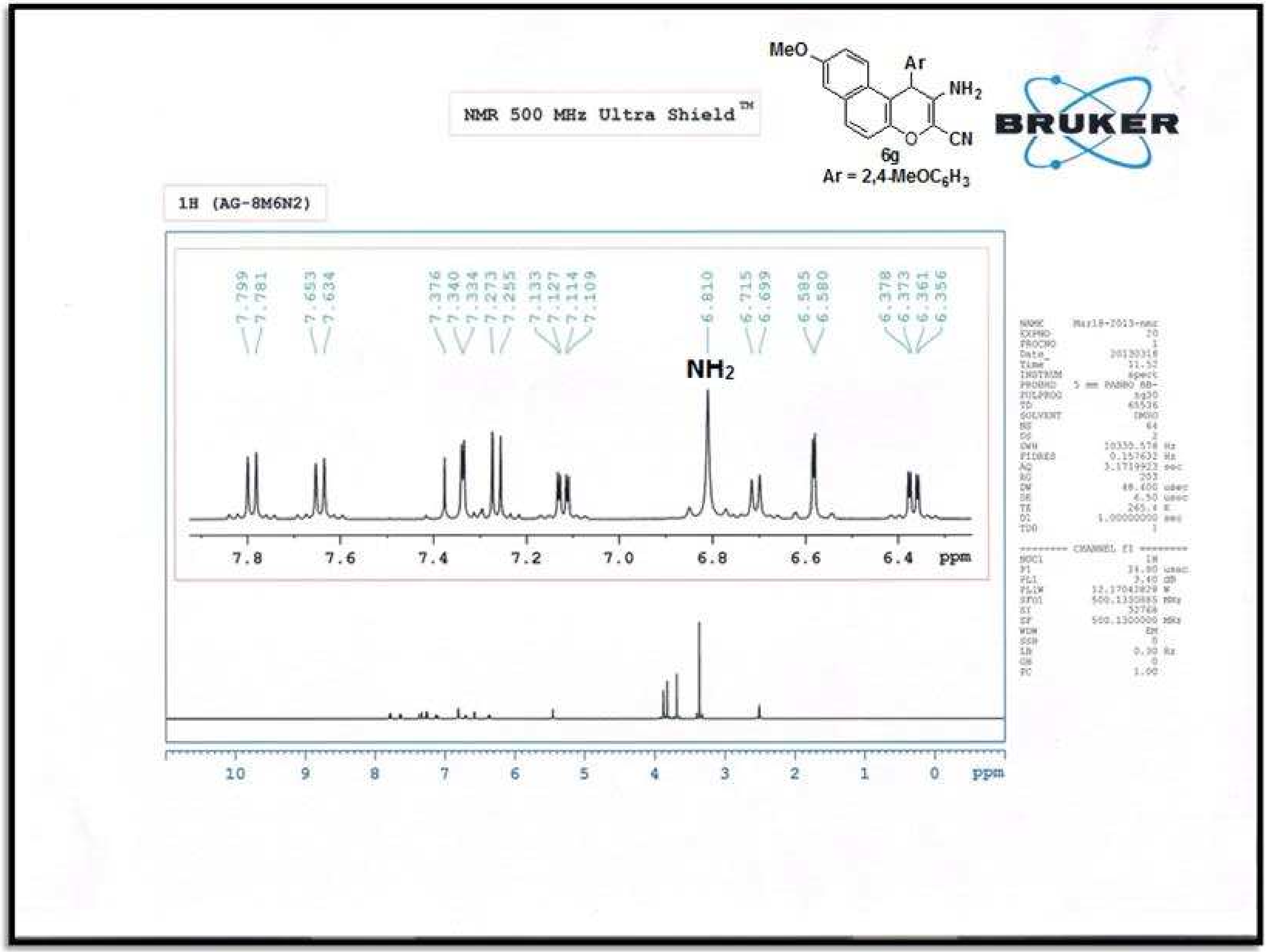
NMR 500 MHz Ultra Shield™
MeO
Ar
NH2
CN
O
6g
Ar = 2,4-MeOC6H3
BRUKER
1H (AG-8M6N2)
7.799
7.781
7.653
7.634
7.376
7.340
7.334
7.273
7.255
7.133
7.127
7.114
7.109
6.810
6.715
6.699
6.585
6.580
6.378
6.373
6.361
6.356
NH2
7.8
7.6
7.4
7.2
7.0
6.8
6.6
6.4
ppm
10
9
8
7
6
5
4
3
2
1
0
ppm

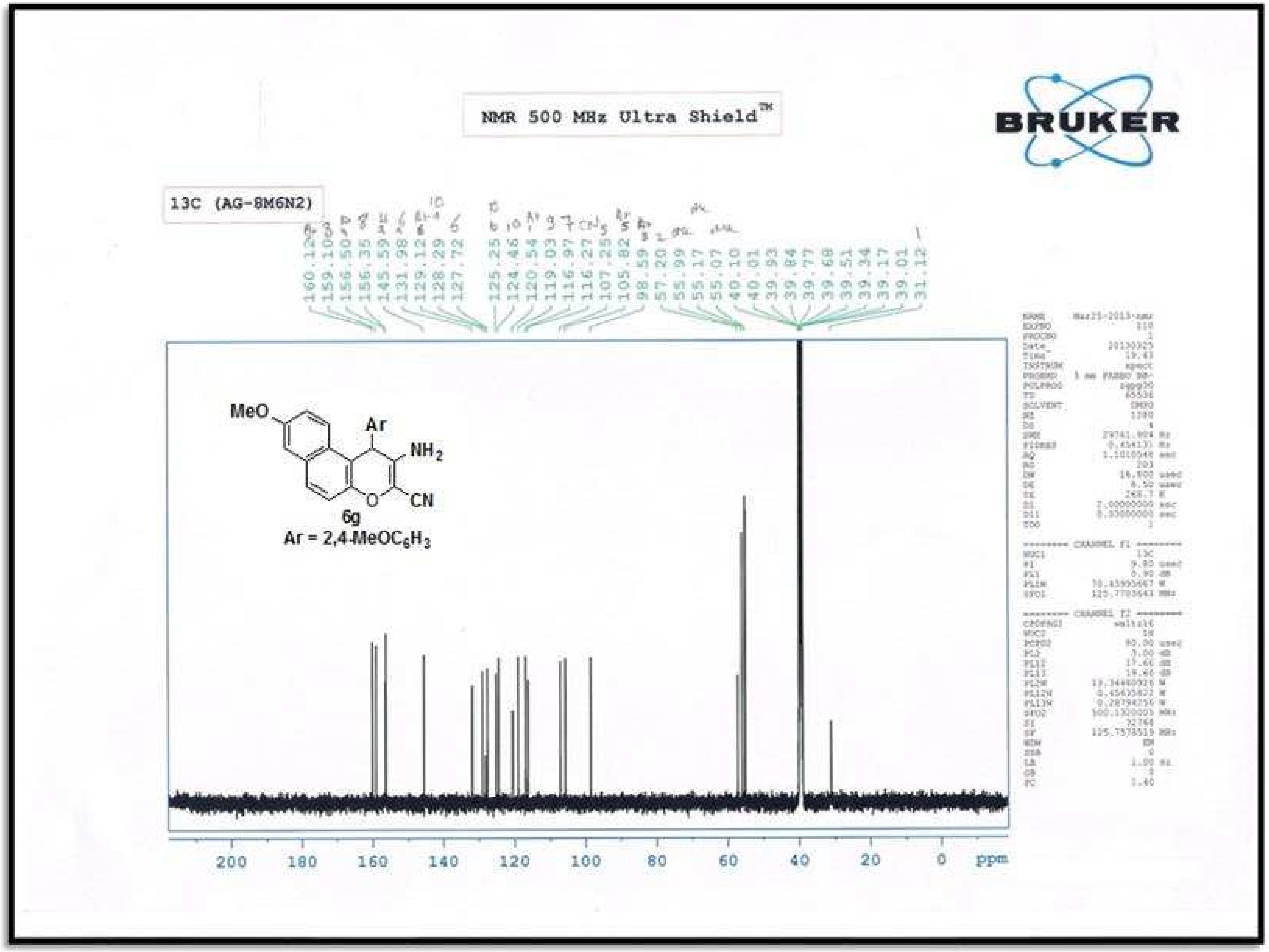
NMR 500 MHz Ultra Shield™
BRUKER
13C (AG-8M6N2)
160.12
159.10
156.50
156.35
145.59
131.98
129.12
128.29
127.72
125.25
124.46
120.54
119.03
116.97
116.27
107.25
105.82
98.59
57.20
55.99
55.17
55.07
40.10
40.01
39.93
39.84
39.77
39.68
39.51
39.34
39.17
39.01
31.12
MeO
Ar
NH2
CN
O
6g
Ar = 2,4-MeOC6H3
200
180
160
140
120
100
80
60
40
20
0
ppm

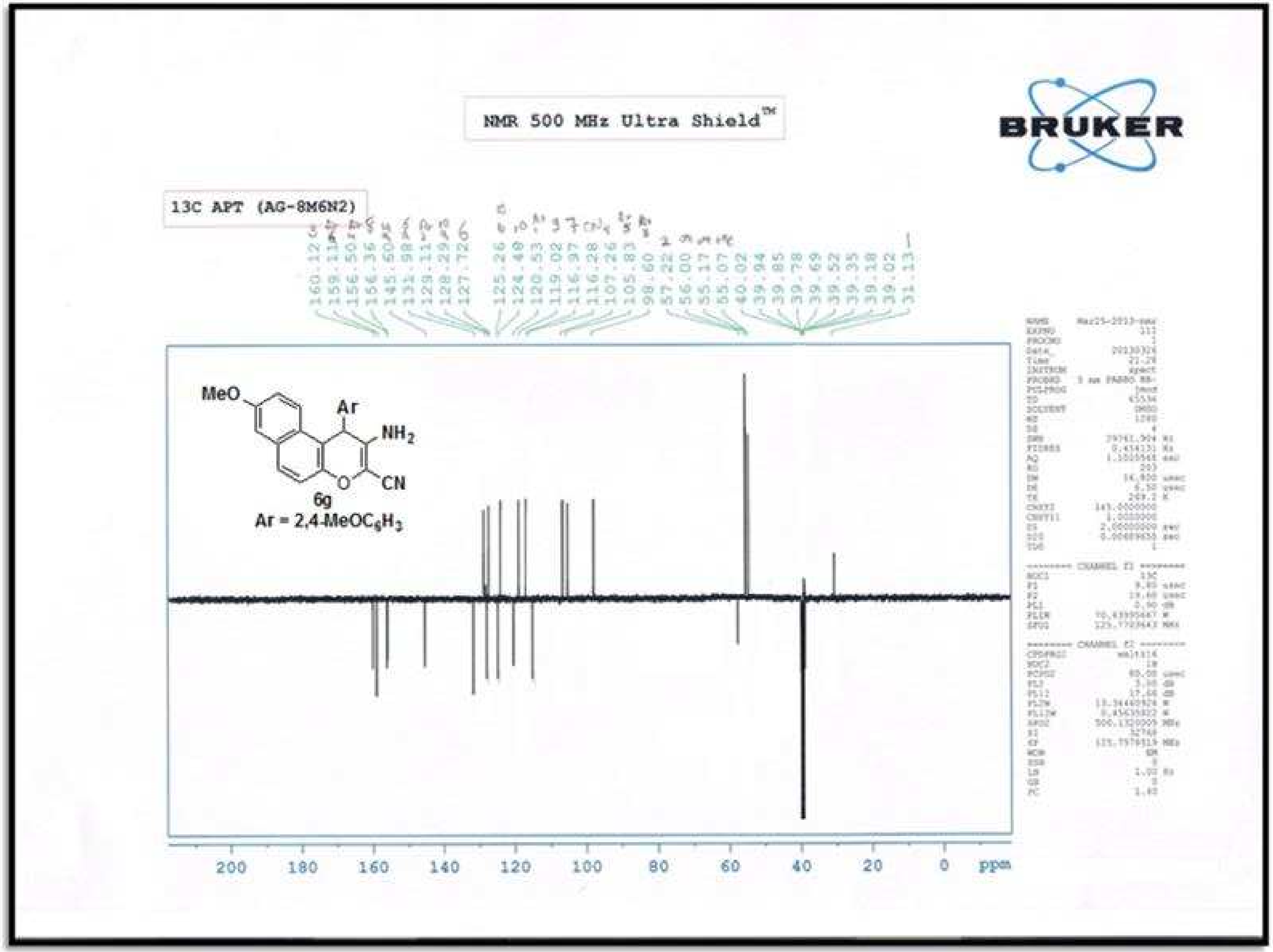
NMR 500 MHz Ultra Shield™
13C APT (AG-8M6N2)
160.12
159.11
156.50
156.36
145.60
131.98
129.11
128.29
127.72
125.26
124.48
120.53
119.02
116.97
116.28
107.26
105.83
98.60
57.22
56.00
55.17
55.07
40.02
39.94
39.85
39.78
39.69
39.52
39.35
39.18
39.02
31.13
MeO
Ar
NH2
CN
O
6g
Ar = 2,4-MeOC6H3
200
180
160
140
120
100
80
60
40
20
0
ppm

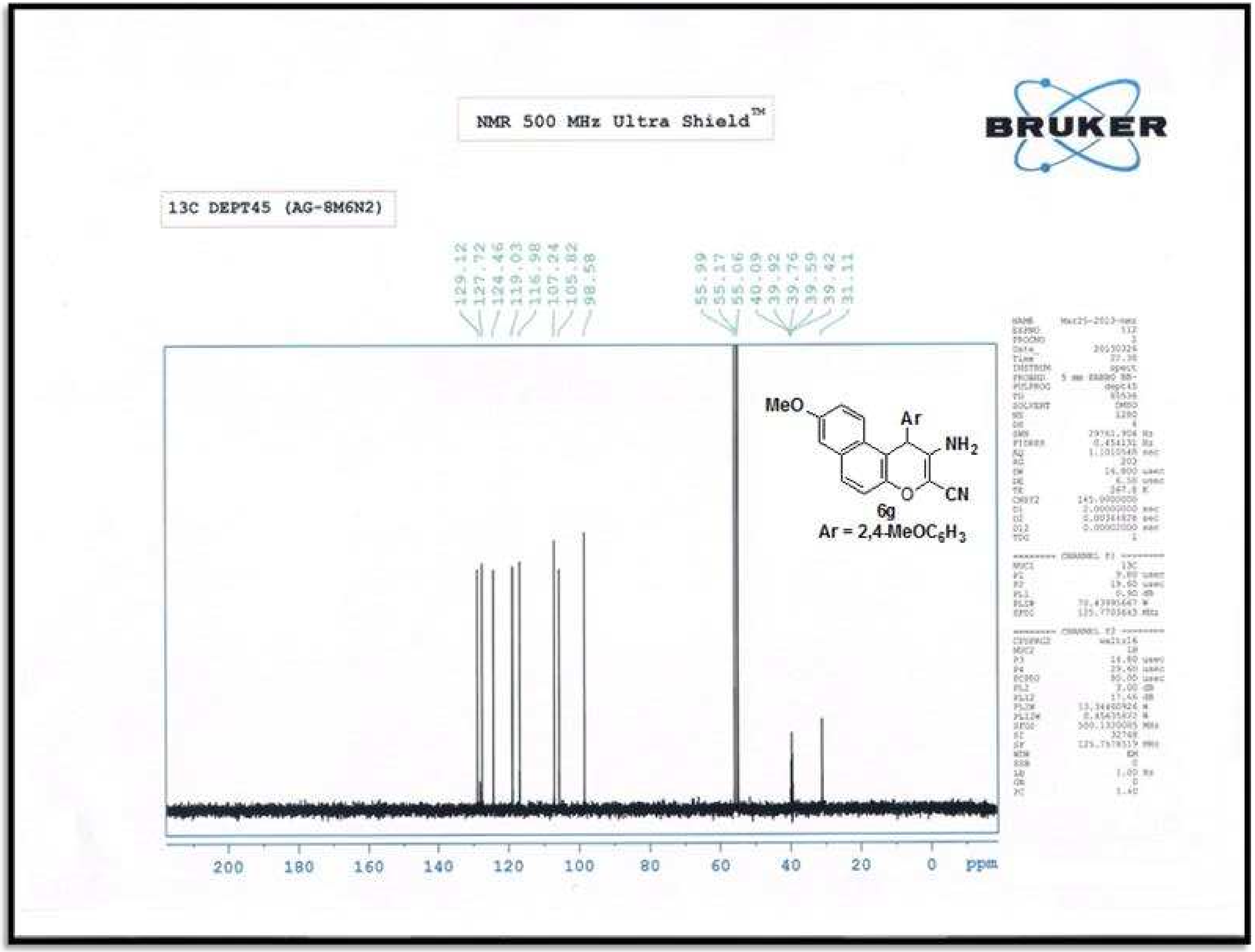
NMR 500 MHz Ultra Shield™
BRUKER
13C DEPT45 (AG-8M6N2)
129.12
127.72
124.46
119.03
116.98
107.24
105.82
98.58
55.99
55.17
55.06
40.09
39.92
39.76
39.59
39.42
31.11
MeO
Ar
NH2
CN
O
6g
Ar = 2,4-MeOC6H3
200
180
160
140
120
100
80
60
40
20
0
ppm

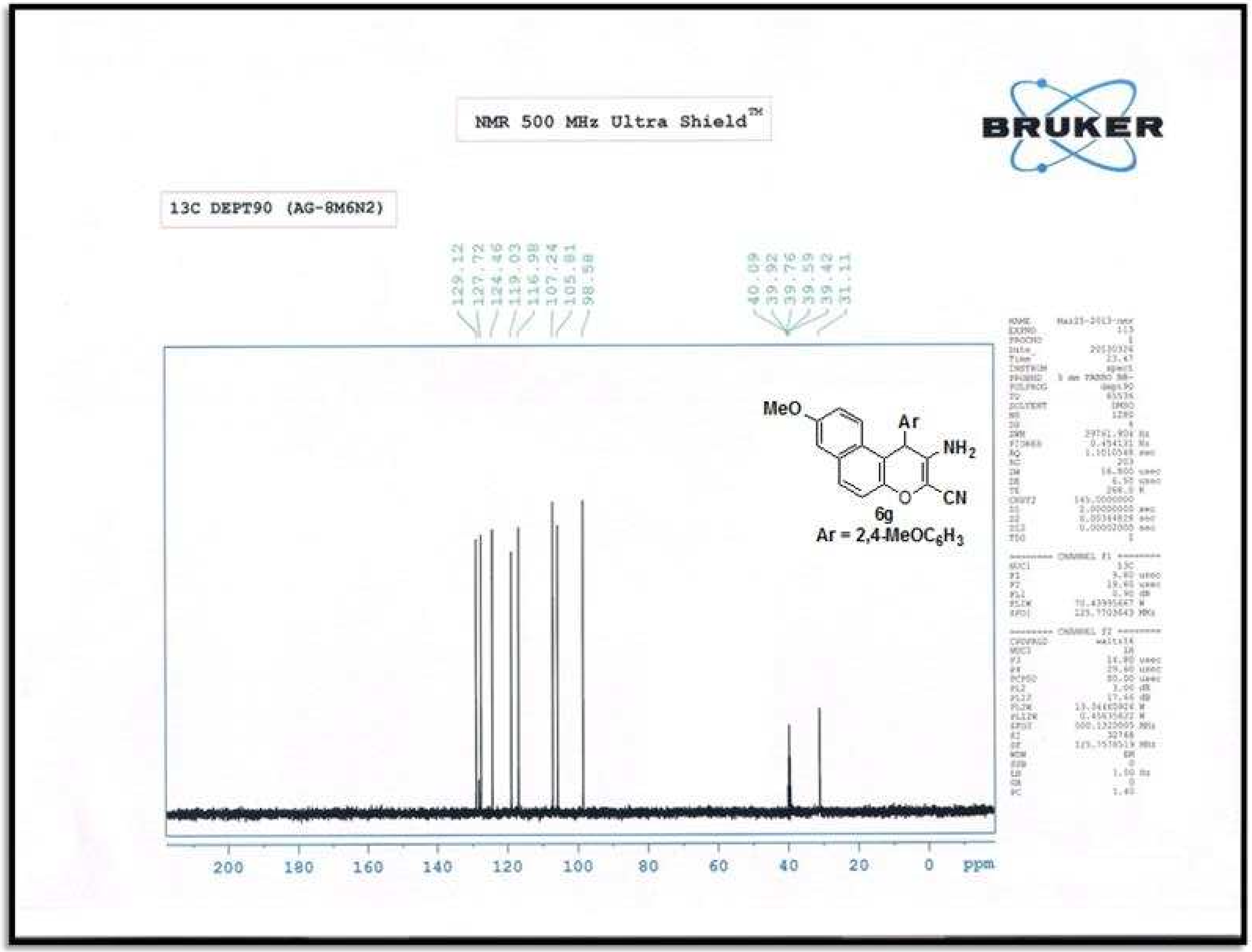

BRUKER
NMR 500 MHz Ultra Shield™
13C DEPT90 (AG-8M6N2)
129.12
127.72
124.46
119.03
116.98
107.24
105.81
98.58
40.09
39.92
39.76
39.59
39.42
31.11
MeO
Ar
NH2
CN
O
6g
Ar = 2,4-MeOC6H3
200
180
160
140
120
100
80
60
40
20
0
ppm

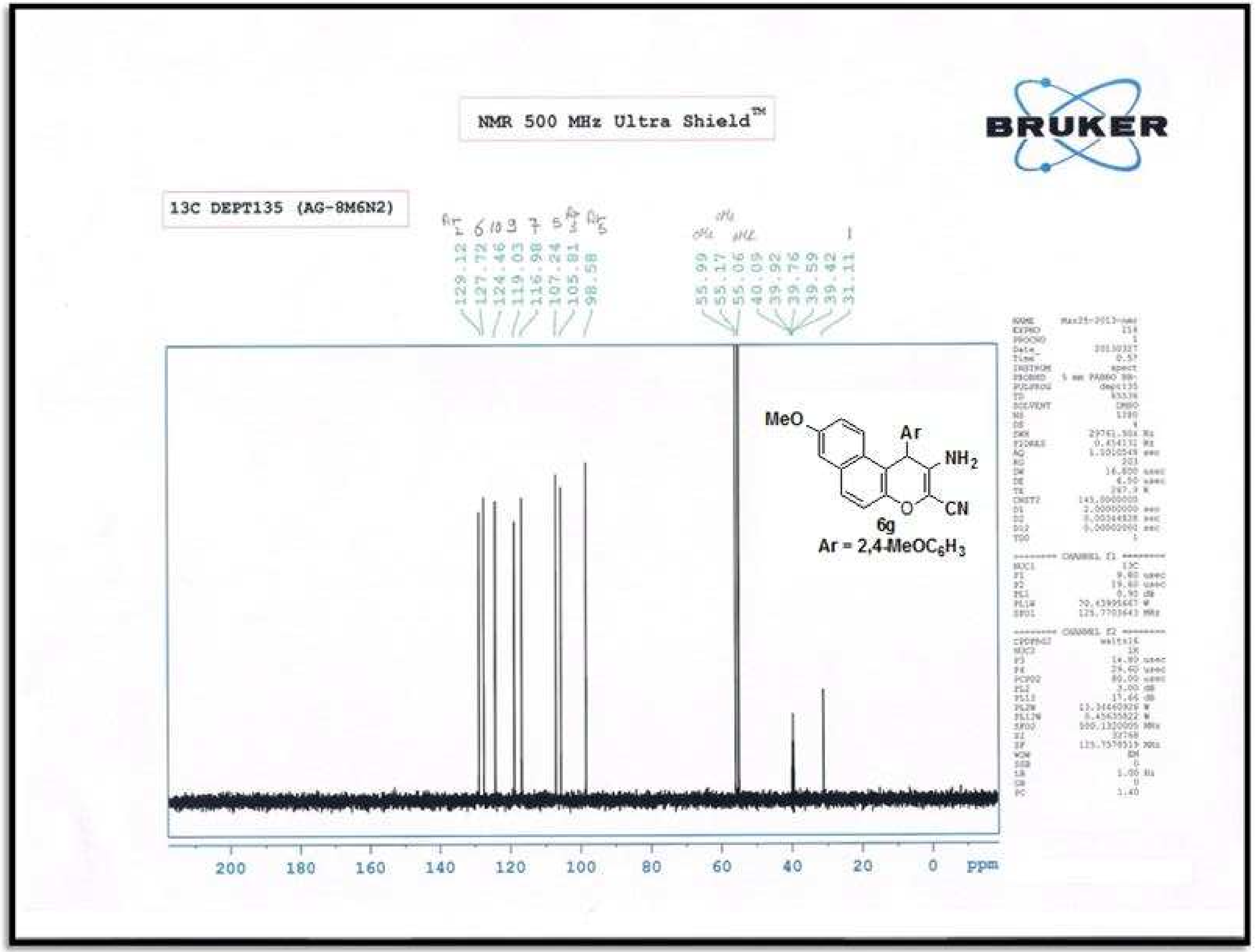
NMR 500 MHz Ultra Shield™
BRUKER
13C DEPT135 (AG-8M6N2)
129.12
127.72
124.46
119.03
116.98
107.24
105.81
98.58
55.99
55.17
55.06
40.09
39.92
39.76
39.59
39.42
31.11
MeO
Ar
NH2
CN
O
6g
Ar = 2,4-MeOC6H3
200
180
160
140
120
100
80
60
40
20
0
ppm

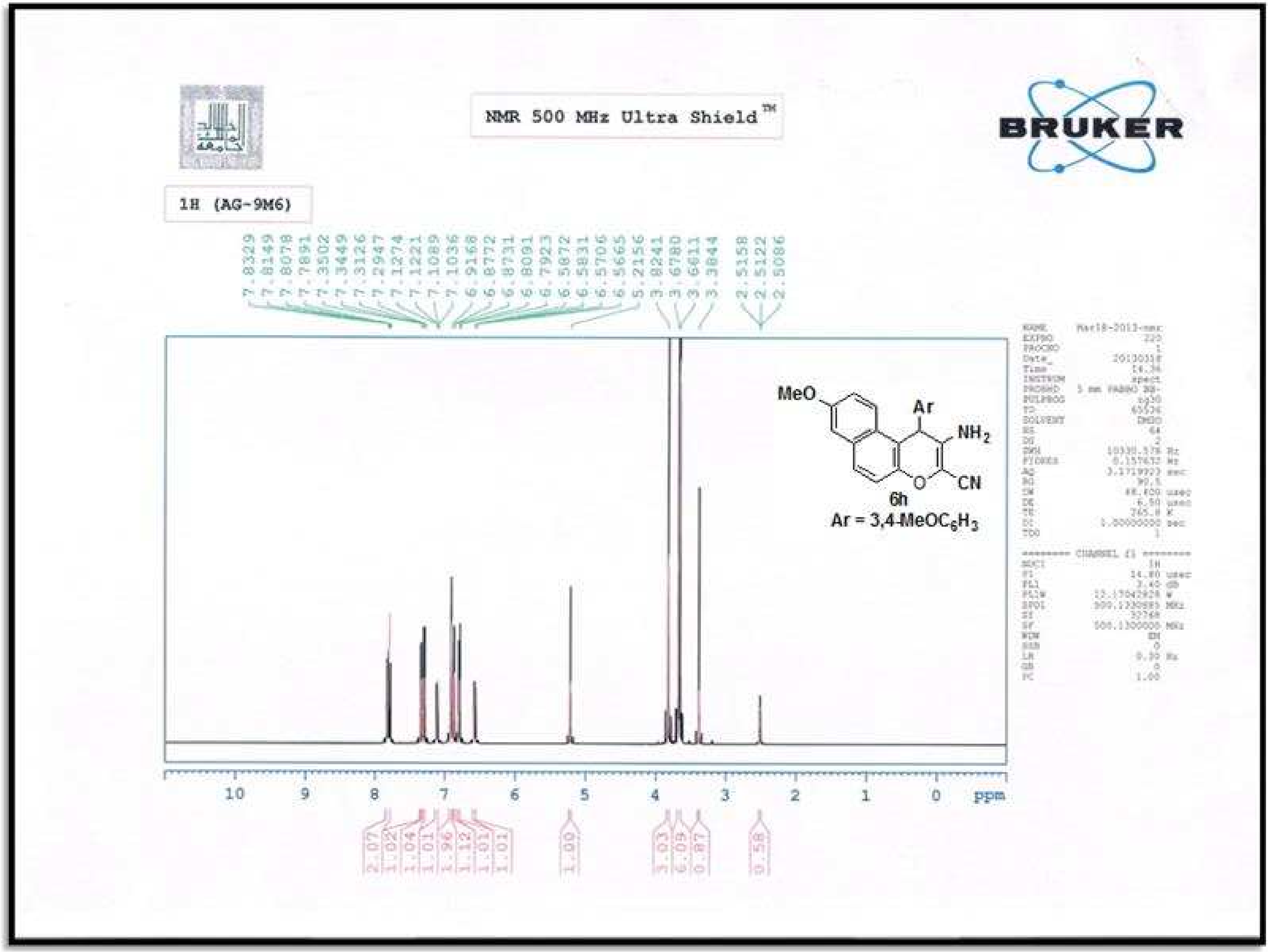
NMR 500 MHz Ultra Shield™
BRUKER
1H (AG-9M6)
7.8329
7.8149
7.8078
7.7891
7.3502
7.3449
7.3126
7.2947
7.1274
7.1221
7.1089
7.1036
6.9168
6.8772
6.8731
6.8091
6.7923
6.5872
6.5831
6.5706
6.5665
5.2156
3.8241
3.6780
3.6611
3.3844
2.5158
2.5122
2.5086
MeO
Ar
NH2
CN
6h
Ar = 3,4-MeOC6H3
ppm
2.07
1.02
1.04
1.01
1.96
1.12
1.01
1.01
1.00
3.03
6.09
0.87
0.58

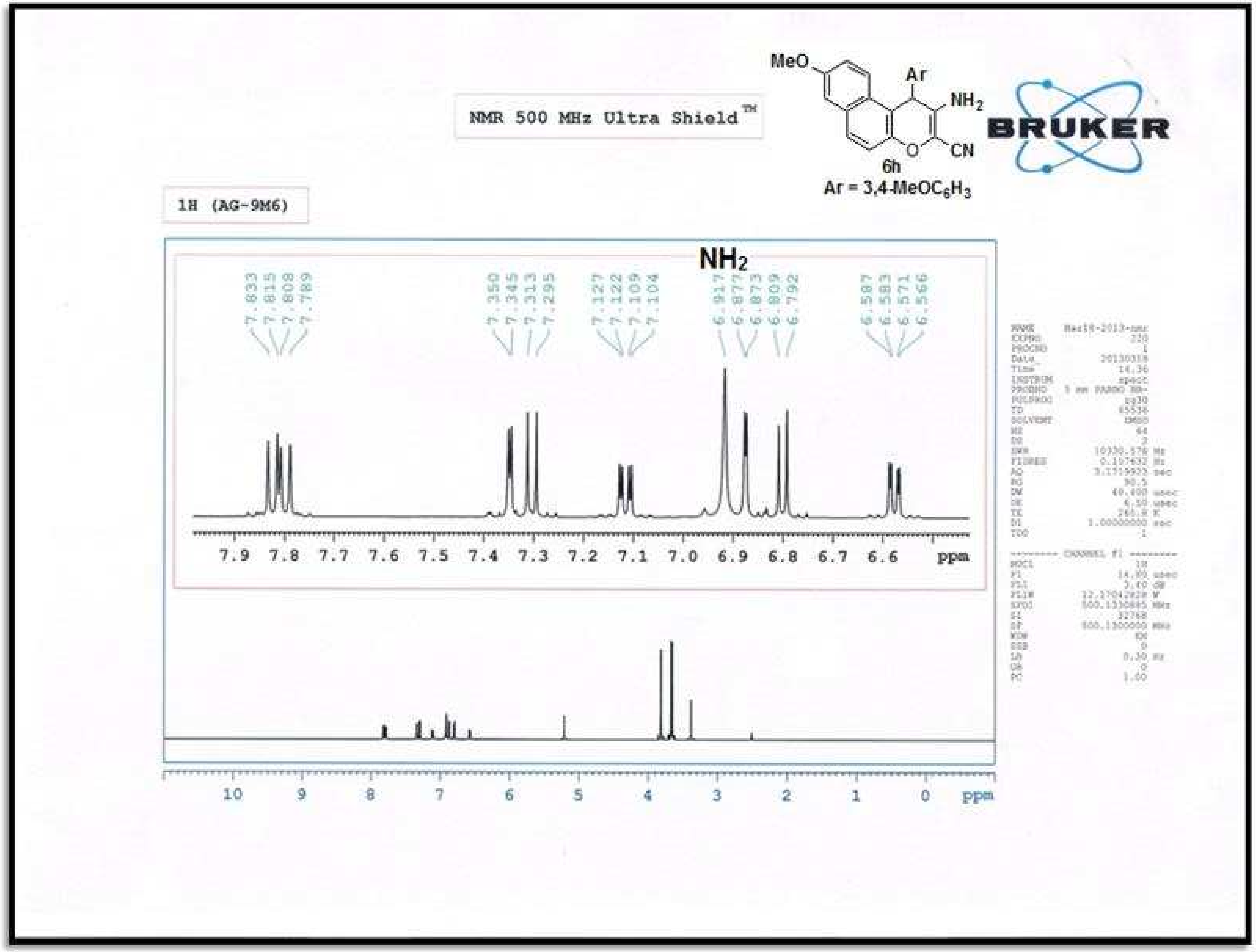
NMR 500 MHz Ultra Shield™
MeO
Ar
NH2
CN
O
6h
Ar = 3,4-MeOC6H3
BRUKER
1H (AG-9M6)
NH2
7.833
7.815
7.808
7.789
7.350
7.345
7.313
7.295
7.127
7.122
7.109
7.104
6.917
6.877
6.873
6.809
6.792
6.587
6.583
6.571
6.566
7.9 7.8 7.7 7.6 7.5 7.4 7.3 7.2 7.1 7.0 6.9 6.8 6.7 6.6 ppm
10 9 8 7 6 5 4 3 2 1 0 ppm

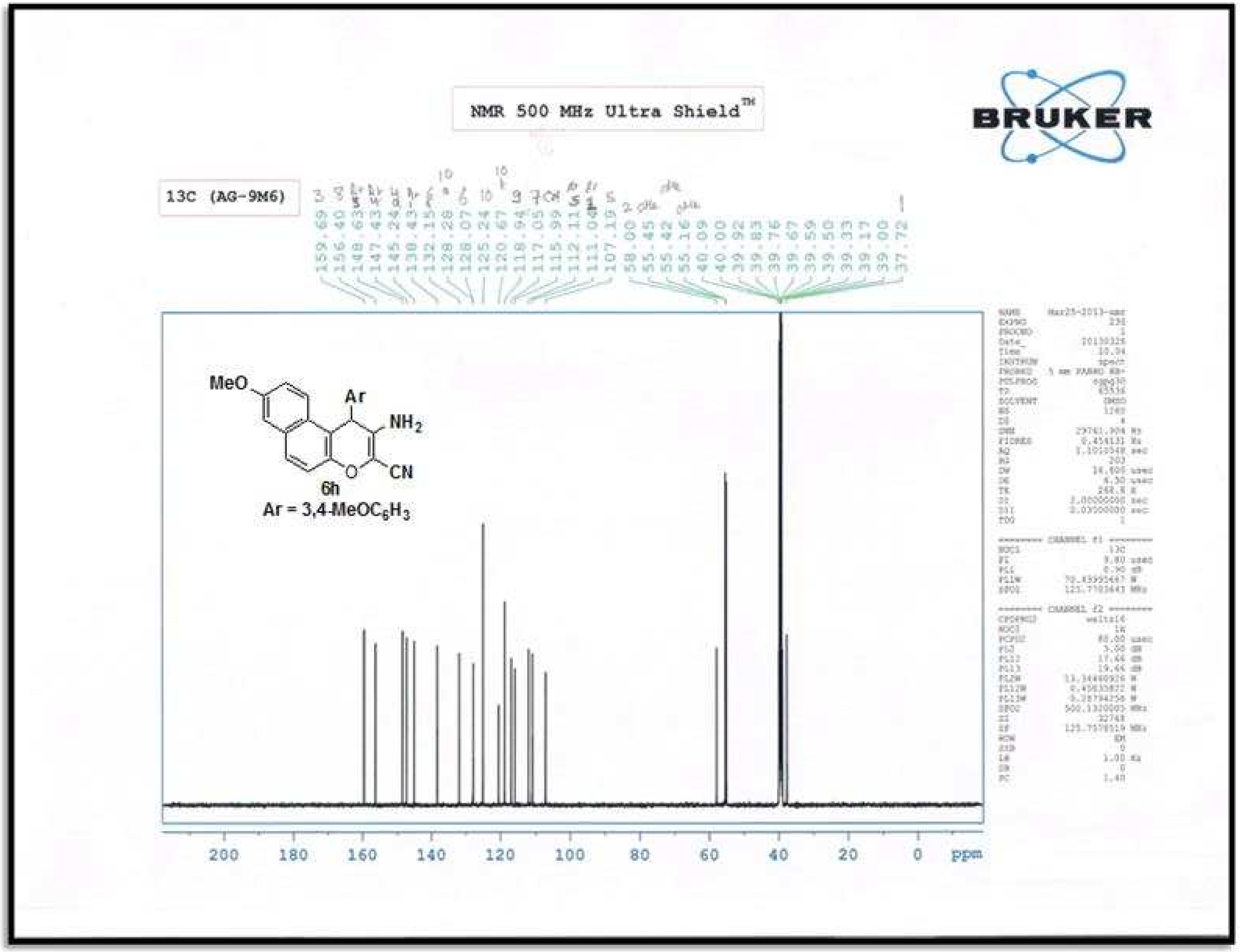

NMR 500 MHz Ultra Shield™
BRUKER
13C (AG-9M6)
159.69
156.40
148.63
147.43
145.24
138.43
132.15
128.28
128.07
125.24
120.67
118.94
117.05
115.99
112.11
111.04
107.19
58.00
55.45
55.42
55.16
40.09
40.00
39.92
39.83
39.76
39.67
39.59
39.50
39.33
39.17
39.00
37.72
MeO
Ar
NH2
CN
O
6h
Ar = 3,4-MeOC6H3
200
180
160
140
120
100
80
60
40
20
0
ppm

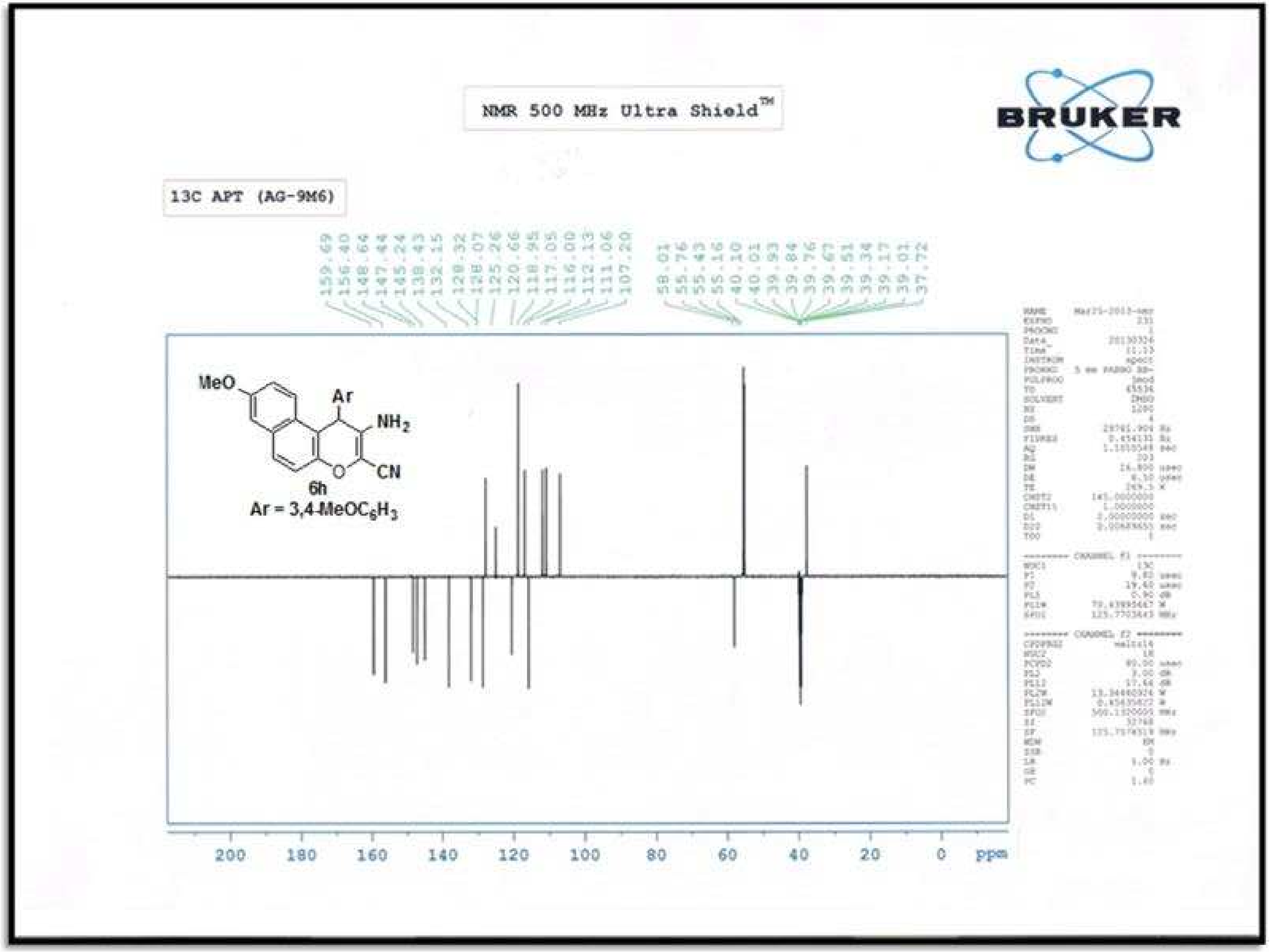
NMR 500 MHz Ultra Shield™
13C APT (AG-9M6)
159.69
156.40
148.64
147.44
145.24
138.43
132.15
128.32
128.07
125.26
120.66
118.95
117.05
116.00
112.13
111.06
107.20
58.01
55.76
55.43
55.16
40.10
40.01
39.93
39.84
39.76
39.67
39.51
39.34
39.17
39.01
37.72
MeO
Ar
NH2
CN
O
6h
Ar = 3,4-MeOC6H3
200
180
160
140
120
100
80
60
40
20
0
ppm

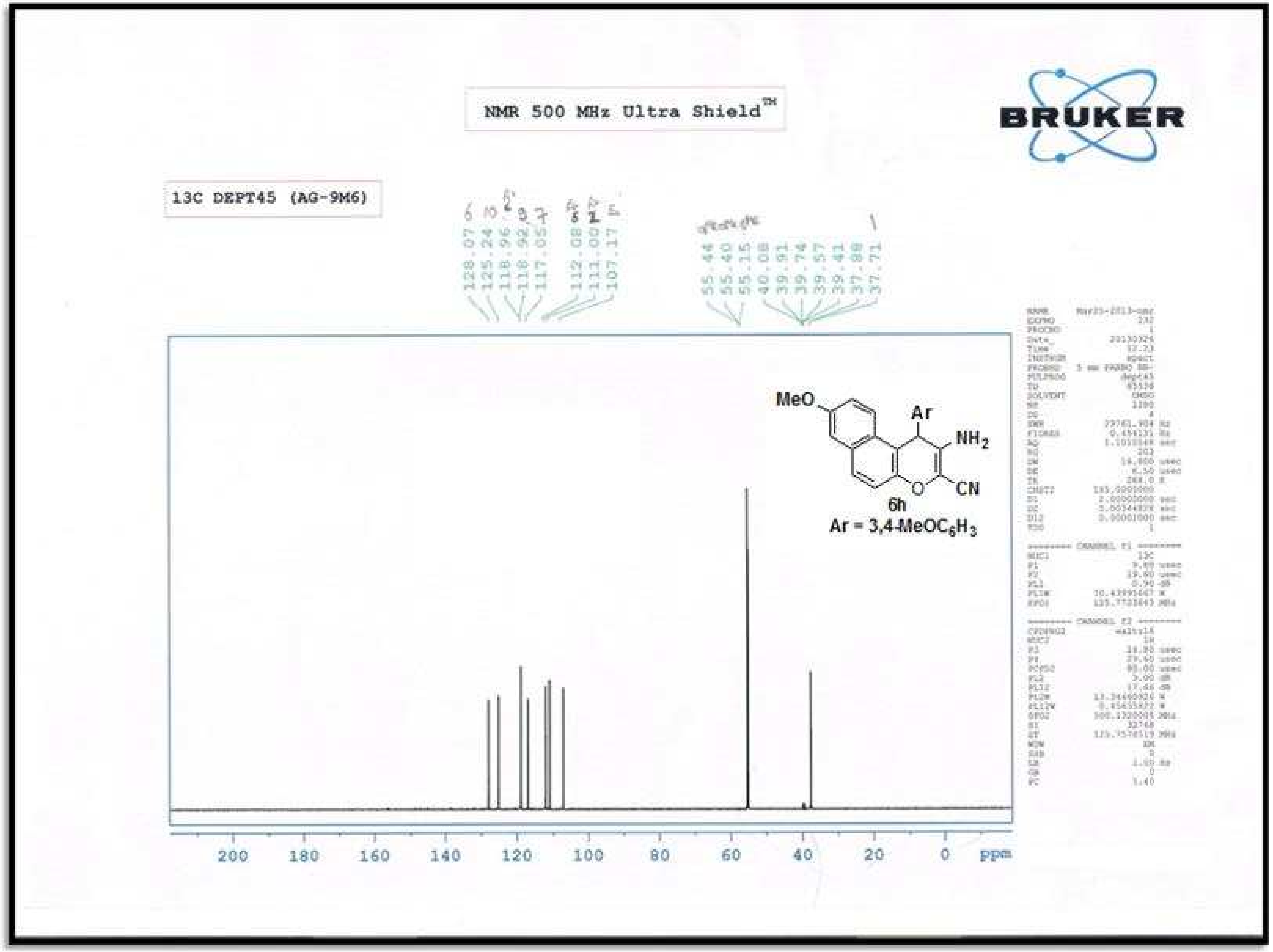
NMR 500 MHz Ultra Shield™
BRUKER
13C DEPT45 (AG-9M6)
128.07
125.24
118.96
118.92
117.05
112.08
111.00
107.17
55.44
55.40
55.15
40.08
39.91
39.74
39.57
39.41
37.88
37.71
MeO
Ar
NH2
CN
O
6h
Ar = 3,4-MeOC6H3
200
180
160
140
120
100
80
60
40
20
0
ppm

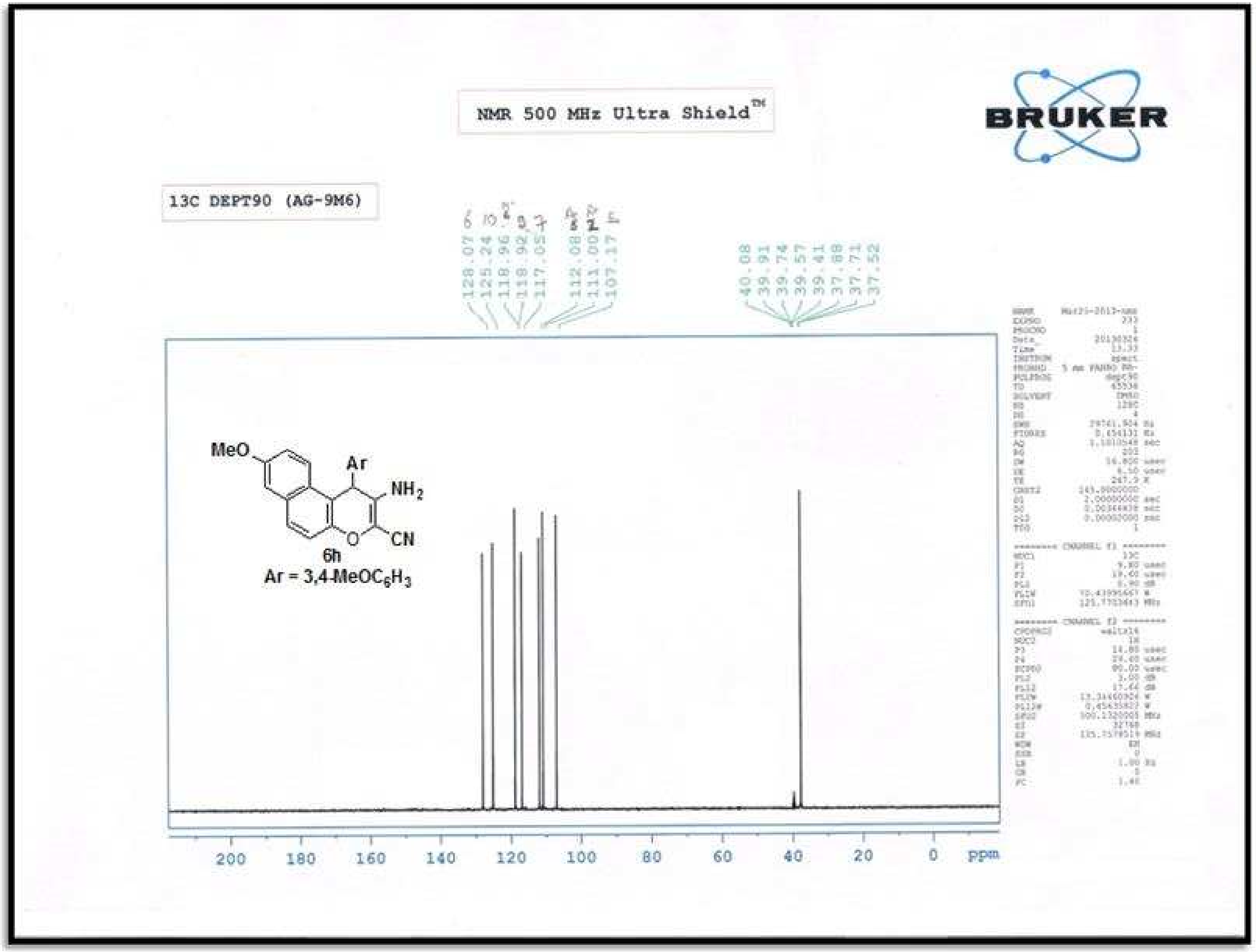
NMR 500 MHz Ultra Shield™
BRUKER
13C DEPT90 (AG-9M6)
128.07
125.24
118.96
118.92
117.05
112.08
111.00
107.17
40.08
39.91
39.74
39.57
39.41
37.88
37.71
37.52
MeO
Ar
NH2
CN
O
6h
Ar = 3,4-MeOC6H3
200 180 160 140 120 100 80 60 40 20 0 ppm

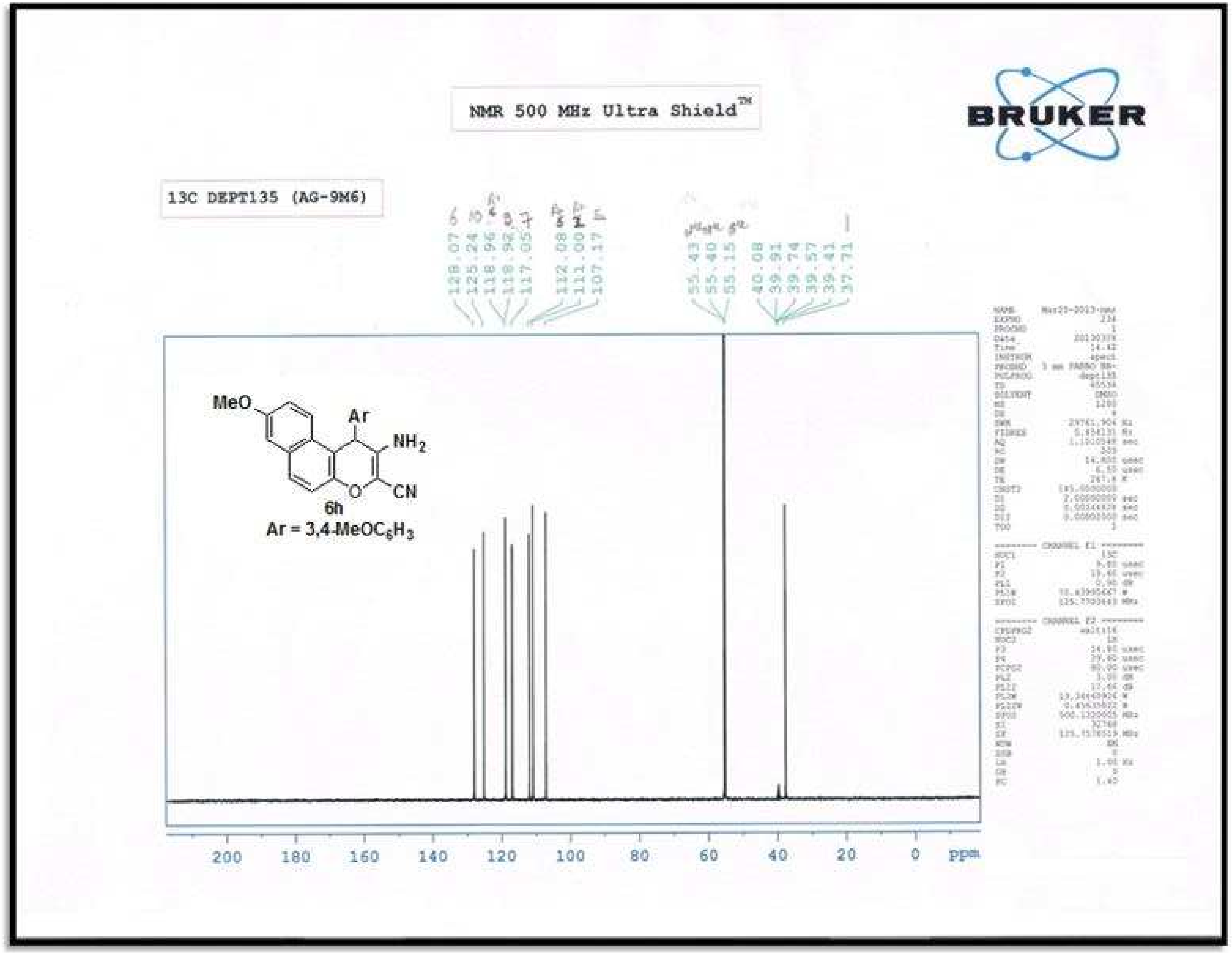
NMR 500 MHz Ultra Shield™
BRUKER
13C DEPT135 (AG-9M6)
128.07
125.24
118.96
118.92
117.05
112.08
111.00
107.17
55.43
55.40
55.15
40.08
39.91
39.74
39.57
39.41
37.71
MeO
Ar
NH2
CN
O
6h
Ar = 3,4-MeOC6H3
200
180
160
140
120
100
80
60
40
20
0
ppm

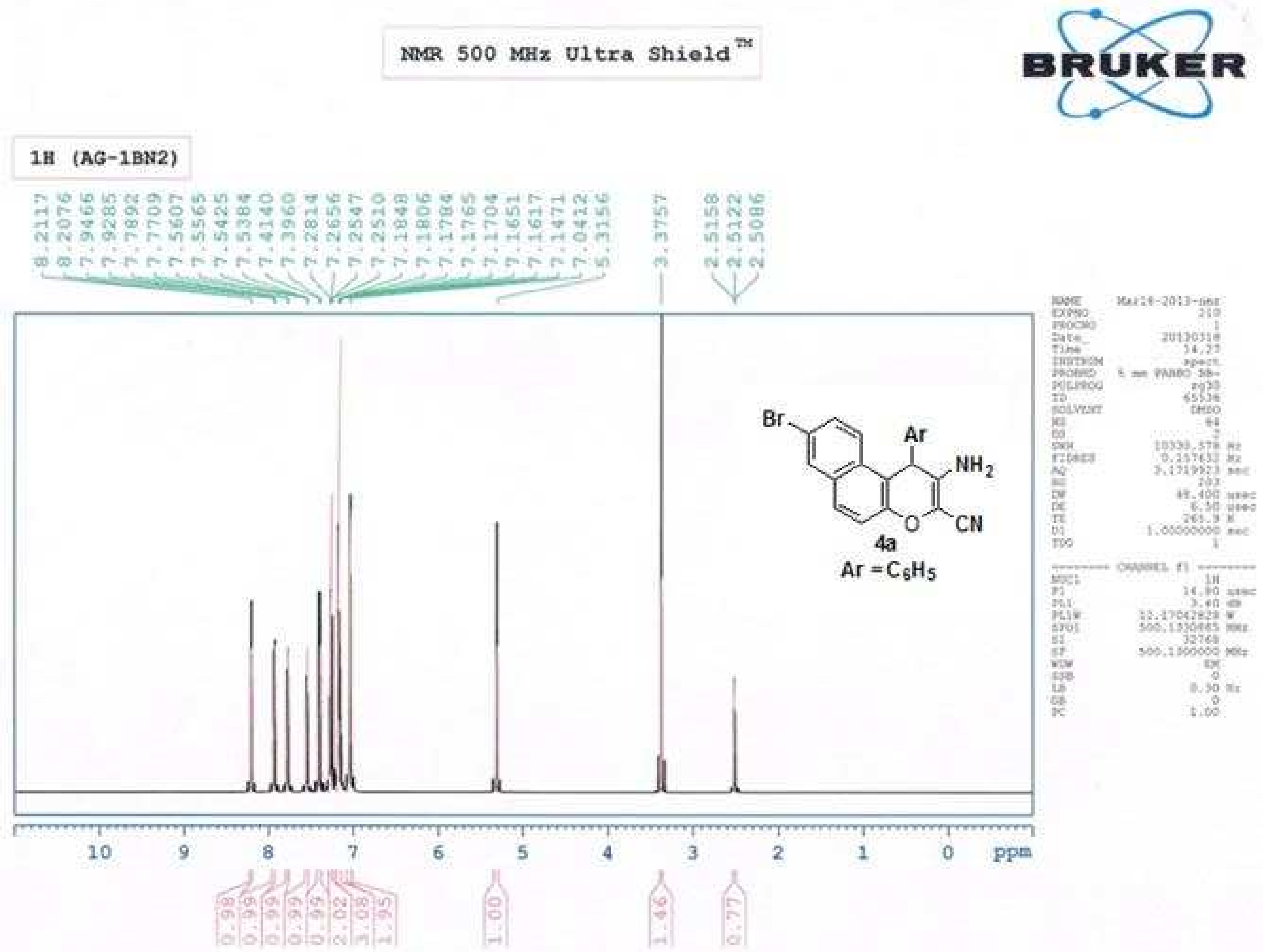
NMR 500 MHz Ultra Shield™
BRUKER
1H (AG-1BN2)
8.2117
8.2076
7.9466
7.9285
7.7892
7.7709
7.5607
7.5565
7.5425
7.5384
7.4140
7.3960
7.2814
7.2656
7.2547
7.2510
7.1848
7.1806
7.1784
7.1765
7.1704
7.1651
7.1617
7.1471
7.0412
5.3156
3.3757
2.5158
2.5122
2.5086
Br
Ar
NH2
O
CN
4a
Ar = C6H5
10
9
8
7
6
5
4
3
2
1
0
ppm
0.98
0.99
0.99
0.99
0.99
2.02
3.08
1.95
1.00
1.46
0.77

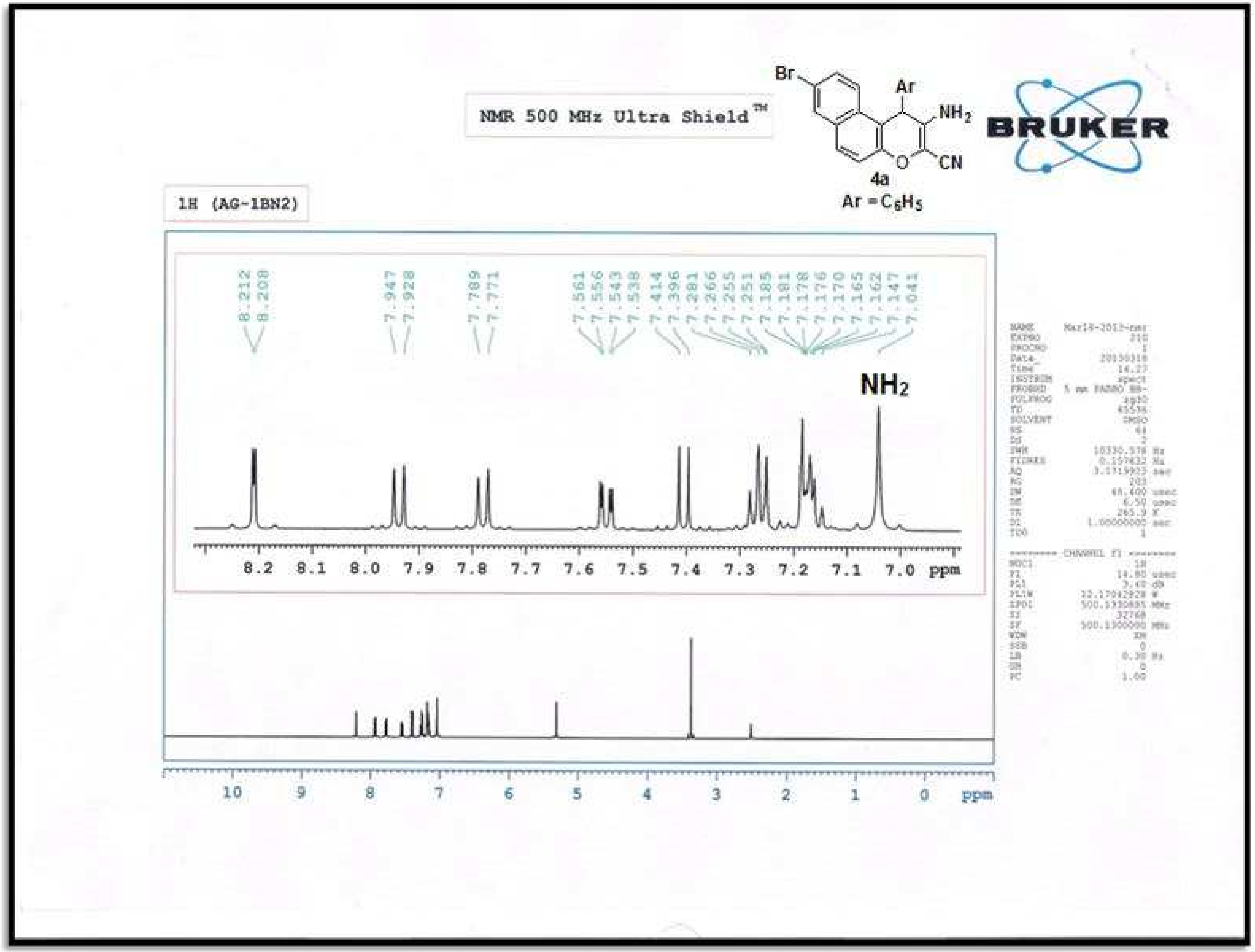
NMR 500 MHz Ultra Shield™
BRUKER
Br
Ar
NH2
O
CN
4a
Ar = C6H5
1H (AG-1BN2)
8.212
8.208
7.947
7.928
7.789
7.771
7.561
7.556
7.543
7.538
7.414
7.396
7.281
7.266
7.255
7.251
7.185
7.181
7.178
7.176
7.170
7.165
7.162
7.147
7.041
NH2
8.2 8.1 8.0 7.9 7.8 7.7 7.6 7.5 7.4 7.3 7.2 7.1 7.0 ppm
10 9 8 7 6 5 4 3 2 1 0 ppm

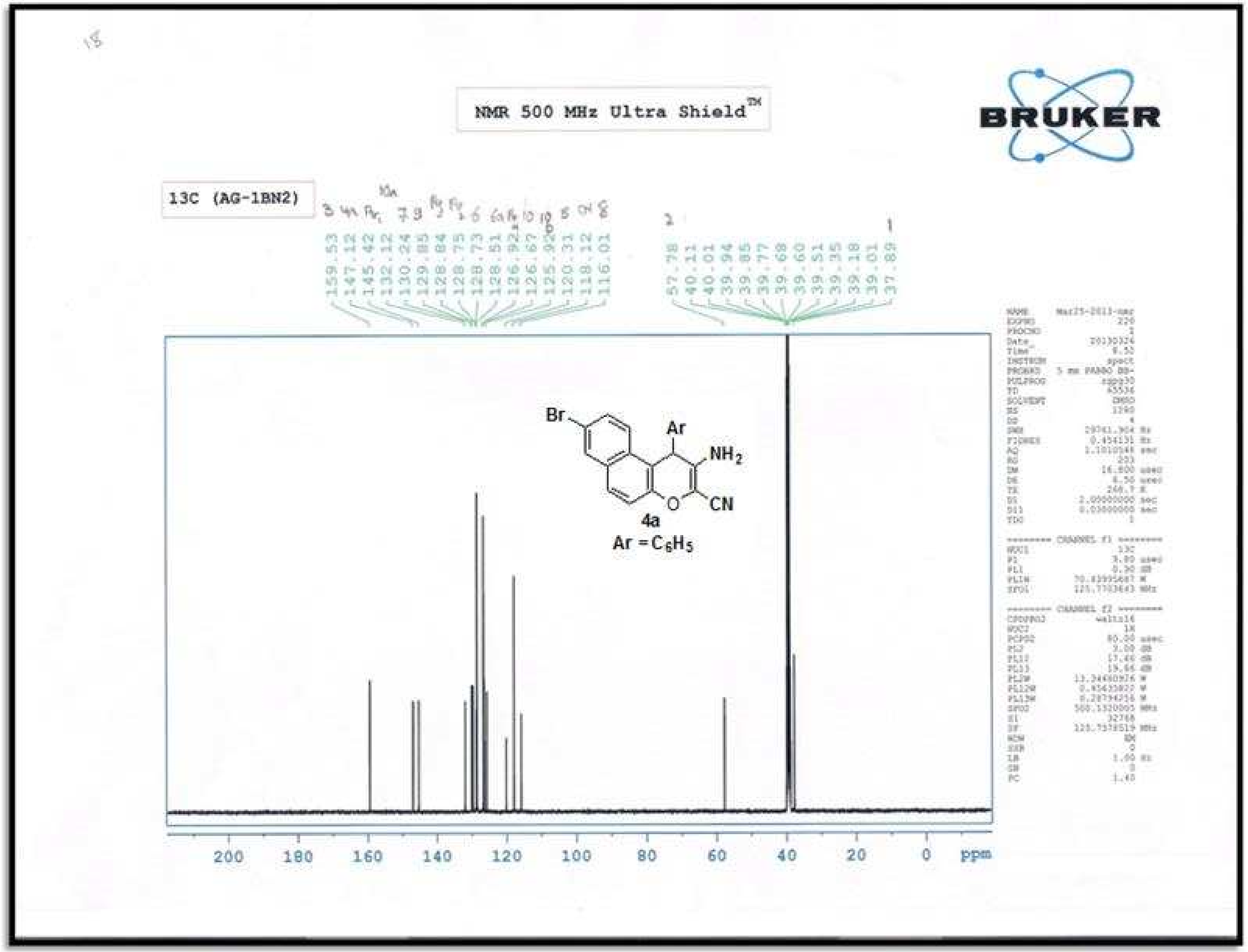
NMR 500 MHz Ultra Shield™
BRUKER
13C (AG-1BN2)
159.53
147.12
145.42
132.12
130.24
129.85
128.84
128.75
128.73
128.51
126.92
126.67
125.92
120.31
118.12
116.01
57.78
40.11
40.01
39.94
39.85
39.77
39.68
39.60
39.51
39.35
39.18
39.01
37.89
Br
Ar
NH2
CN
O
4a
Ar = C6H5
200
180
160
140
120
100
80
60
40
20
0
ppm

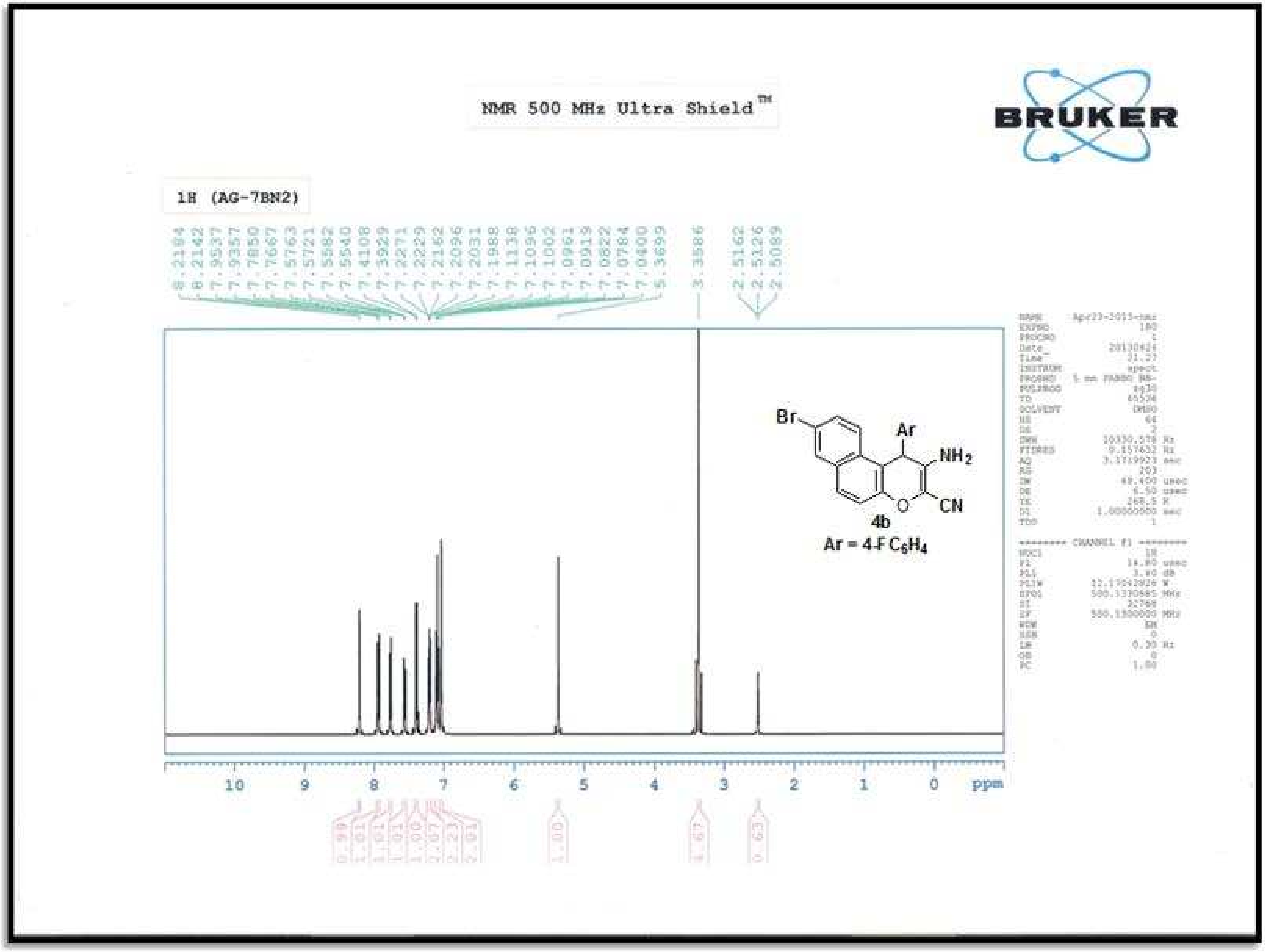
NMR 500 MHz Ultra Shield™
BRUKER
1H (AG-7BN2)
8.2184
8.2142
7.9537
7.9357
7.7850
7.7667
7.5763
7.5721
7.5582
7.5540
7.4108
7.3929
7.2271
7.2229
7.2162
7.2096
7.2031
7.1988
7.1138
7.1096
7.1002
7.0961
7.0919
7.0822
7.0784
7.0400
5.3699
3.3586
2.5162
2.5126
2.5089
Br
Ar
NH2
O
CN
4b
Ar = 4-F C6H4
10
9
8
7
6
5
4
3
2
1
0
ppm

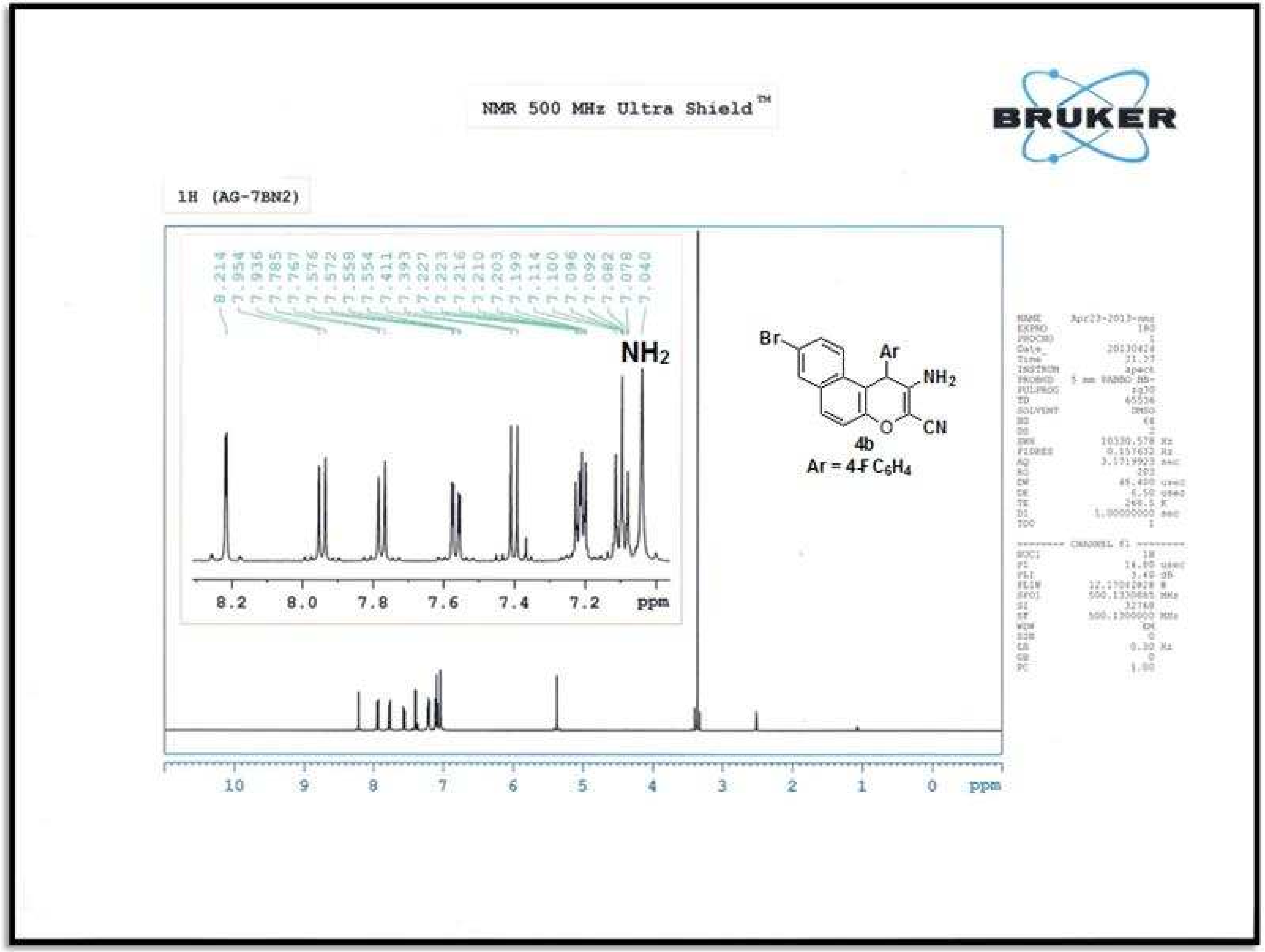
NMR 500 MHz Ultra Shield™
BRUKER
1H (AG-7BN2)
8.214 7.954 7.936 7.785 7.767 7.576 7.572 7.558 7.554 7.411 7.393 7.227 7.223 7.216 7.210 7.203 7.199 7.114 7.100 7.096 7.092 7.082 7.078 7.040
NH2
Br
Ar
NH2
CN
O
4b
Ar = 4-F C6H4
ppm

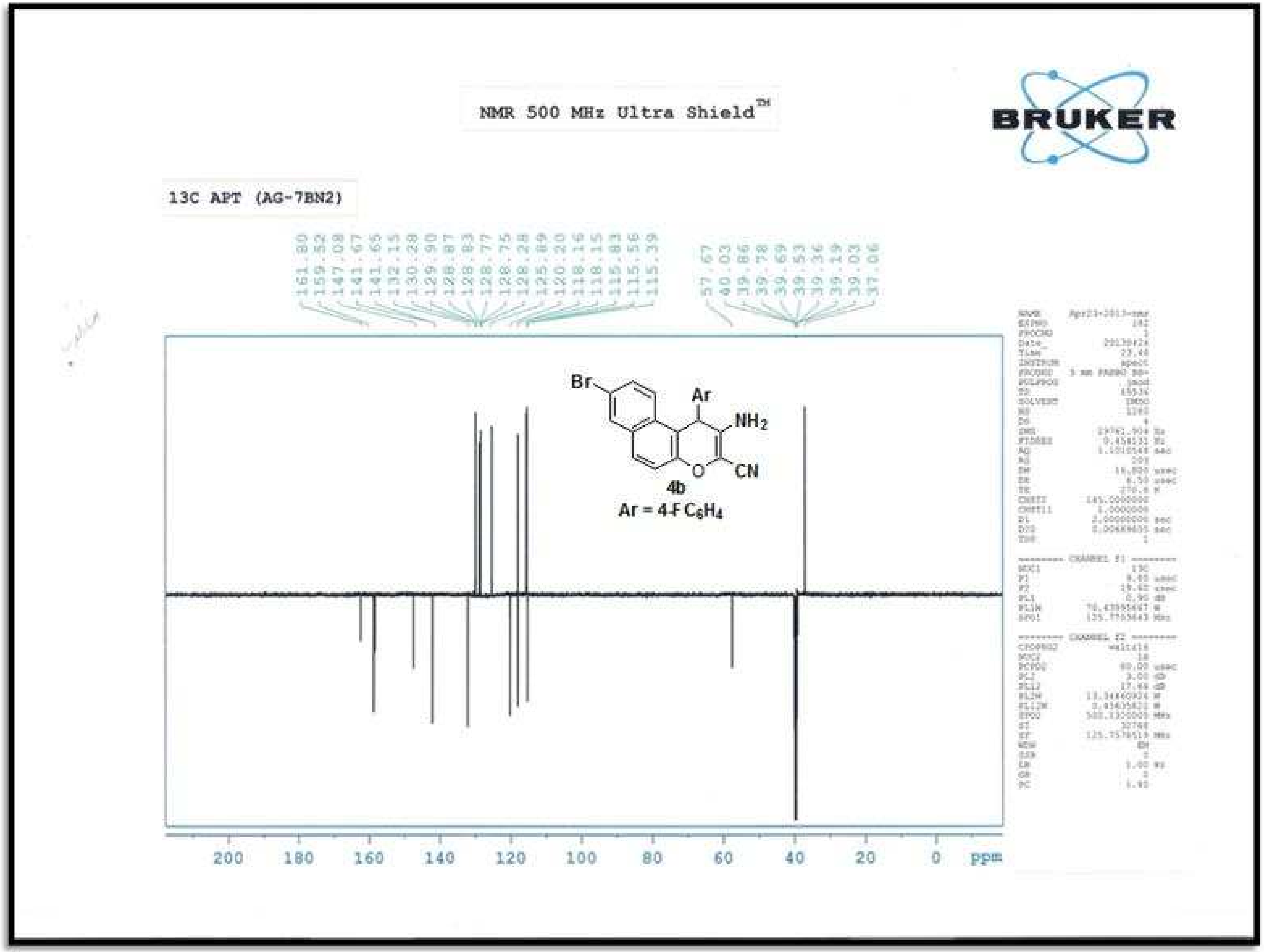
NMR 500 MHz Ultra Shield™
13C APT (AG-7BN2)
161.80 159.52 147.08 141.67 141.65 132.15 130.28 129.90 128.87 128.83 128.77 128.75 128.28 125.89 120.20 118.16 118.15 115.83 115.56 115.39
57.67 40.03 39.86 39.78 39.69 39.53 39.36 39.19 39.03 37.06
Br
Ar
NH2
O
CN
4b
Ar = 4-F C6H4
200 180 160 140 120 100 80 60 40 20 0 ppm

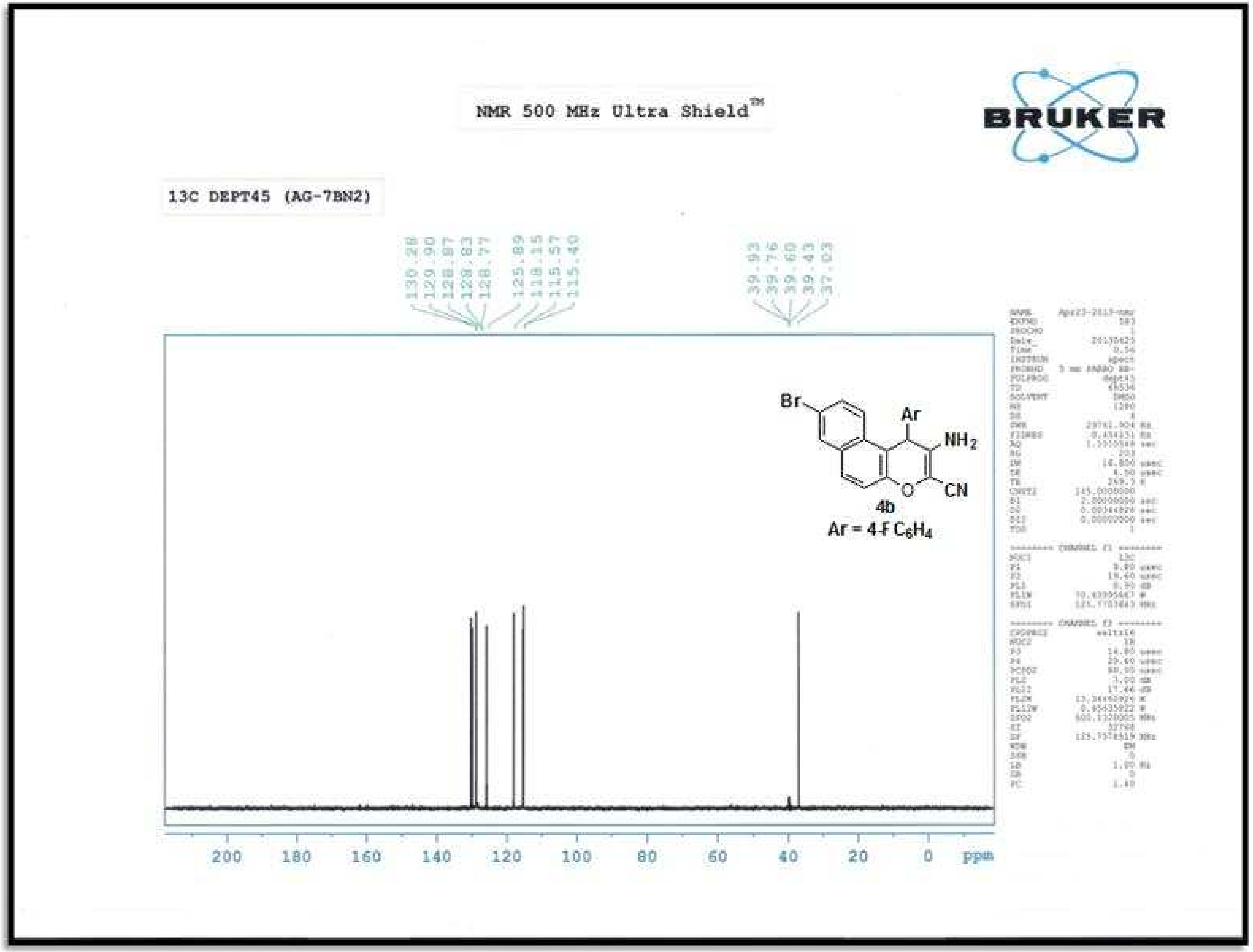
NMR 500 MHz Ultra Shield™
BRUKER
13C DEPT45 (AG-7BN2)
130.28
129.90
128.87
128.83
128.77
125.89
118.15
115.57
115.40
39.93
39.76
39.60
39.43
37.03
Br
Ar
NH2
CN
O
4b
Ar = 4-F C6H4
200
180
160
140
120
100
80
60
40
20
0
ppm

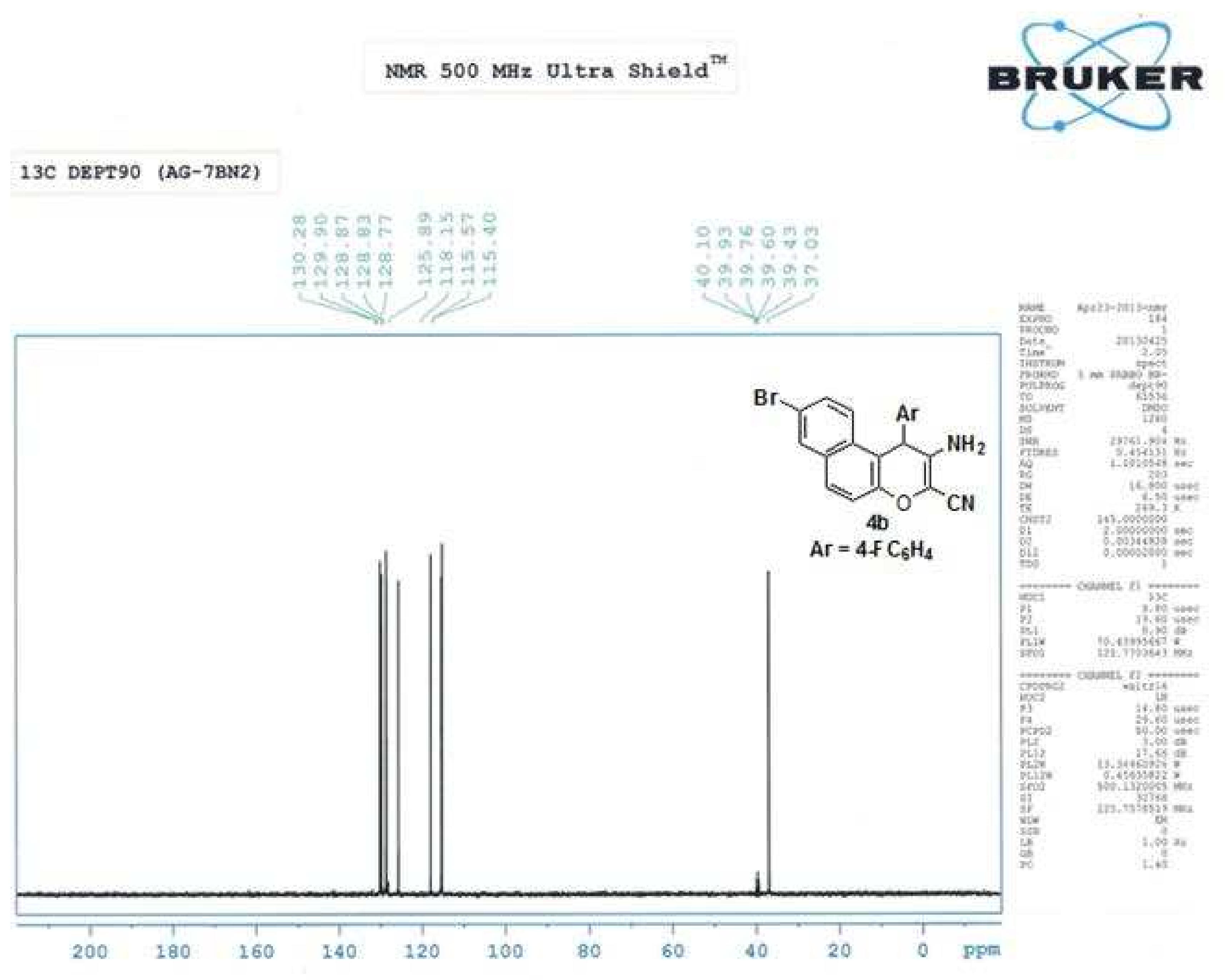
NMR 500 MHz Ultra Shield™
BRUKER
13C DEPT90 (AG-7BN2)
130.28
129.90
128.87
128.83
128.77
125.89
118.15
115.57
115.40
40.10
39.93
39.76
39.60
39.43
37.03
Br
Ar
NH2
CN
O
4b
Ar = 4-F C6H4
200 180 160 140 120 100 80 60 40 20 0 ppm

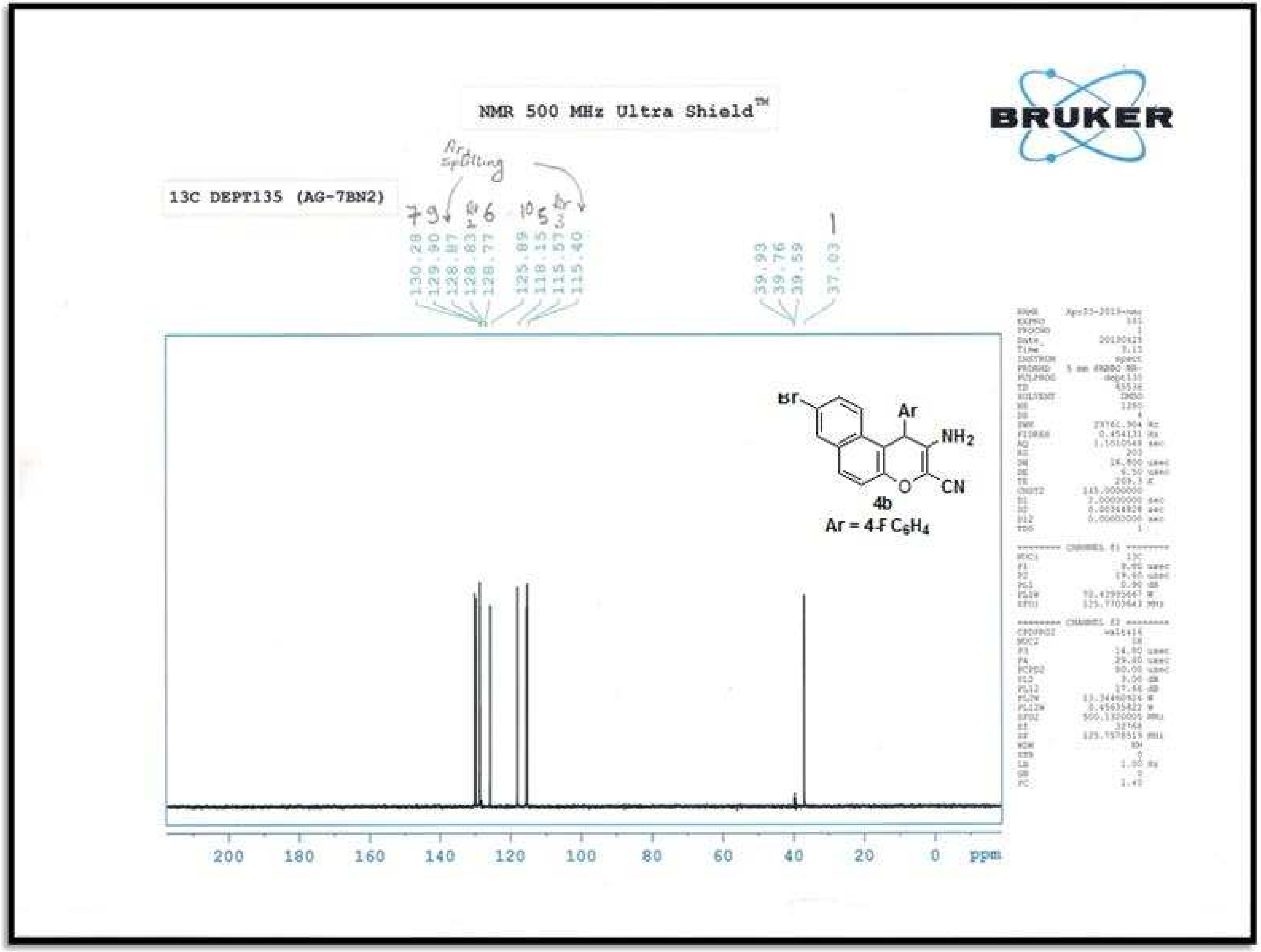

NMR 500 MHz Ultra Shield™
13C DEPT135 (AG-7BN2)
130.28
129.90
128.87
128.83
128.77
125.89
118.15
115.57
115.40
39.93
39.76
39.59
37.03
Ar splitting
Br
Ar
NH2
CN
O
4b
Ar = 4-F C6H4
200
180
160
140
120
100
80
60
40
20
0
ppm

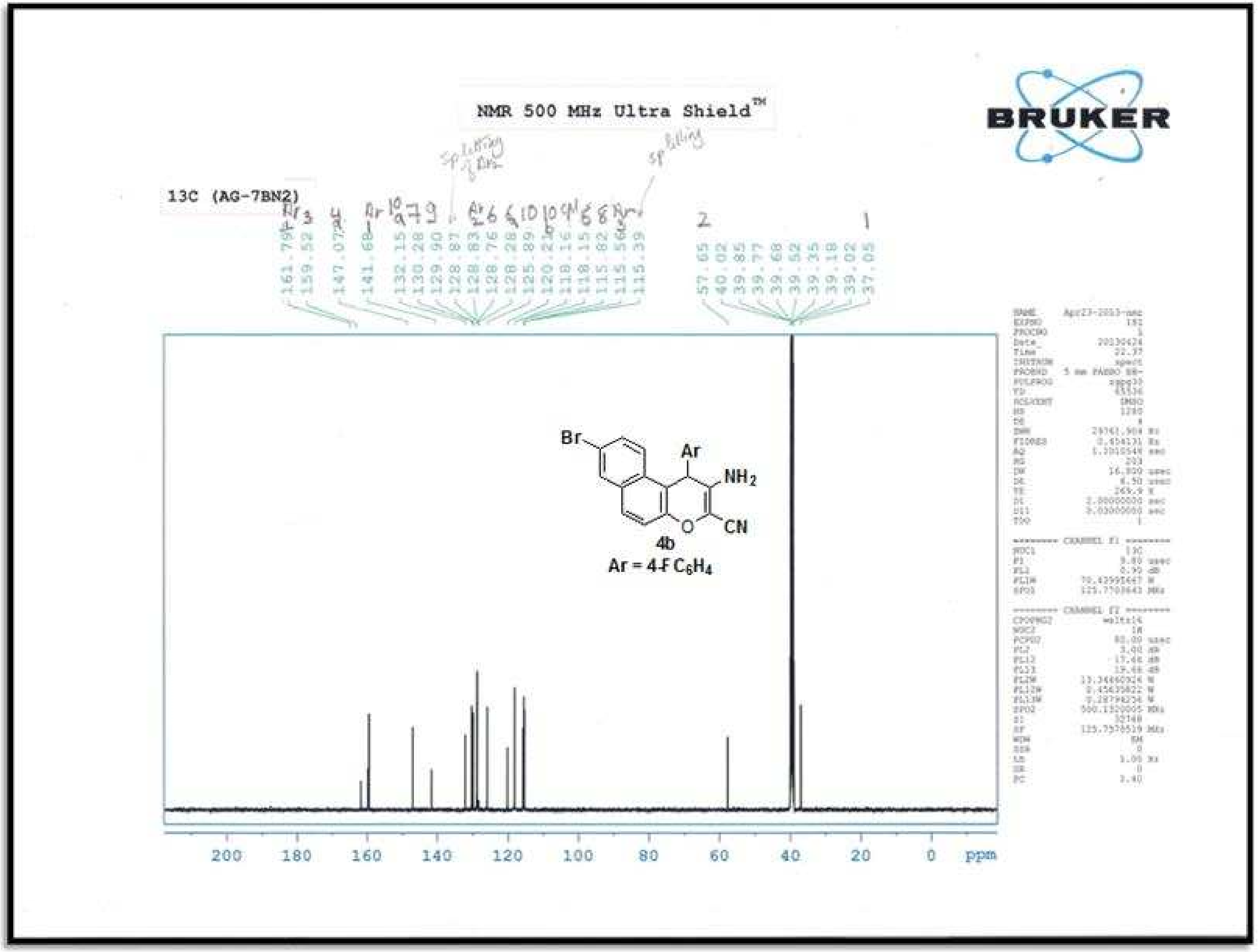
NMR 500 MHz Ultra Shield™
BRUKER
13C (AG-7BN2)
161.79
159.52
147.07
141.68
132.15
130.28
129.90
128.87
128.83
128.76
128.28
125.89
120.21
118.16
118.15
115.82
115.56
115.39
57.65
40.02
39.85
39.77
39.68
39.52
39.35
39.18
39.02
37.05
Br
Ar
NH2
O
CN
4b
Ar = 4-F C6H4
200
180
160
140
120
100
80
60
40
20
0
ppm

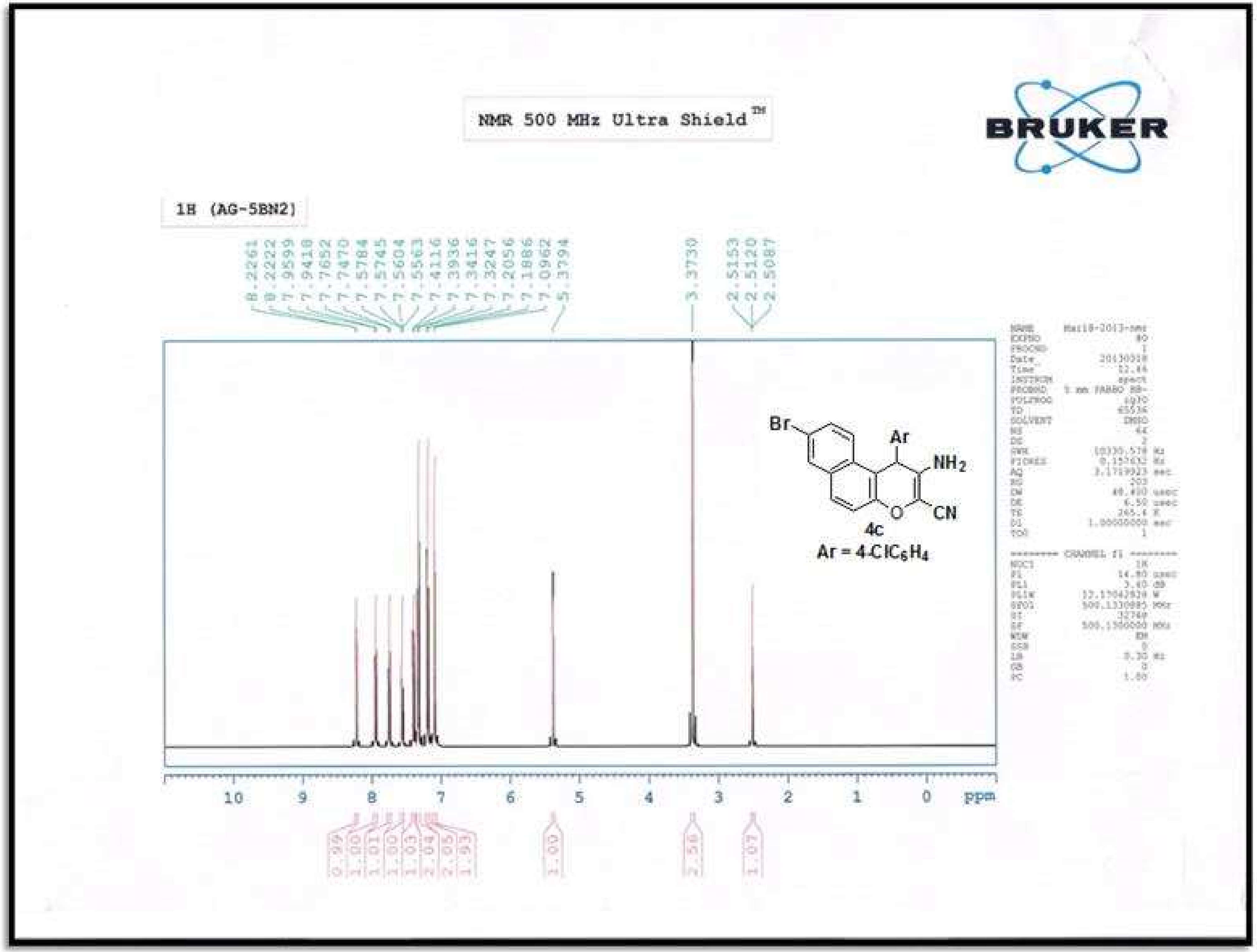

NMR 500 MHz Ultra Shield™
BRUKER
1H (AG-5BN2)
8.2261
8.2222
7.9599
7.9418
7.7652
7.7470
7.5784
7.5745
7.5604
7.5563
7.4116
7.3936
7.3416
7.3247
7.2056
7.1886
7.0962
5.3794
3.3730
2.5153
2.5120
2.5087
Br
Ar
NH2
O
CN
4c
Ar = 4-ClC6H4
ppm

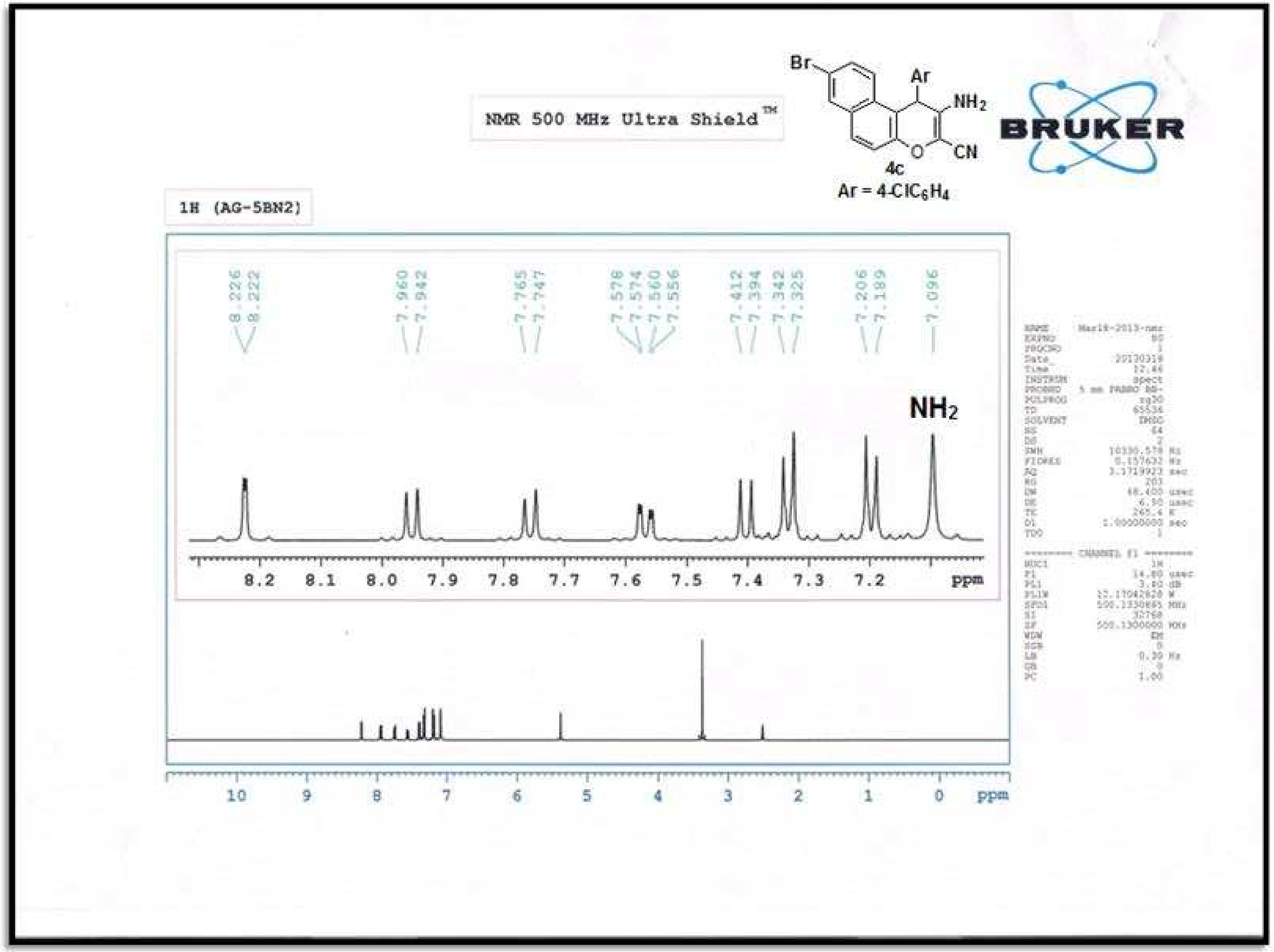
NMR 500 MHz Ultra Shield™
Br
Ar
NH2
CN
O
4c
Ar = 4-ClC6H4
BRUKER
1H (AG-5BN2)
8.226
8.222
7.960
7.942
7.765
7.747
7.578
7.574
7.560
7.556
7.412
7.394
7.342
7.325
7.206
7.189
7.096
NH2
ppm

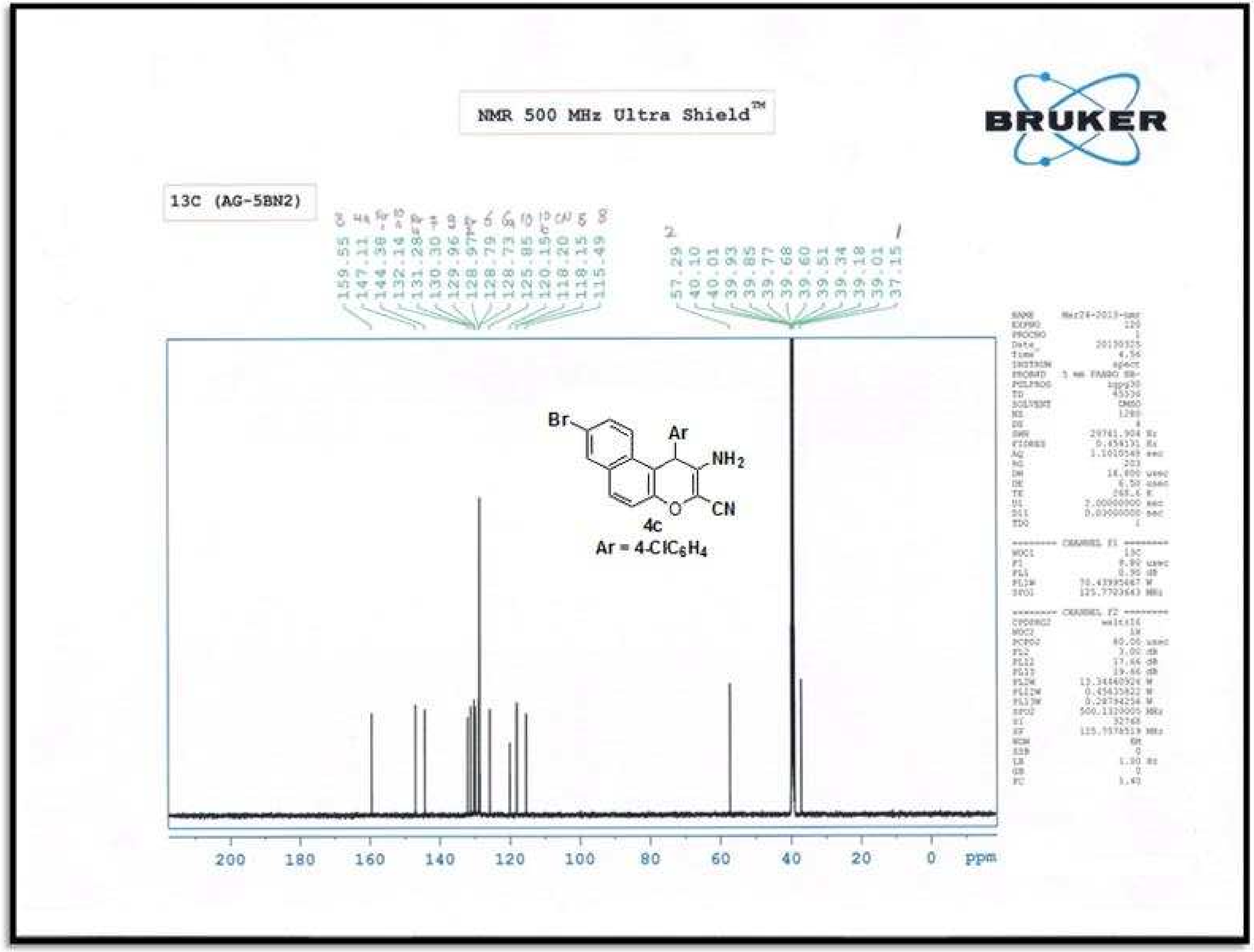
NMR 500 MHz Ultra Shield™
BRUKER
13C (AG-5BN2)
159.55
147.11
144.38
132.14
131.28
130.30
129.96
128.97
128.79
128.73
125.85
120.15
118.20
118.15
115.49
57.29
40.10
40.01
39.93
39.85
39.77
39.68
39.60
39.51
39.34
39.18
39.01
37.15
Br
Ar
NH2
CN
O
4c
Ar = 4-ClC6H4
200
180
160
140
120
100
80
60
40
20
0
ppm

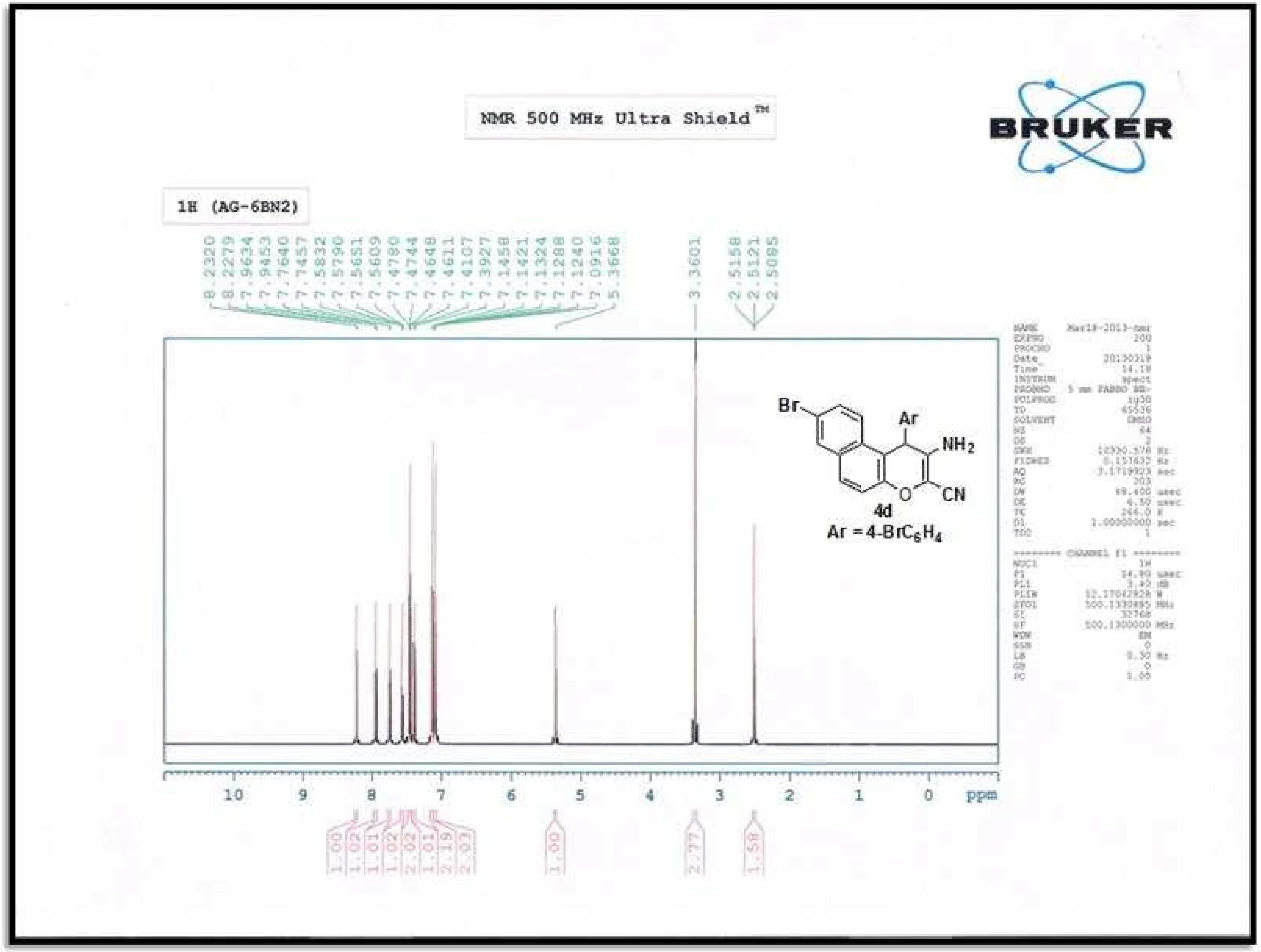
NMR 500 MHz Ultra Shield™
BRUKER
1H (AG-6BN2)
8.2320
8.2279
7.9634
7.9453
7.7640
7.7457
7.5832
7.5790
7.5651
7.5609
7.4780
7.4744
7.4648
7.4611
7.4107
7.3927
7.1458
7.1421
7.1324
7.1288
7.1240
7.0916
5.3668
3.3601
2.5158
2.5121
2.5085
Br
Ar
NH2
CN
O
4d
Ar = 4-BrC6H4
10 9 8 7 6 5 4 3 2 1 0 ppm
1.00 1.02 1.01 1.02 2.02 1.01 2.19 2.03
1.00
2.77
1.58

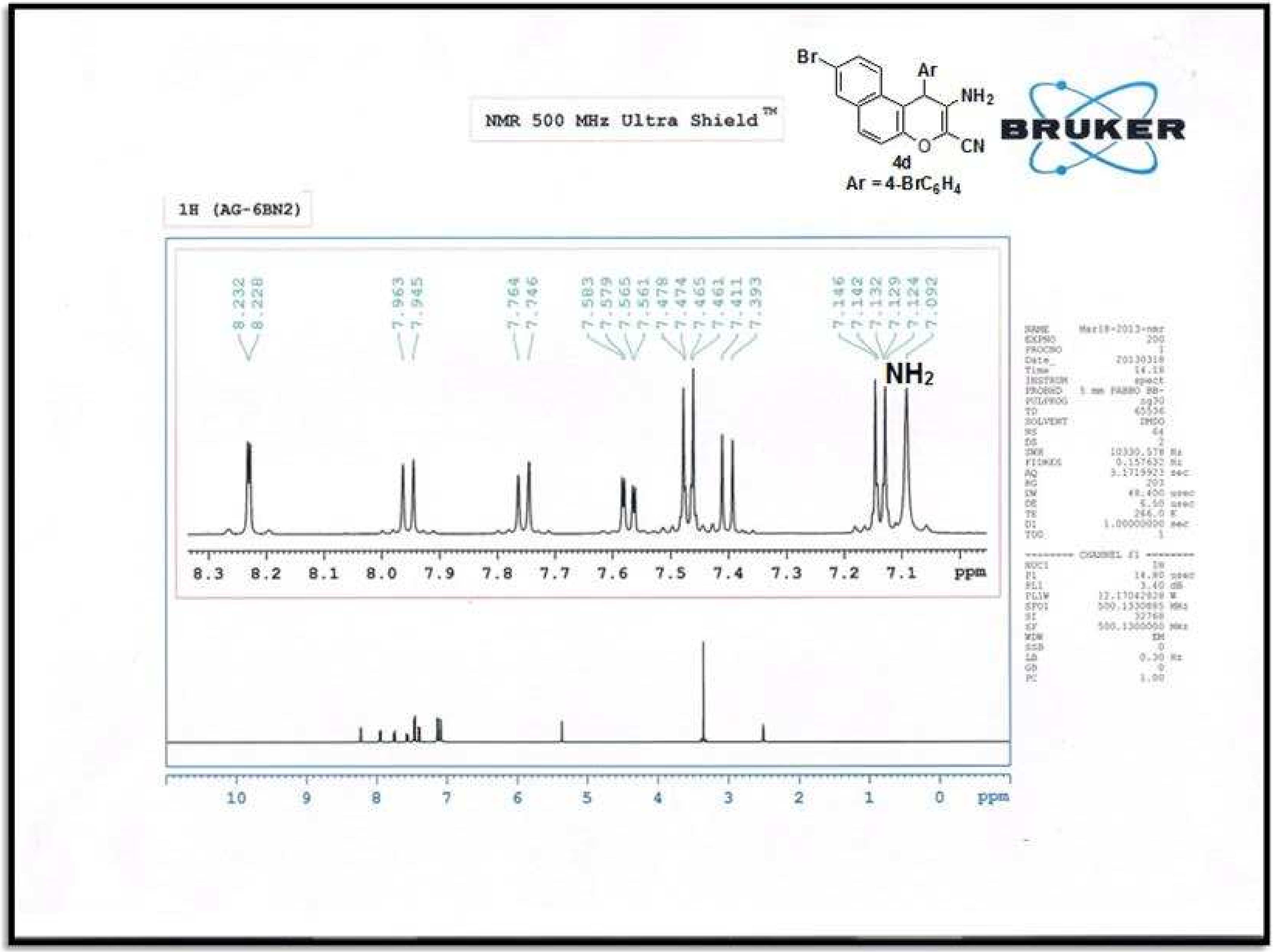
NMR 500 MHz Ultra Shield™
BRUKER
4d
Ar = 4-BrC6H4
1H (AG-6BN2)
8.232
8.228
7.963
7.945
7.764
7.746
7.583
7.579
7.565
7.561
7.478
7.474
7.465
7.461
7.411
7.393
7.146
7.142
7.132
7.129
7.124
7.092
NH2
ppm

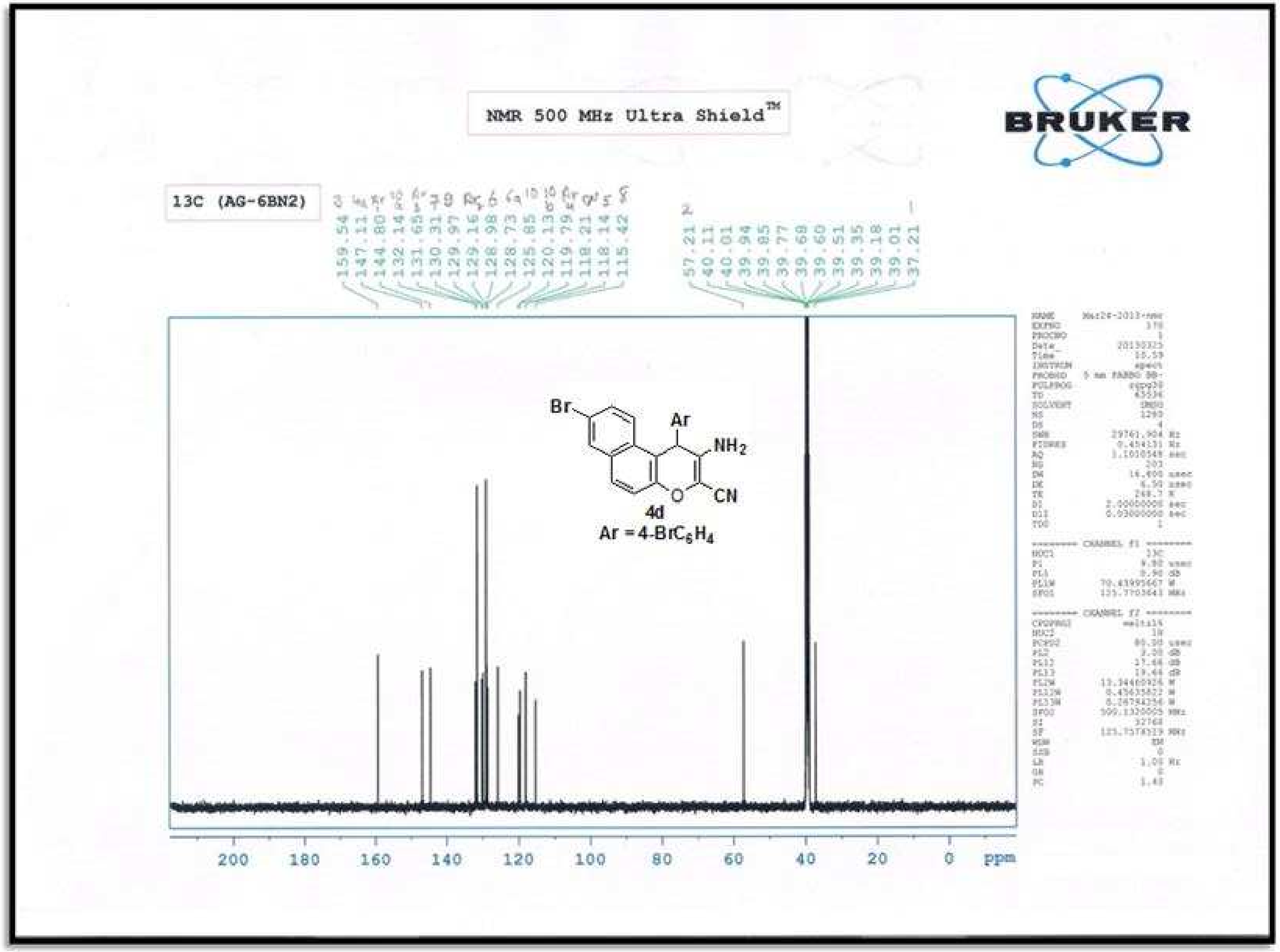
NMR 500 MHz Ultra Shield™
BRUKER
13C (AG-6BN2)
159.54
147.11
144.80
132.14
131.65
130.31
129.97
129.16
128.98
128.73
125.85
120.13
119.79
118.21
118.14
115.42
57.21
40.11
40.01
39.94
39.85
39.77
39.68
39.60
39.51
39.35
39.18
39.01
37.21
Br
Ar
NH2
O
CN
4d
Ar = 4-BrC6H4
200
180
160
140
120
100
80
60
40
20
0
ppm

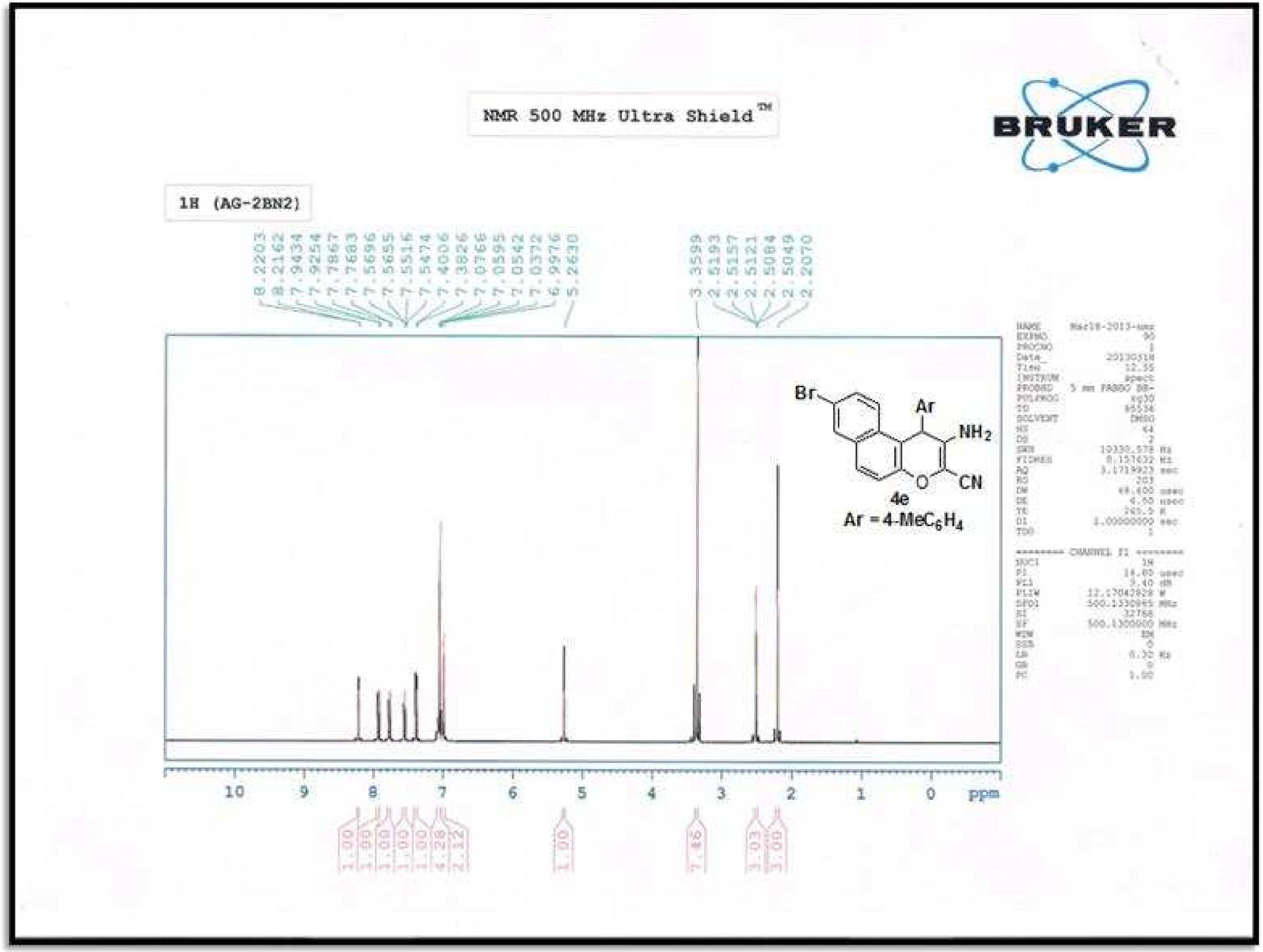

NMR 500 MHz Ultra Shield™
BRUKER
1H (AG-2BN2)
8.2203
8.2162
7.9434
7.9254
7.7867
7.7683
7.5696
7.5655
7.5516
7.5474
7.4006
7.3826
7.0766
7.0595
7.0542
7.0372
6.9976
5.2630
3.3599
2.5193
2.5157
2.5121
2.5084
2.5049
2.2070
Br
Ar
NH2
CN
O
4e
Ar = 4-MeC6H4
ppm
1.00
1.00
1.00
1.00
4.28
2.12
1.00
7.46
3.03
3.00

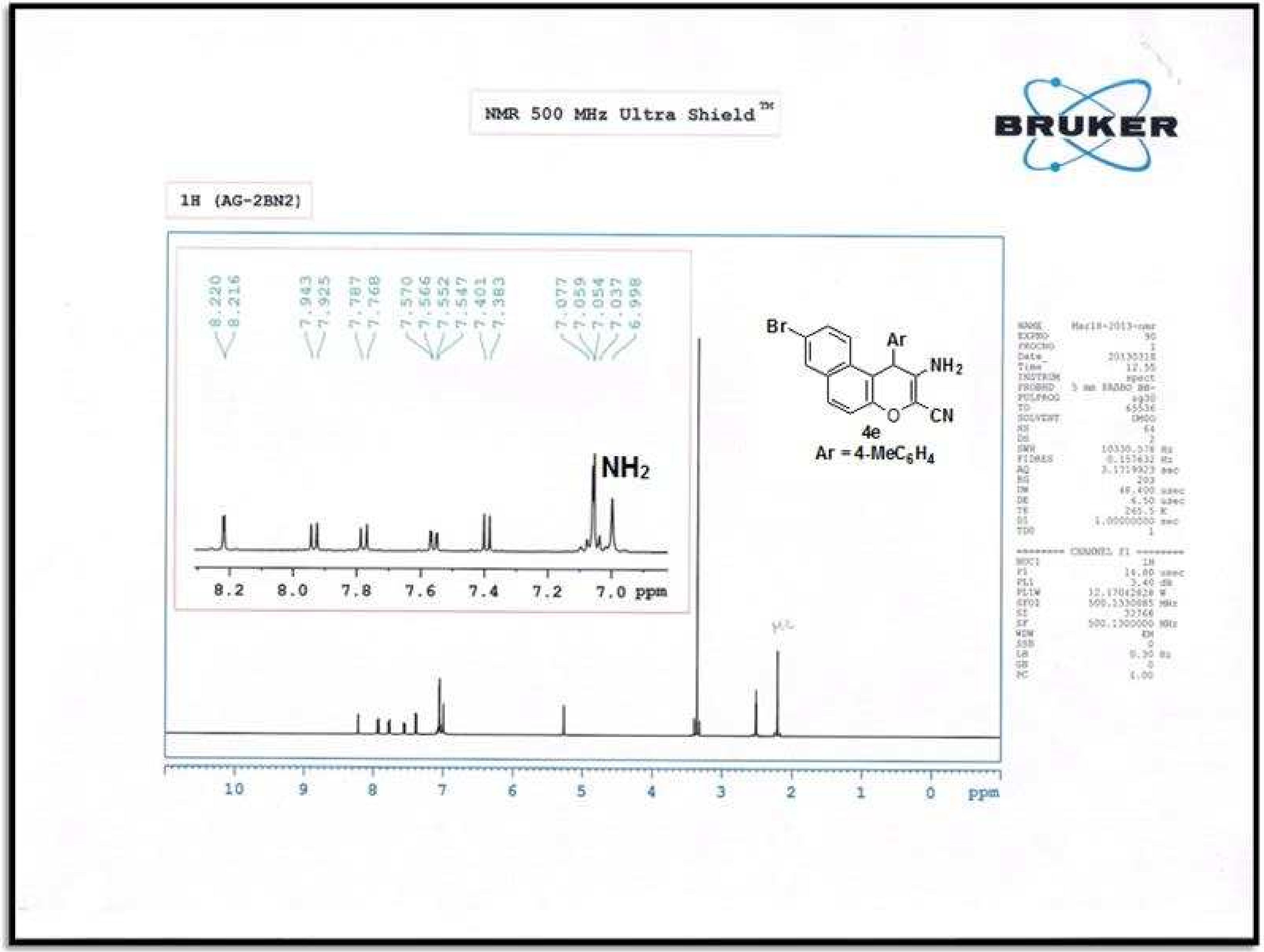
NMR 500 MHz Ultra Shield™
BRUKER
1H (AG-2BN2)
8.220
8.216
7.943
7.925
7.787
7.768
7.570
7.566
7.552
7.547
7.401
7.383
7.077
7.059
7.054
7.037
6.998
NH2
8.2 8.0 7.8 7.6 7.4 7.2 7.0 ppm
Br
Ar
NH2
O
CN
4e
Ar = 4-MeC6H4
10 9 8 7 6 5 4 3 2 1 0 ppm

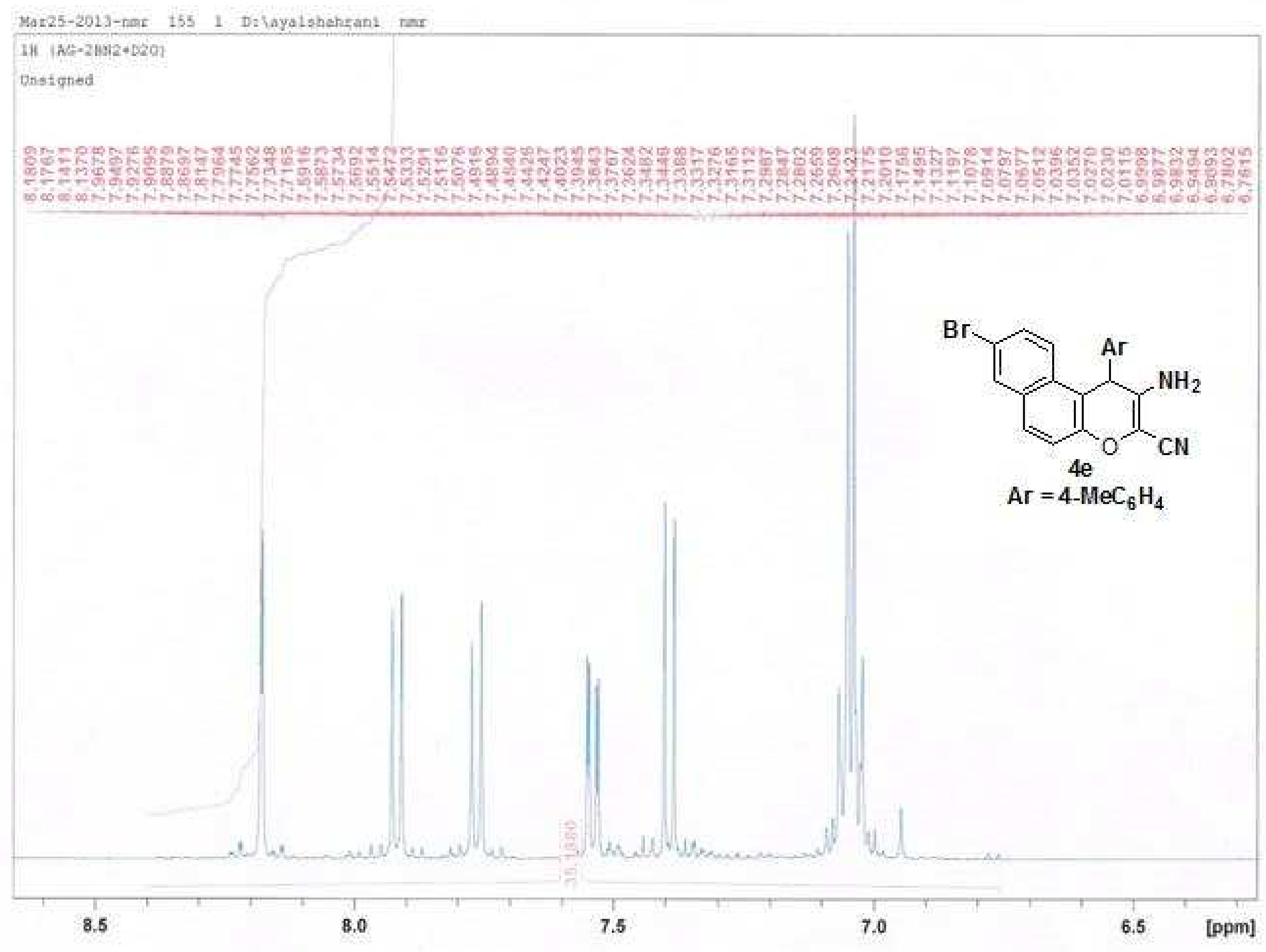

Mar25-2013-nmr 155 1 D:\ayalshahrani nmr
1H (AG-2BN2+D2O)
Unsigned
8.1809
8.1767
8.1411
8.1370
7.9678
7.9497
7.9276
7.9095
7.8879
7.8697
7.8147
7.7964
7.7745
7.7562
7.7348
7.7165
7.5916
7.5873
7.5734
7.5692
7.5514
7.5472
7.5333
7.5291
7.5116
7.5076
7.4916
7.4894
7.4540
7.4426
7.4247
7.4023
7.3945
7.3843
7.3767
7.3624
7.3482
7.3446
7.3388
7.3317
7.3276
7.3165
7.3112
7.2987
7.2847
7.2802
7.2659
7.2608
7.2422
7.2175
7.2010
7.1756
7.1495
7.1327
7.1197
7.1078
7.0914
7.0797
7.0677
7.0512
7.0396
7.0352
7.0270
7.0230
7.0116
6.9998
6.9877
6.9832
6.9494
6.9093
6.7802
6.7615
Br
Ar
NH2
O
CN
4e
Ar = 4-MeC6H4
8.5
8.0
7.5
7.0
6.5
[ppm]

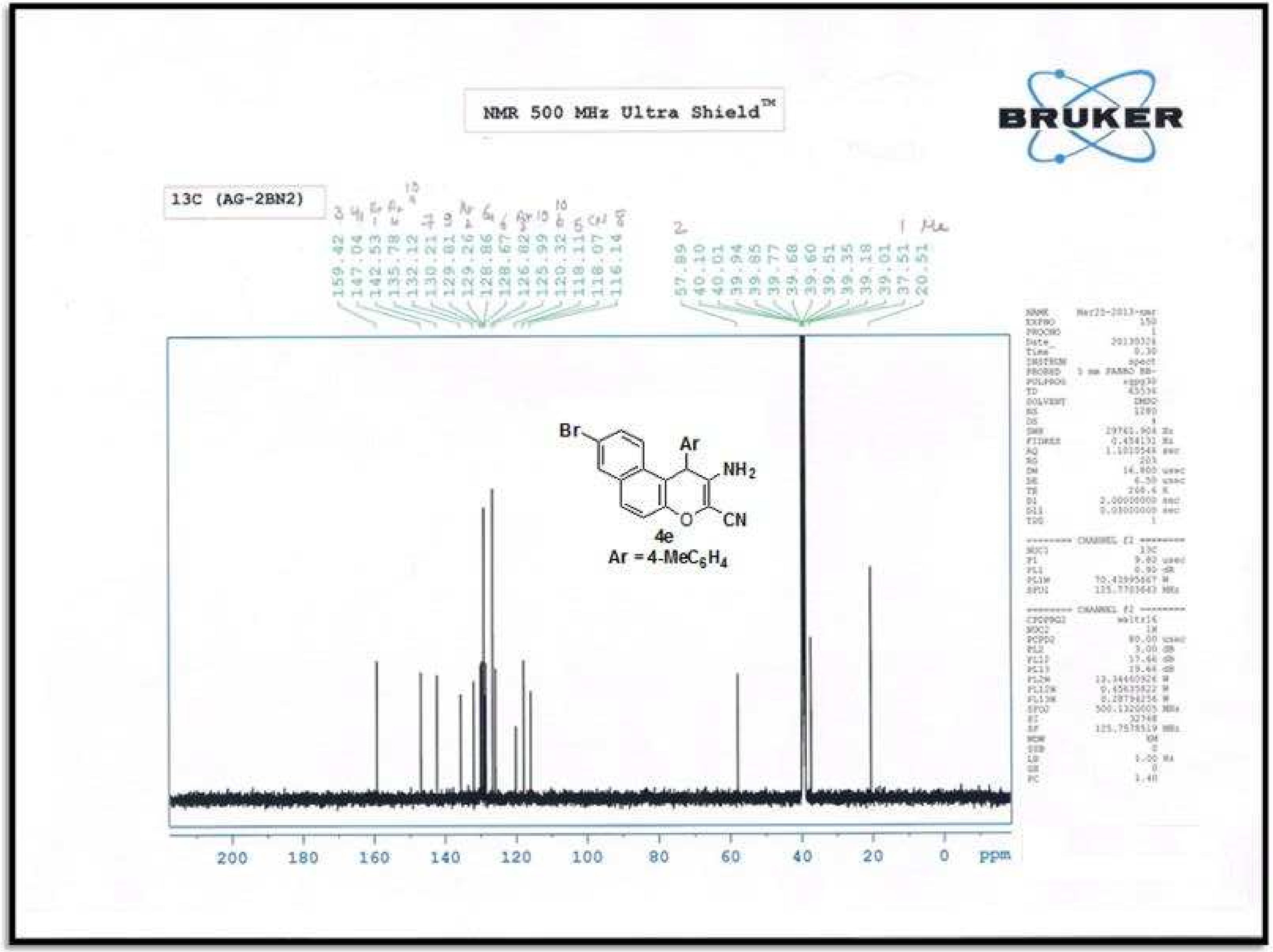
NMR 500 MHz Ultra Shield™
BRUKER
13C (AG-2BN2)
159.42
147.04
142.53
135.78
132.12
130.21
129.81
129.26
128.86
128.67
126.82
125.99
120.32
118.11
118.07
116.14
57.89
40.10
40.01
39.94
39.85
39.77
39.68
39.60
39.51
39.35
39.18
39.01
37.51
20.51
Br
Ar
NH2
CN
O
4e
Ar = 4-MeC6H4
200
180
160
140
120
100
80
60
40
20
0
ppm

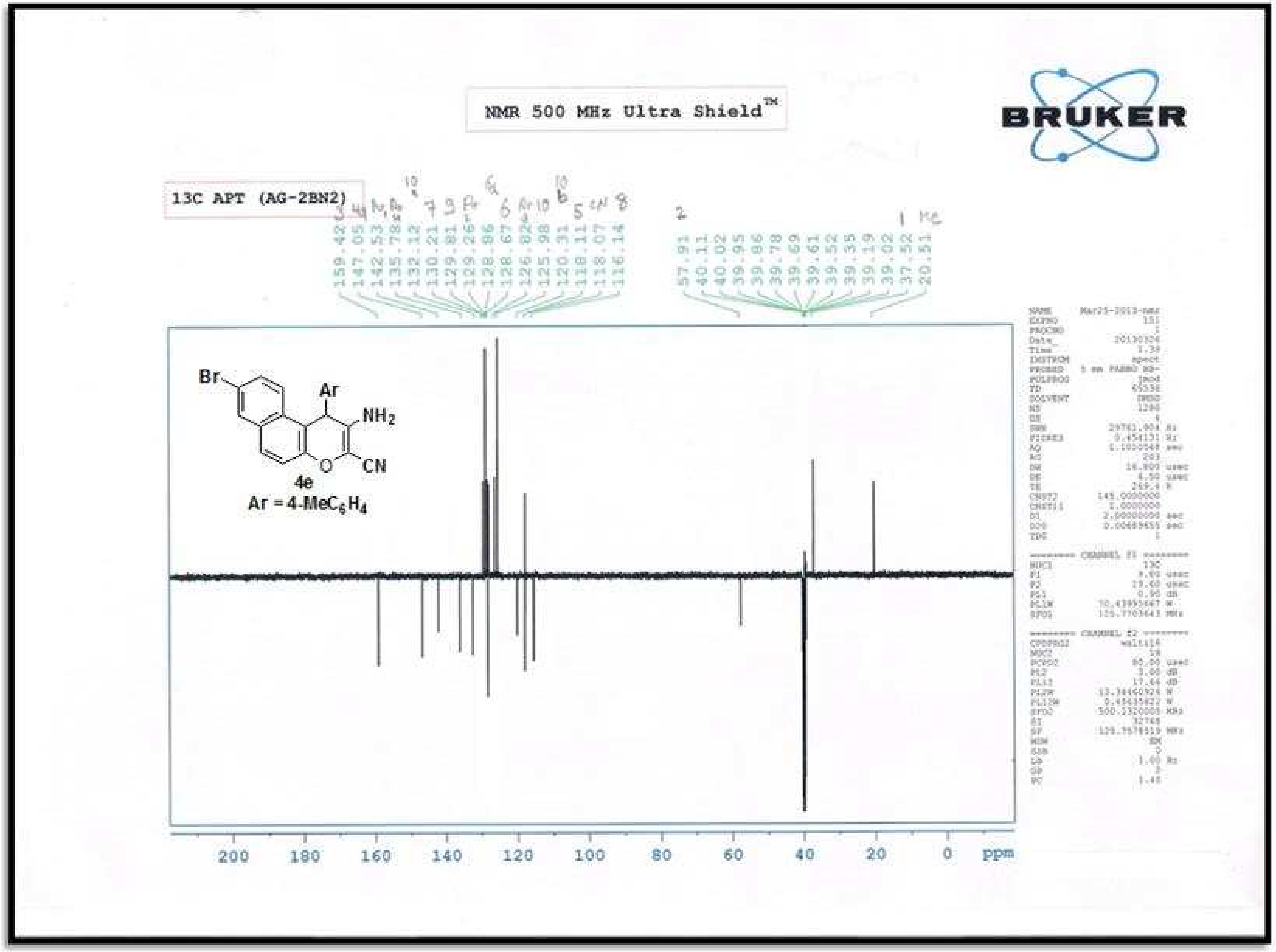
NMR 500 MHz Ultra Shield™
BRUKER
13C APT (AG-2BN2)
159.42
147.05
142.53
135.78
132.12
130.21
129.81
129.26
128.86
128.67
126.82
125.98
120.31
118.11
118.07
116.14
57.91
40.11
40.02
39.95
39.86
39.78
39.69
39.61
39.52
39.35
39.19
39.02
37.52
20.51
Br
Ar
NH2
CN
O
4e
Ar = 4-MeC6H4
200
180
160
140
120
100
80
60
40
20
0
ppm

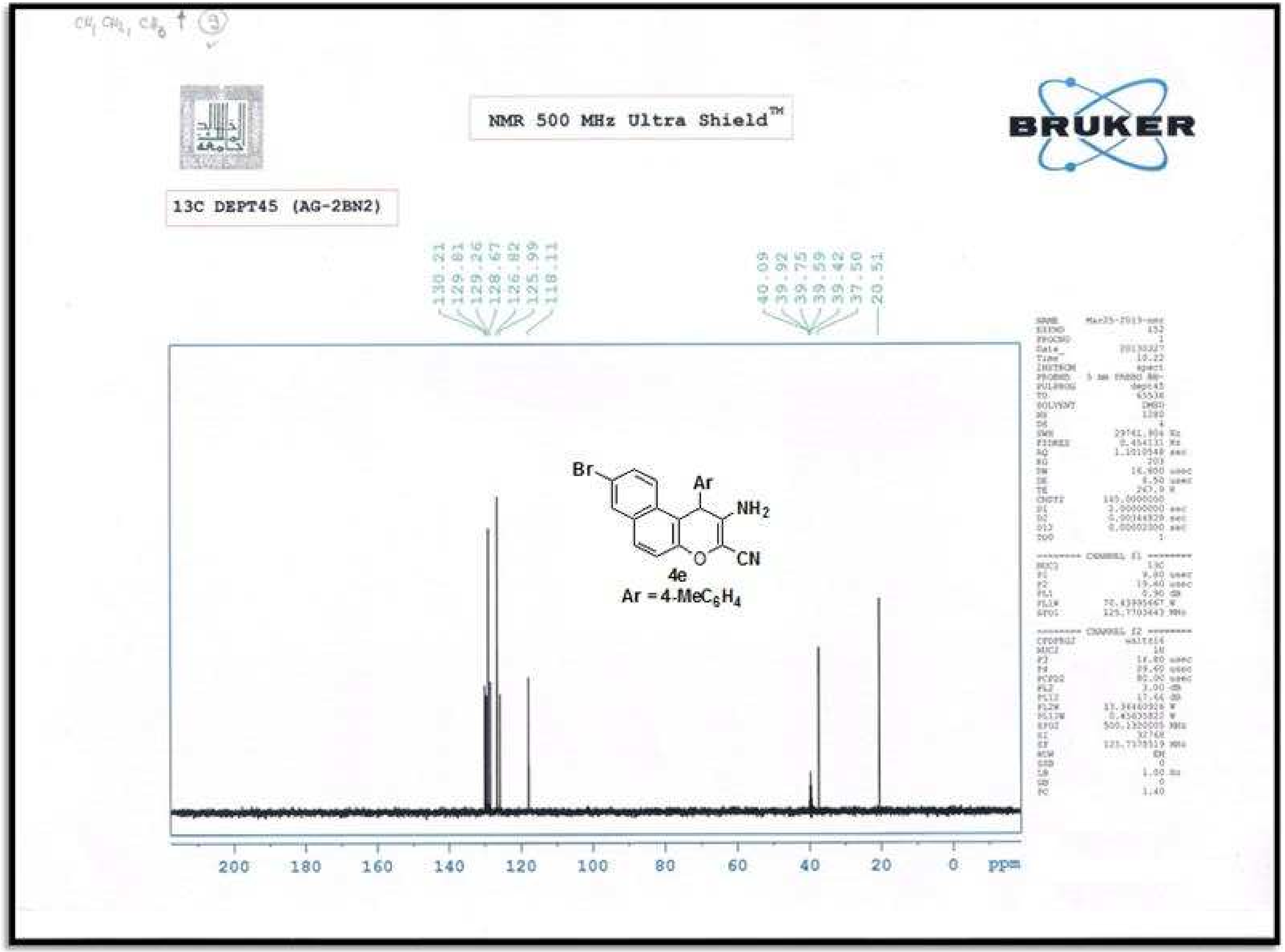

NMR 500 MHz Ultra Shield™
BRUKER
13C DEPT45 (AG-2BN2)
130.21
129.81
129.26
128.67
126.82
125.99
118.11
40.09
39.92
39.75
39.59
39.42
37.50
20.51
Br
Ar
NH2
O
CN
4e
Ar = 4-MeC6H4
200 180 160 140 120 100 80 60 40 20 0 ppm

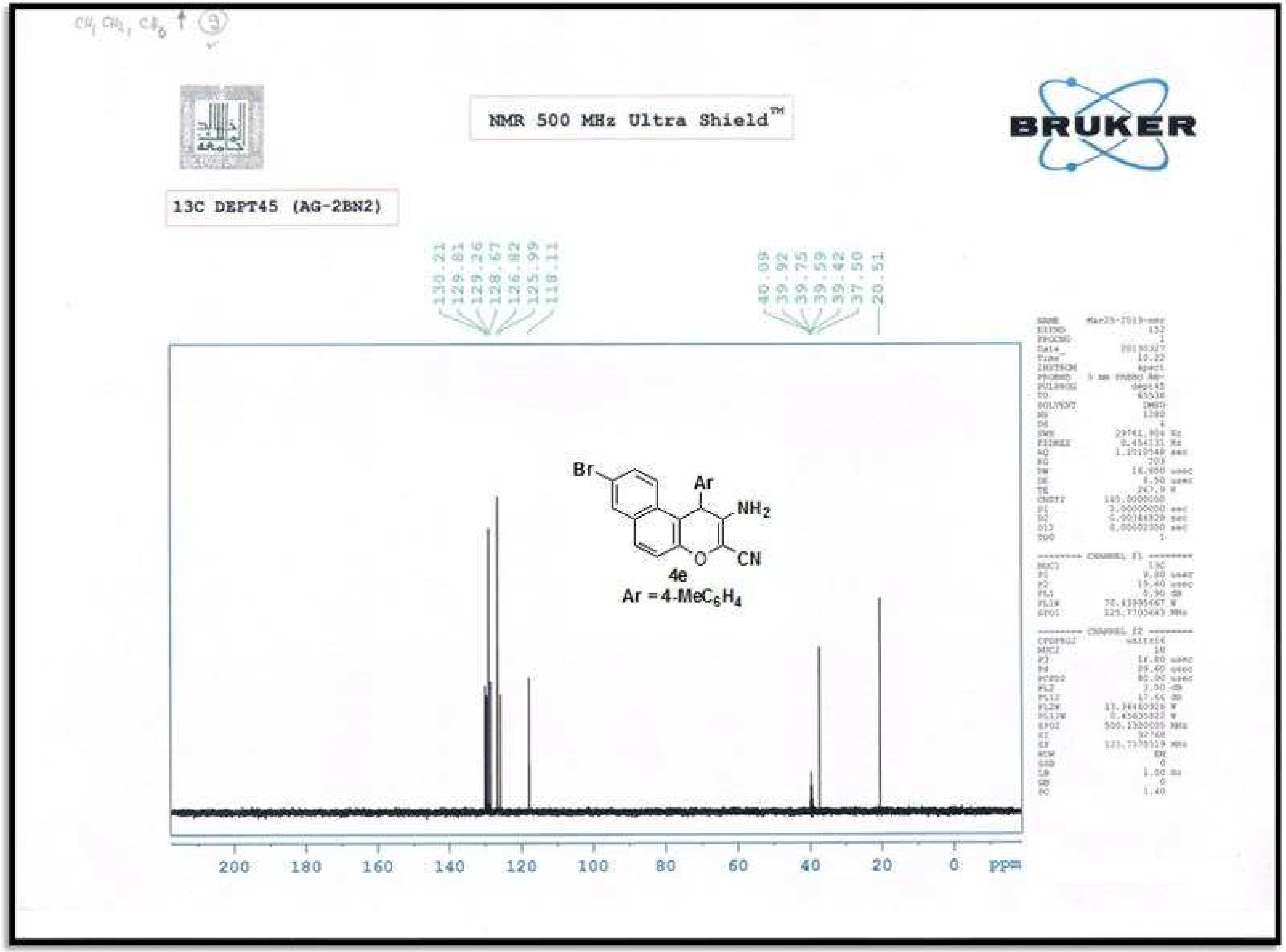
NMR 500 MHz Ultra Shield™
BRUKER
13C DEPT45 (AG-2BN2)
130.21
129.81
129.26
128.67
126.82
125.99
118.11
40.09
39.92
39.75
39.59
39.42
37.50
20.51
Br
Ar
NH2
O
CN
4e
Ar = 4-MeC6H4
200
180
160
140
120
100
80
60
40
20
0
ppm

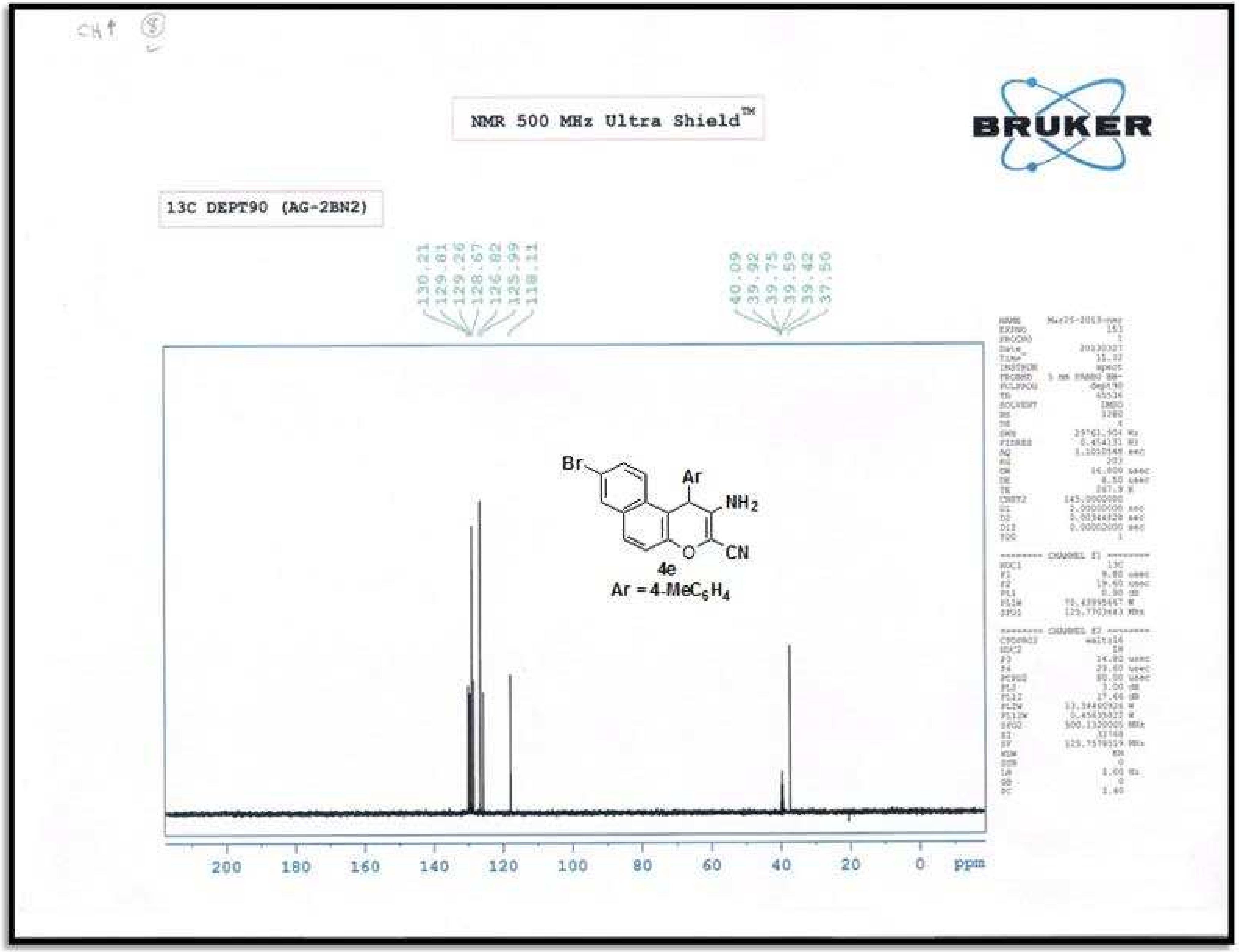

NMR 500 MHz Ultra Shield™
BRUKER
13C DEPT90 (AG-2BN2)
130.21
129.81
129.26
128.67
126.82
125.99
118.11
40.09
39.92
39.75
39.59
39.42
37.50
Br
Ar
NH2
CN
O
4e
Ar = 4-MeC6H4
200
180
160
140
120
100
80
60
40
20
0
ppm

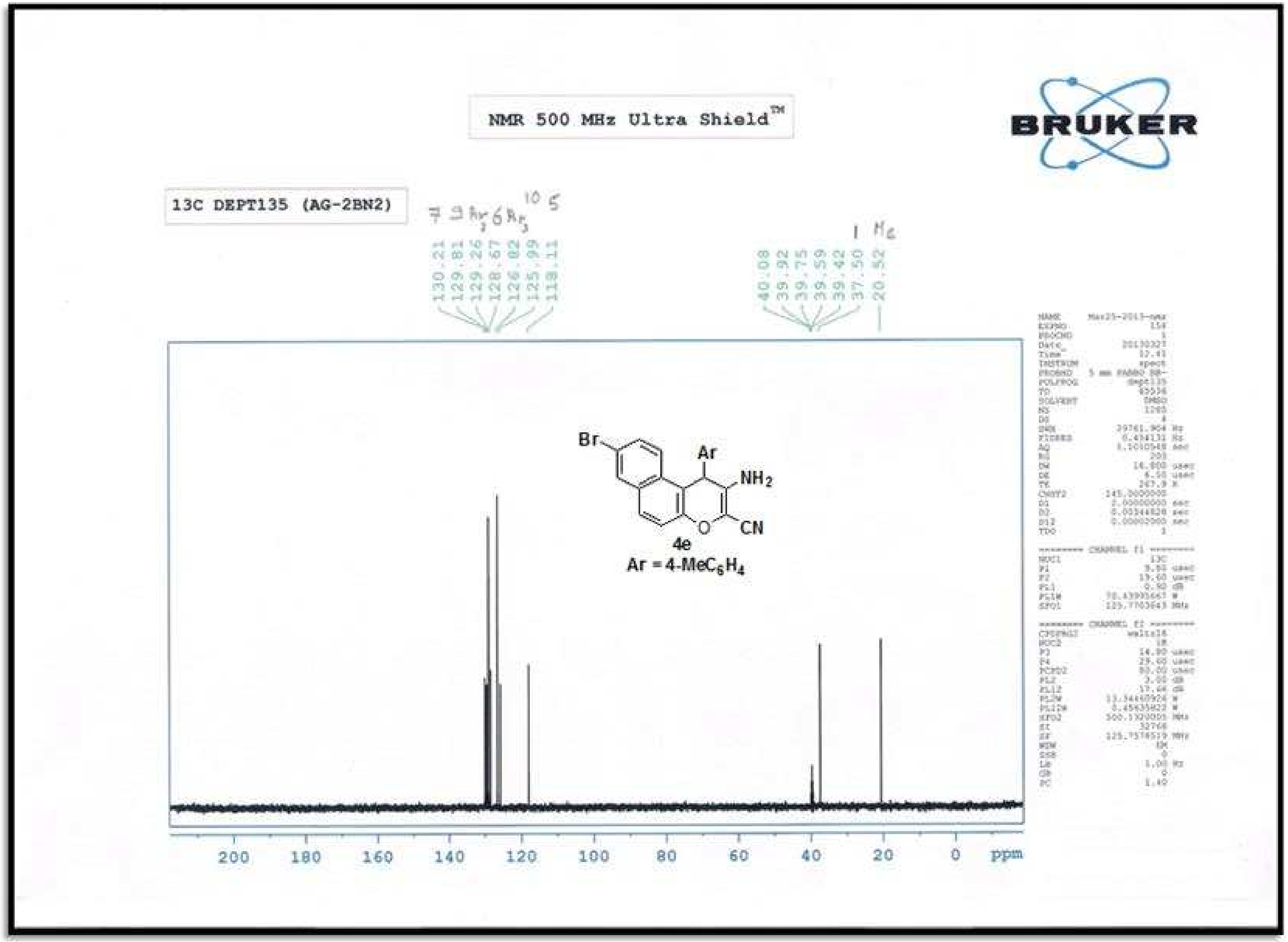
NMR 500 MHz Ultra Shield™
BRUKER
13C DEPT135 (AG-2BN2)
130.21
129.81
129.26
128.67
126.82
125.99
118.11
40.08
39.92
39.75
39.59
39.42
37.50
20.52
4e
Ar = 4-MeC6H4
ppm

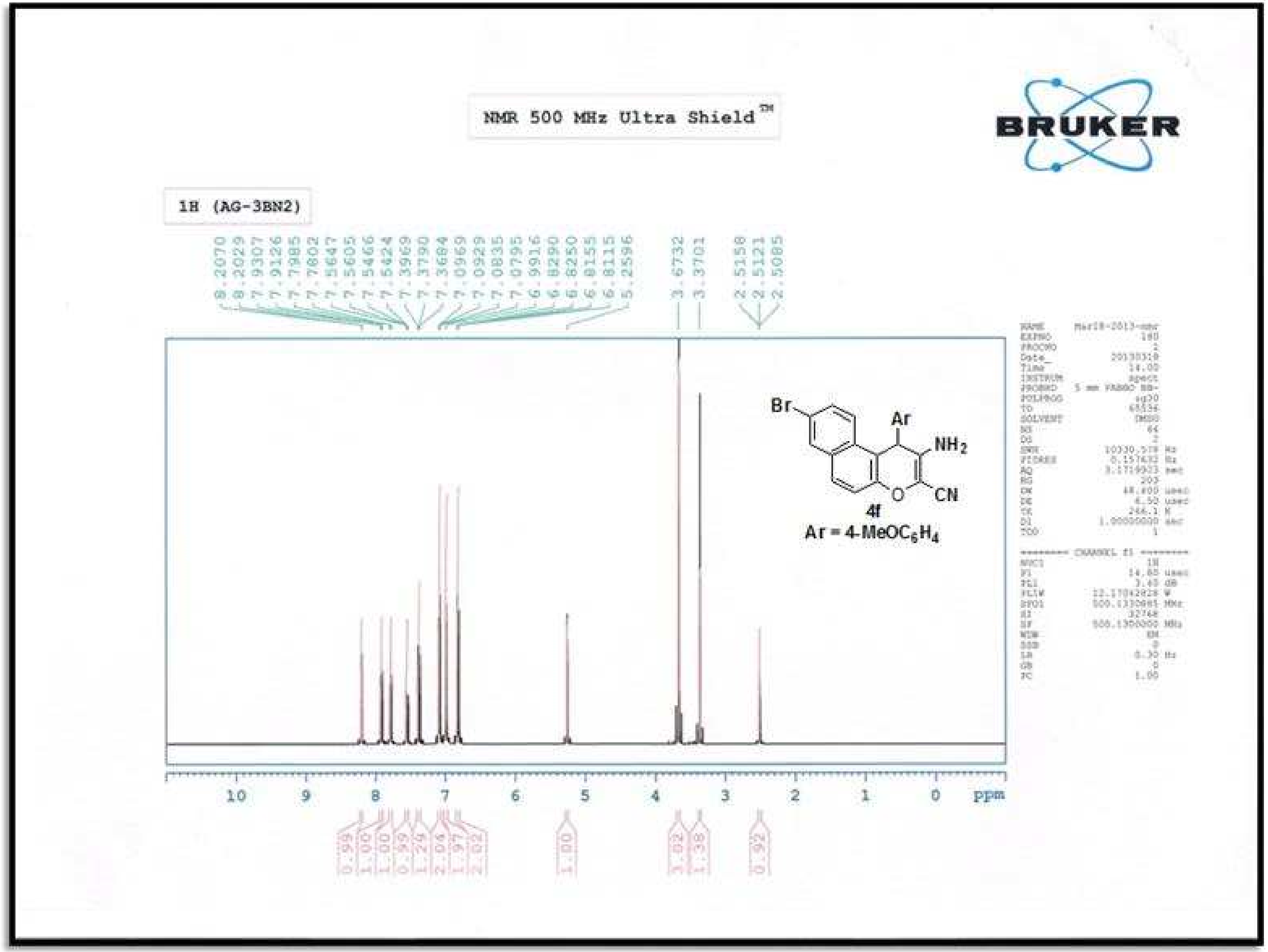
NMR 500 MHz Ultra Shield™
BRUKER
1H (AG-3BN2)
8.2070
8.2029
7.9307
7.9126
7.7985
7.7802
7.5647
7.5605
7.5466
7.5424
7.3969
7.3790
7.3684
7.0969
7.0929
7.0835
7.0795
6.9916
6.8290
6.8250
6.8155
6.8115
5.2596
3.6732
3.3701
2.5158
2.5121
2.5085
Br
Ar
NH2
O
CN
4f
Ar = 4-MeOC6H4
10
9
8
7
6
5
4
3
2
1
0
ppm
0.99
1.00
1.00
0.99
1.29
2.04
1.97
2.02
1.00
3.02
1.38
0.92

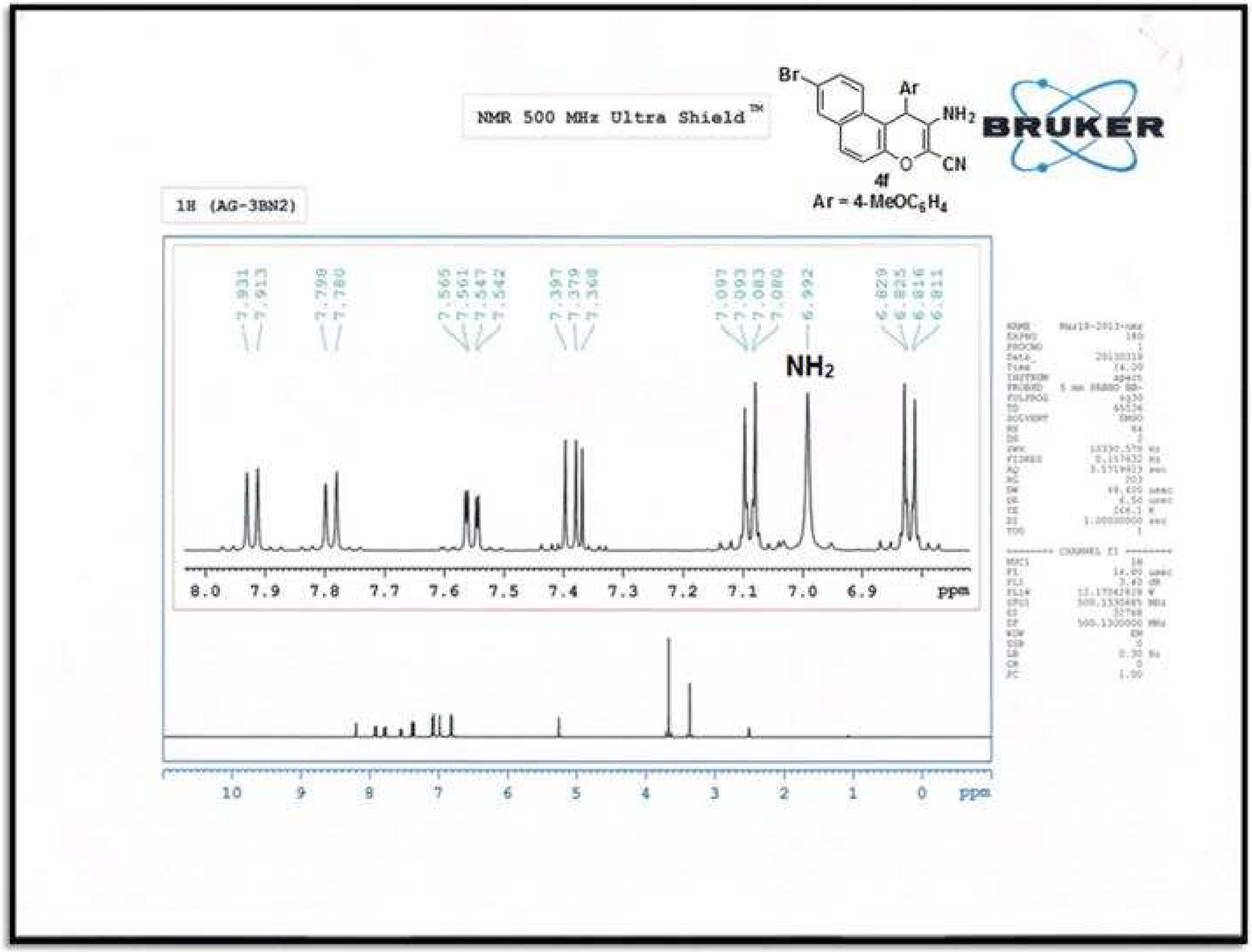
NMR 500 MHz Ultra Shield™
1H (AG-3BN2)
Br
Ar
NH2
CN
O
4f
Ar = 4-MeOC6H4
BRUKER
NH2
7.931
7.913
7.798
7.780
7.565
7.561
7.547
7.542
7.397
7.379
7.368
7.097
7.093
7.083
7.080
6.992
6.829
6.825
6.816
6.811
8.0
7.9
7.8
7.7
7.6
7.5
7.4
7.3
7.2
7.1
7.0
6.9
ppm
10
9
8
7
6
5
4
3
2
1
0
ppm

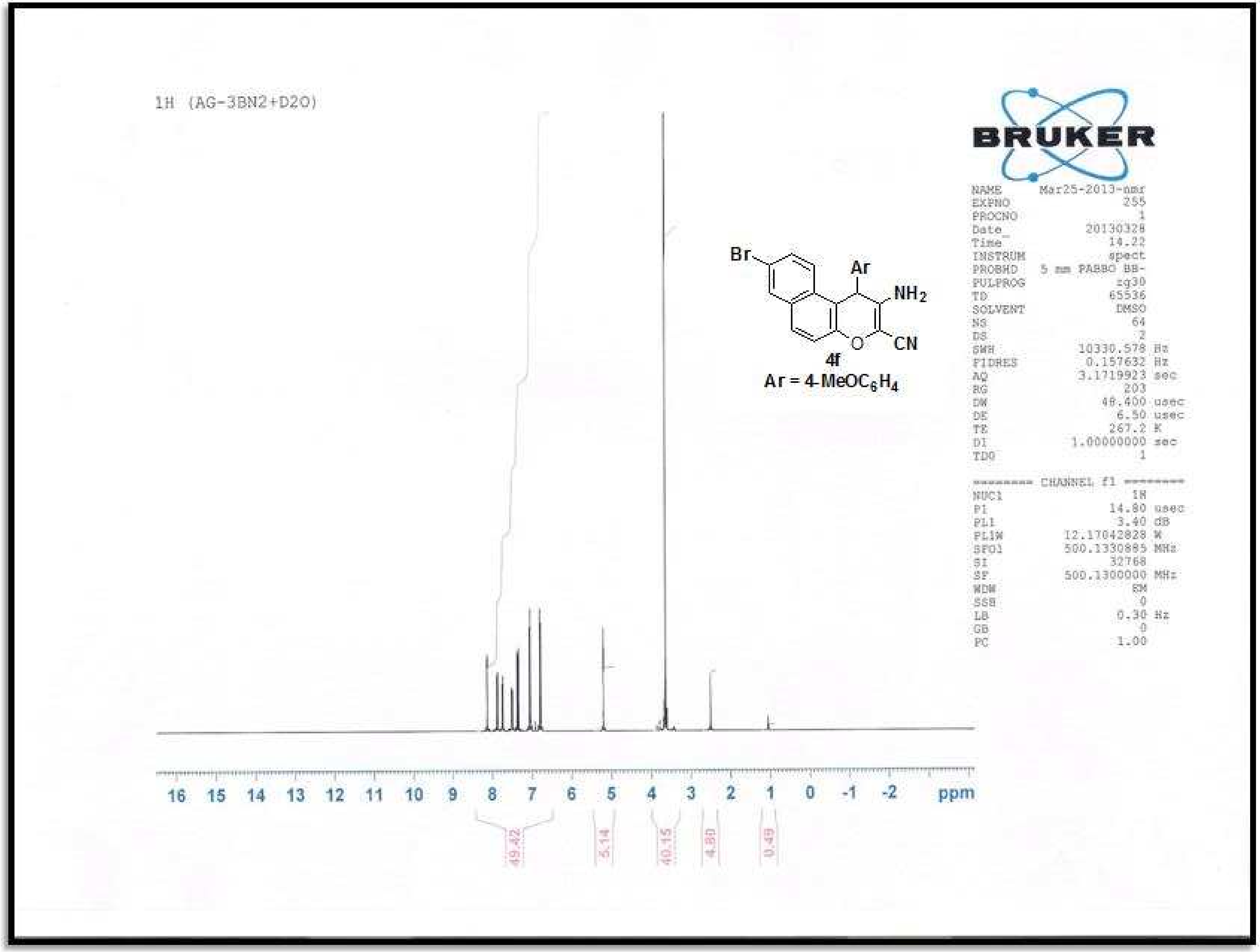
1H (AG-3BN2+D2O)
Br
Ar
NH2
CN
O
4f
Ar = 4-MeOC6H4
BRUKER
NAME Mar25-2013-nmr
EXPNO 255
PROCNO 1
Date_ 20130328
Time 14.22
INSTRUM spect
PROBHD 5 mm PABBO BB-
PULPROG zg30
TD 65536
SOLVENT DMSO
NS 64
DS 2
SWH 10330.578 Hz
FIDRES 0.157632 Hz
AQ 3.1719923 sec
RG 203
DW 48.400 usec
DE 6.50 usec
TE 267.2 K
D1 1.00000000 sec
TD0 1
======== CHANNEL f1 ========
NUC1 1H
P1 14.80 usec
PL1 3.40 dB
PL1W 12.17042828 W
SFO1 500.1330885 MHz
SI 32768
SF 500.1300000 MHz
WDW EM
SSB 0
LB 0.30 Hz
GB 0
PC 1.00
16 15 14 13 12 11 10 9 8 7 6 5 4 3 2 1 0 -1 -2 ppm
49.42
5.14
40.15
4.80
0.49

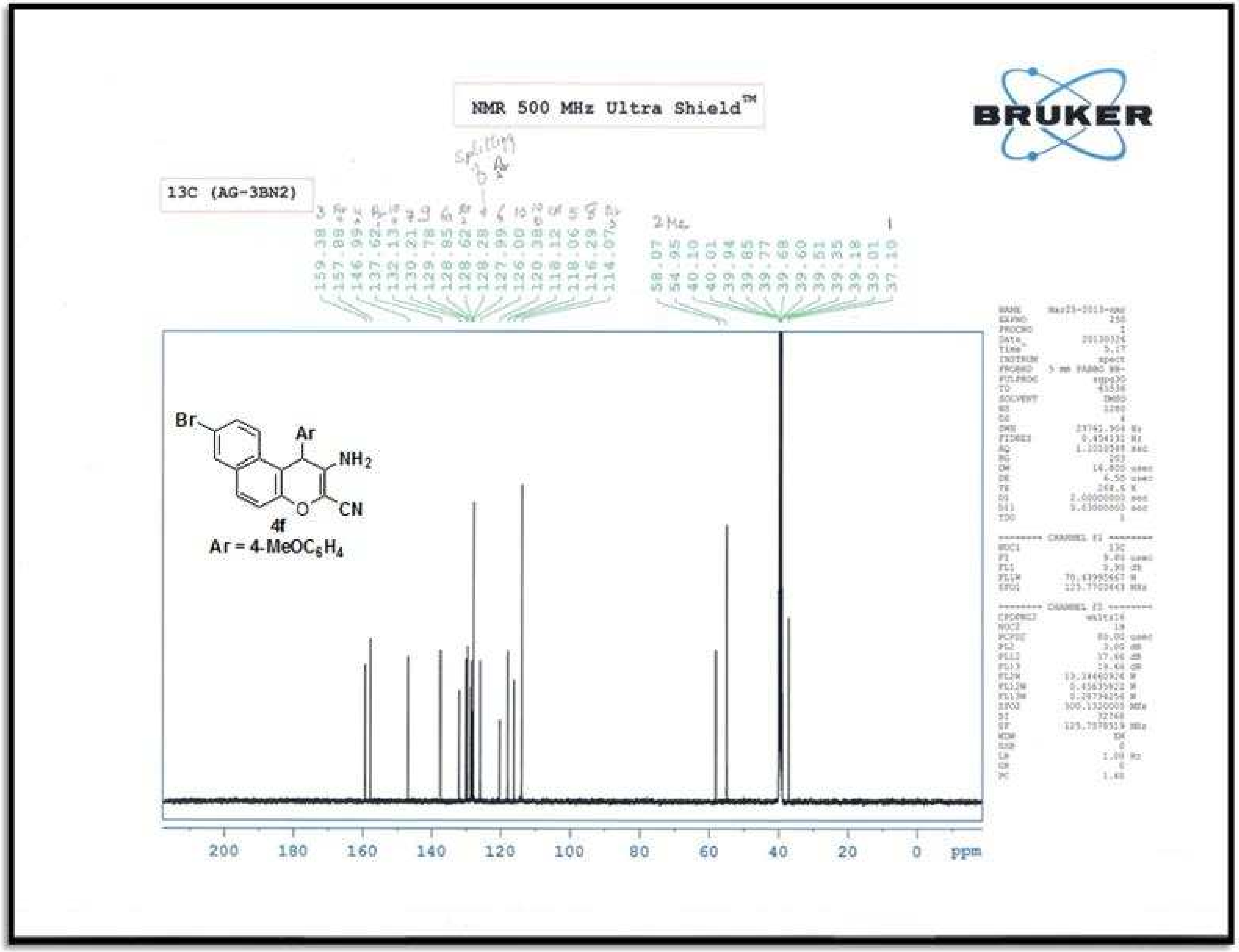
NMR 500 MHz Ultra Shield™
BRUKER
13C (AG-3BN2)
159.38
157.88
146.99
137.62
132.13
130.21
129.78
128.85
128.62
128.28
127.99
126.00
120.38
118.12
118.06
116.29
114.07
58.07
54.95
40.10
40.01
39.94
39.85
39.77
39.68
39.60
39.51
39.35
39.18
39.01
37.10
Br
Ar
NH2
CN
O
4f
Ar = 4-MeOC6H4
200
180
160
140
120
100
80
60
40
20
0
ppm

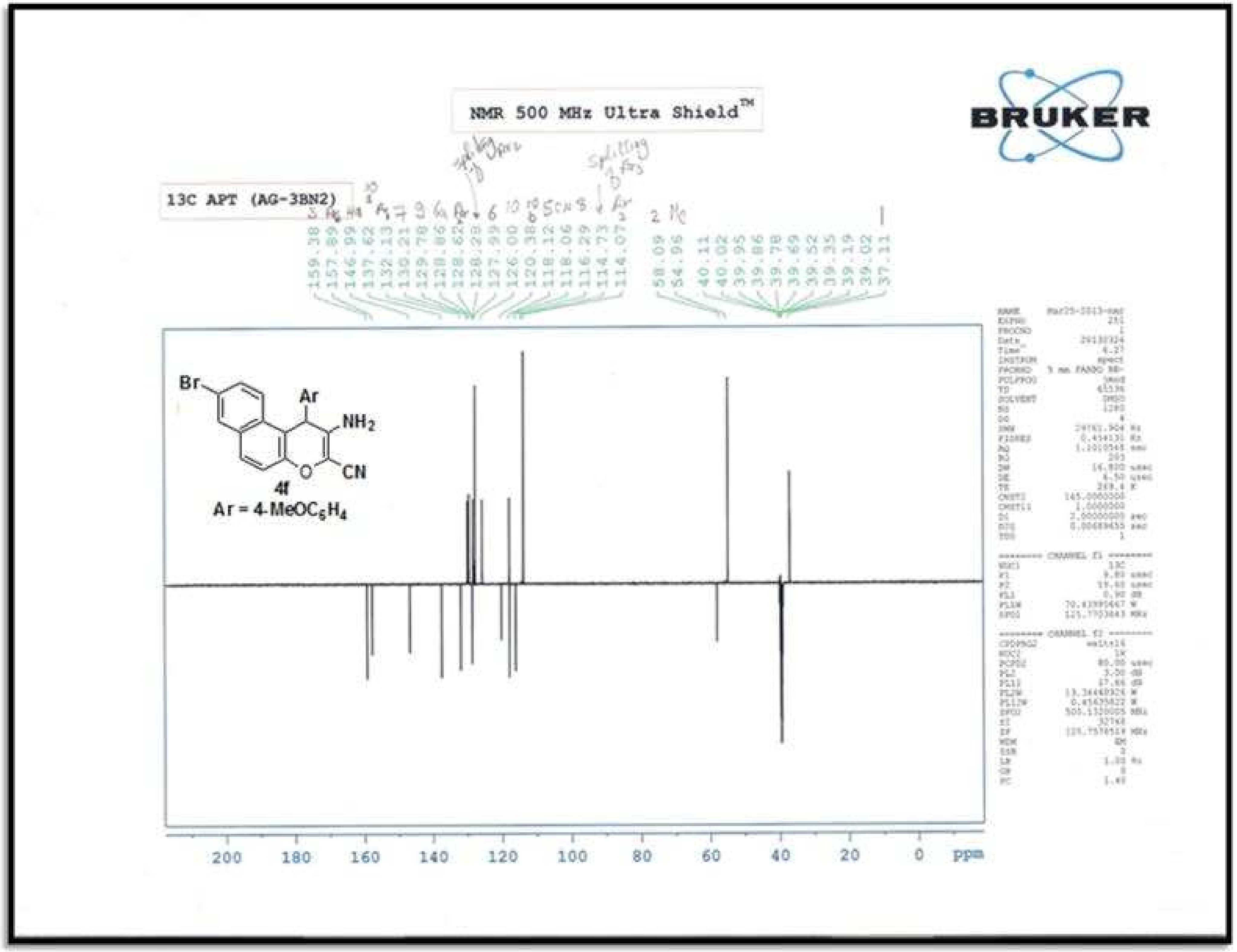
NMR 500 MHz Ultra Shield™
BRUKER
13C APT (AG-3BN2)
159.38
157.89
146.99
137.62
132.13
130.21
129.78
128.86
128.62
128.28
127.99
126.00
120.38
118.12
118.06
116.29
114.73
114.07
58.09
54.96
40.11
40.02
39.95
39.86
39.78
39.69
39.52
39.35
39.19
39.02
37.11
Br
Ar
NH2
CN
O
4f
Ar = 4-MeOC6H4
200
180
160
140
120
100
80
60
40
20
0
ppm

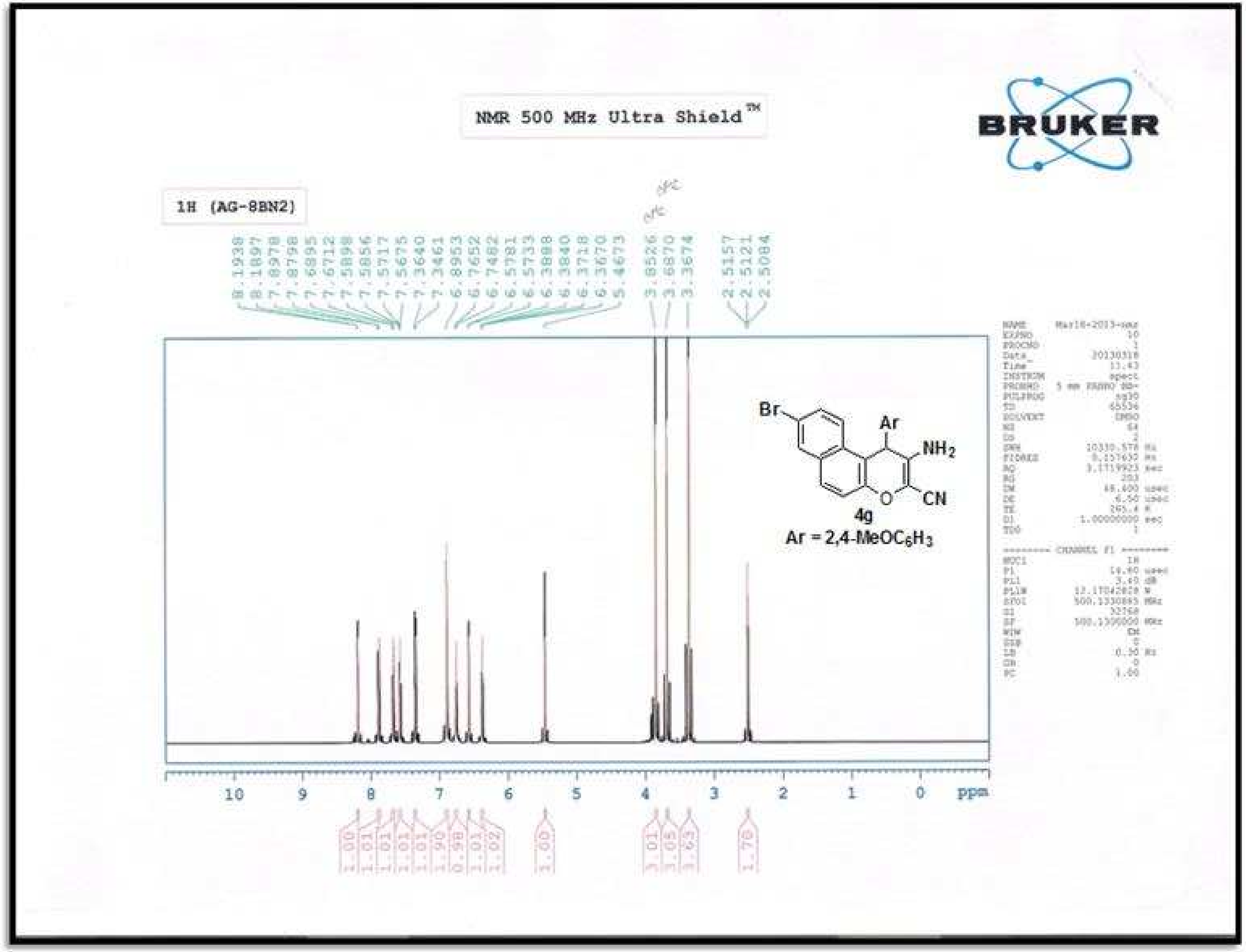
NMR 500 MHz Ultra Shield™
BRUKER
1H (AG-8BN2)
8.1938
8.1897
7.8978
7.8798
7.6895
7.6712
7.5898
7.5856
7.5717
7.5675
7.3640
7.3461
6.8953
6.7652
6.7482
6.5781
6.5733
6.3888
6.3840
6.3718
6.3670
5.4673
3.8526
3.6870
3.3674
2.5157
2.5121
2.5084
Br
Ar
NH2
O
CN
4g
Ar = 2,4-MeOC6H3
10 9 8 7 6 5 4 3 2 1 0 ppm
1.00 1.01 1.01 1.01 1.01 1.90 0.98 1.01 1.02
1.00
3.01 3.05 3.63
1.70

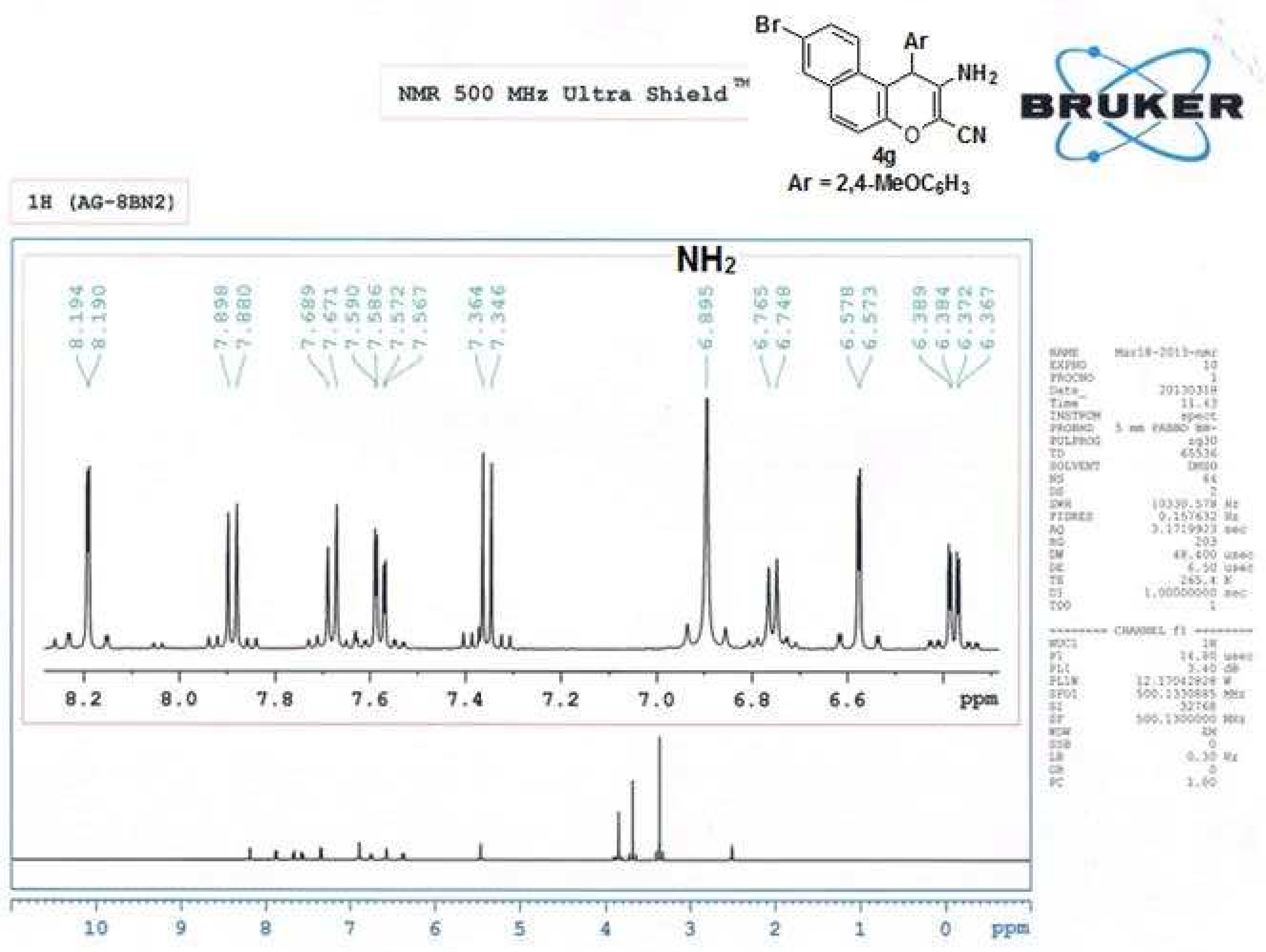
NMR 500 MHz Ultra Shield™
BRUKER
Br
Ar
NH2
CN
O
4g
Ar = 2,4-MeOC6H3
1H (AG-8BN2)
NH2
8.194
8.190
7.898
7.880
7.689
7.671
7.590
7.586
7.572
7.567
7.364
7.346
6.895
6.765
6.748
6.578
6.573
6.389
6.384
6.372
6.367
ppm

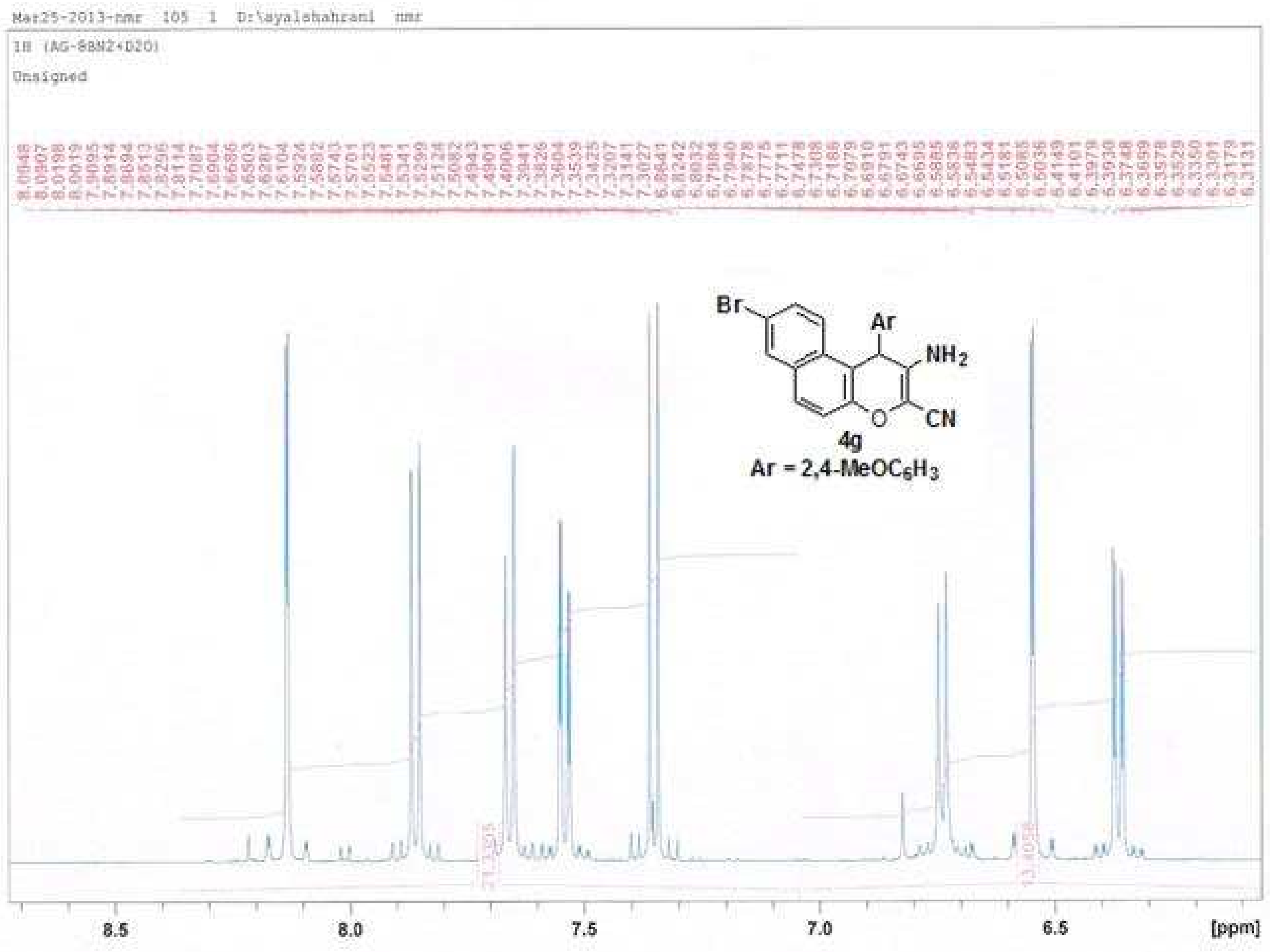

Mar25-2013-nmr 105 1 D:\ayalshahrani nmr
1H (AG-8BN2+D2O)
Unsigned
8.0948 8.0907 8.0198 8.0019 7.9095 7.8914 7.8694 7.8513 7.8296 7.8114 7.7087 7.6904 7.6686 7.6503 7.6287 7.6104 7.5924 7.5882 7.5743 7.5701 7.5523 7.5481 7.5341 7.5299 7.5124 7.5082 7.4943 7.4901 7.4006 7.3941 7.3826 7.3604 7.3539 7.3425 7.3207 7.3141 7.3027 6.8641 6.8242 6.8032 6.7984 6.7940 6.7878 6.7775 6.7711 6.7478 6.7308 6.7186 6.7079 6.6910 6.6791 6.6743 6.6695 6.5885 6.5838 6.5483 6.5434 6.5181 6.5085 6.5036 6.4149 6.4101 6.3979 6.3930 6.3748 6.3699 6.3578 6.3529 6.3350 6.3301 6.3179 6.3131
Br
Ar
NH2
O
CN
4g
Ar = 2,4-MeOC6H3
8.5
8.0
7.5
7.0
6.5
[ppm]

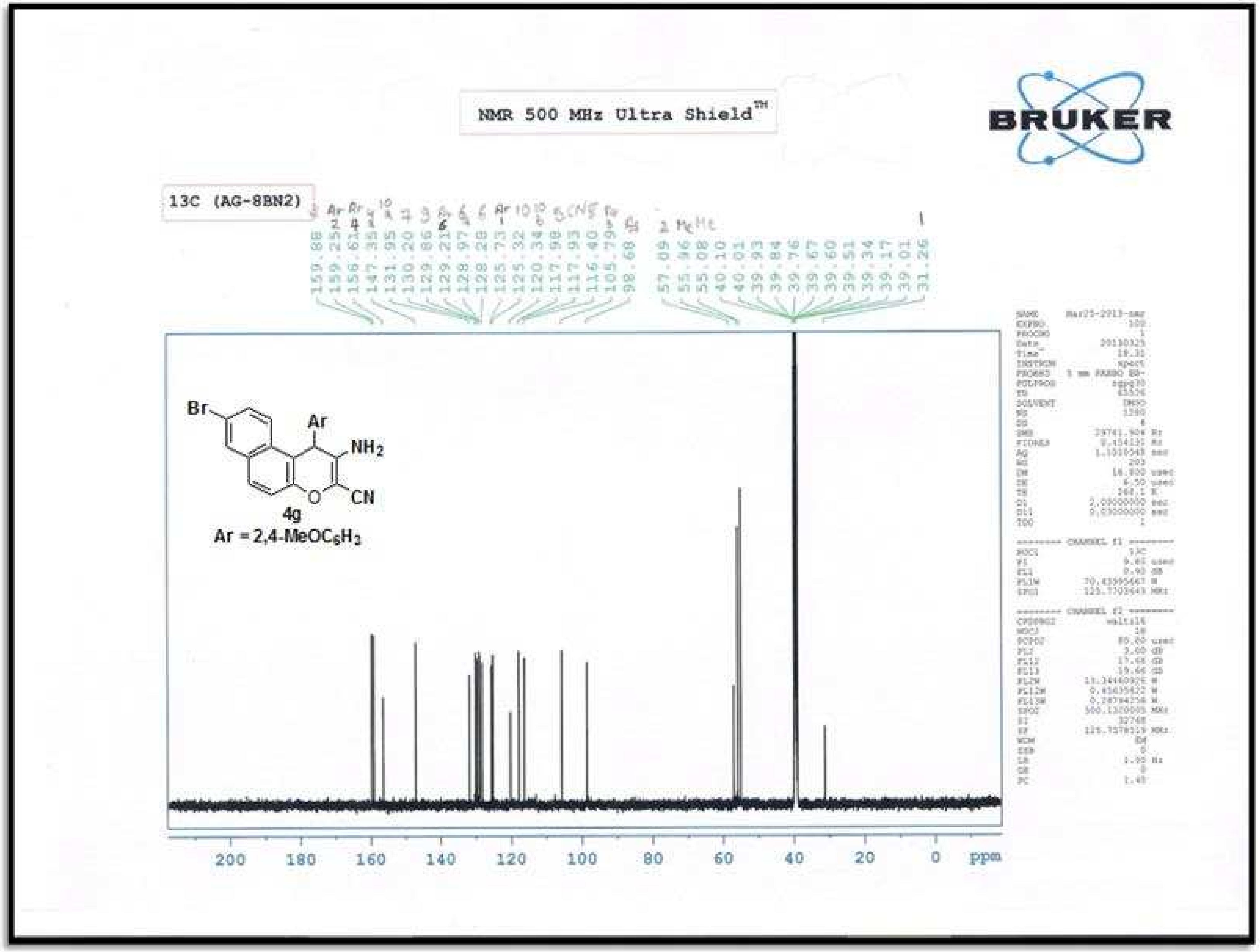
NMR 500 MHz Ultra Shield™
BRUKER
13C (AG-8BN2)
159.88
159.25
156.61
147.35
131.95
130.20
129.86
129.21
128.97
128.28
125.73
125.32
120.34
117.98
117.93
116.40
105.79
98.68
57.09
55.96
55.08
40.10
40.01
39.93
39.84
39.76
39.67
39.60
39.51
39.34
39.17
39.01
31.26
Br
Ar
NH2
CN
O
4g
Ar = 2,4-MeOC6H3
200
180
160
140
120
100
80
60
40
20
0
ppm

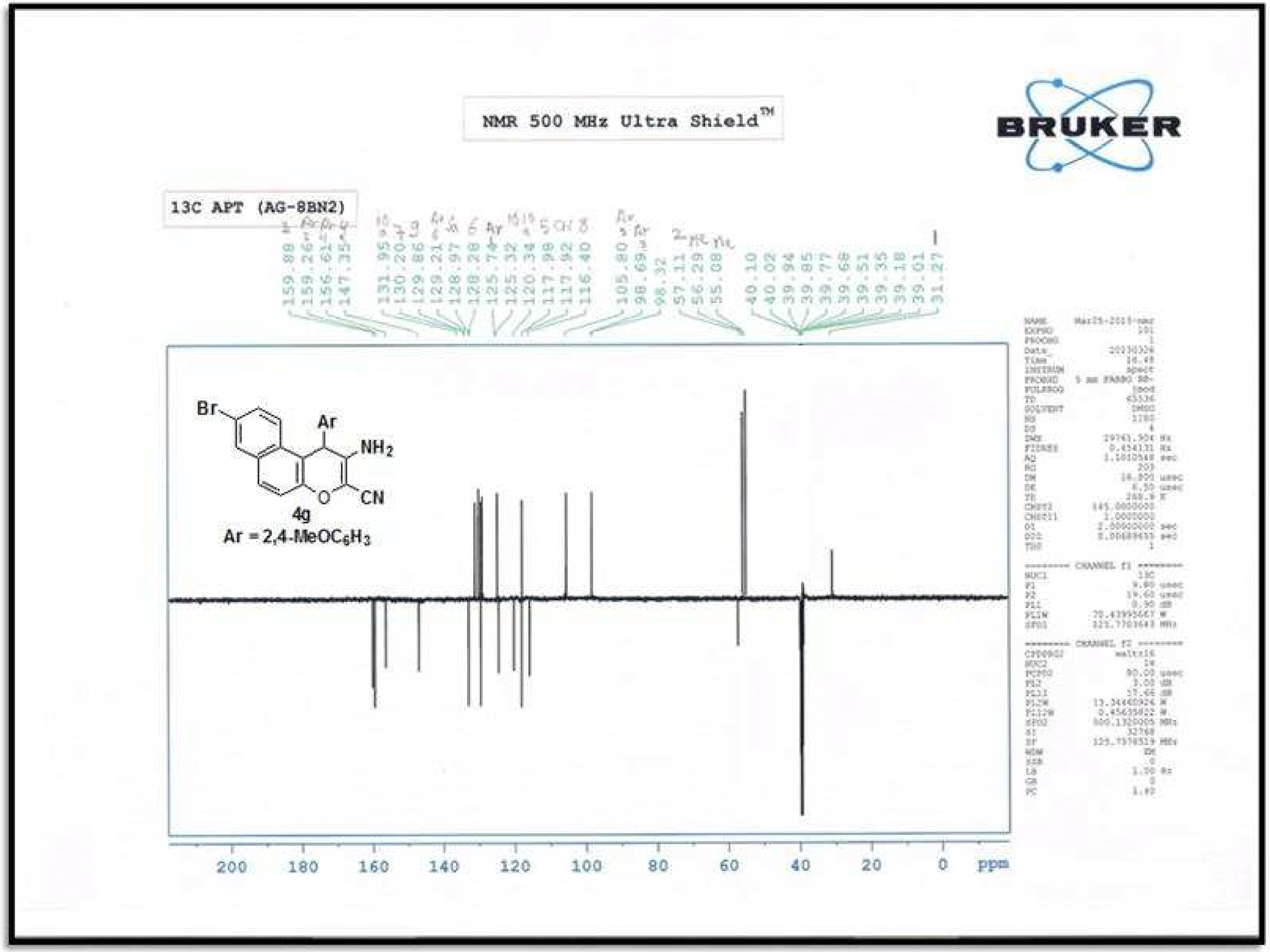
NMR 500 MHz Ultra Shield™
BRUKER
13C APT (AG-8BN2)
159.88
159.26
156.61
147.35
131.95
130.20
129.86
129.21
128.97
128.28
125.74
125.32
120.34
117.98
117.92
116.40
105.80
98.69
98.32
57.11
56.29
55.08
40.10
40.02
39.94
39.85
39.77
39.68
39.51
39.35
39.18
39.01
31.27
Br
Ar
NH2
CN
O
4g
Ar = 2,4-MeOC6H3
200
180
160
140
120
100
80
60
40
20
0
ppm

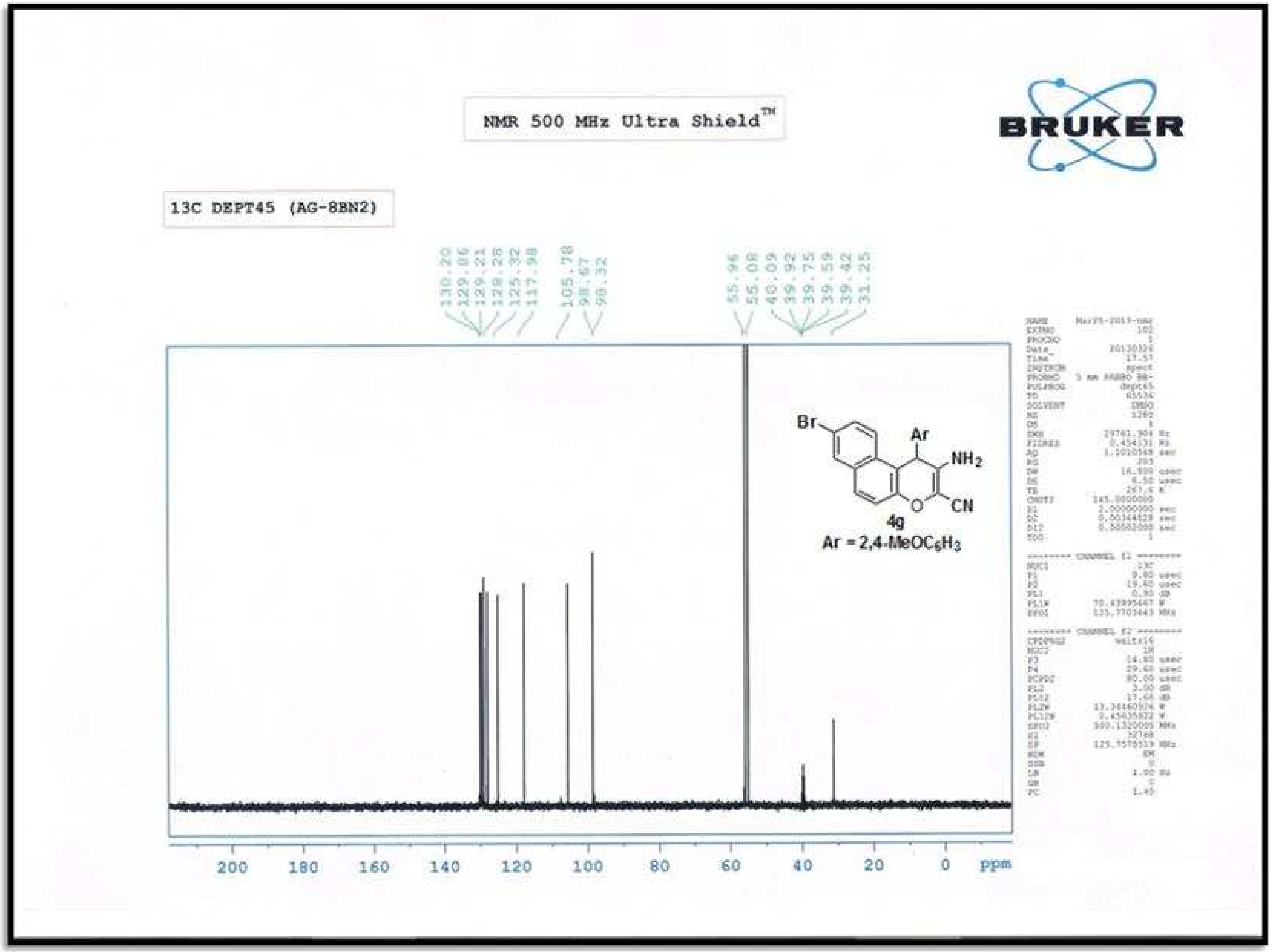
NMR 500 MHz Ultra Shield™
BRUKER
13C DEPT45 (AG-8BN2)
130.20
129.86
129.21
128.26
125.32
117.98
105.78
98.67
98.32
55.96
55.08
40.09
39.92
39.75
39.59
39.42
31.25
Br
Ar
NH2
O
CN
4g
Ar = 2,4-MeOC6H3
200
180
160
140
120
100
80
60
40
20
0
ppm
